# Design, Synthesis, and SAR of Novel 2-Glycinamide Cyclohexyl Sulfonamide Derivatives against *Botrytis cinerea*

**DOI:** 10.3390/molecules23040740

**Published:** 2018-03-23

**Authors:** Nan Cai, Caixiu Liu, Zhihui Feng, Xinghai Li, Zhiqiu Qi, Mingshan Ji, Peiwen Qin, Wasim Ahmed, Zining Cui

**Affiliations:** 1 Department of Pesticide Science, Plant Protection College, Shenyang Agricultural University, Shenyang 110866, Liaoning, China; ladycainan@126.com (N.C.); LCX14714027@163.com (C.L.); zhihuige950820@163.com (Z.F.); syqizhiqiu@sina.com (Z.Q.); jimingshan@163.com (M.J.); qinpeiwen08@sina.com (P.Q.); 2 State Key Laboratory for Conservation and Utilization of Subtropical Agro-bioresources, Integrative Microbiology Research Centre, Guangdong Province Key Laboratory of Microbial Signals and Disease Control, South China Agricultural University, Guangzhou 510642, Guangdong, China; wasimahmed1485@gmail.com

**Keywords:** β-aminosulfonamide, glycinamide, *N*-alkylation reaction, fungicidal activity, *Botrytis cinerea*

## Abstract

*N*-(2-trifluoromethyl-4-chlorophenyl)-2-oxocyclohexyl sulfonamide (chesulfamide) is in the limelight as a novel fungicide, and has fungicidal activity against *Botrytis cinerea*. For exploring more novel structures, 33 new compounds were synthesized by *N*-alkylation and acid–amine coupling reactions with chesulfamide as the core moiety, and their structures were characterized and established by ^1^H-NMR, ^13^C-NMR, MS, and elemental analysis. The structure of (1*R*,2*S*)-2-(2-(*N*-(4-chloro-2-trifluoromethylphenyl)sulfamoyl)-cyclohexylamino)-*N*-(2-trifluoromethylphenyl) acetamide (**II-19**) was defined by X-ray single crystal diffraction. The in vivo and in vitro fungicidal activities against *B. cinerea* were evaluated. The bioassay results of mycelial growth demonstrated that most compounds exhibited excellent inhibitory activity against *B. cinerea* at 50 μg mL^−1^, and 7 compounds showed lower EC_50_ values than boscalid (EC_50_ = 4.46 μg mL^−1^) against *B. cinerea* (CY-09). In cucumber pot experiment, the inhibitory rates of four compounds (**II-4**, **II-5**, **II-12**, and **II-13**) against *B. cinerea* were 90.48, 93.45, 92.86, and 91.07, which were better than cyprodinil (88.69%), the best performing of all controls. In tomato pot experiment, the control efficacy of two analogs (**II-8** and **II-15**) were 87.98 and 87.97% at 200 μg mL^−1^, which were significantly higher than boscalid (78.10%). Most compounds have an excellent fungicidal effect on *B. cinerea*, with potential as a lead compound for developing new pesticides.

## 1. Introduction

Plant-pathogenic fungi, with the characteristics of diversity and wide distribution, are causing a destructive economic and humanitarian problem, which threatens food security and biodiversity [1,2,3,4,5]. *B. cinerea*, one of the important representative fungi, has a necrotic lifestyle, and attacks over 200 major dicotyledonous plants in temperate and subtropical regions, causing soft rotting of all aerial plant parts, as well as decaying vegetables, fruits, and flowers after harvesting [6,7]. Despite the availability of many fungicides to control it, many of them have failed due to the genetic plasticity [6]. The reality is that the resistance in various parts of the world is not adequate [8,9,10,11]. The rapid development of the resistance of *Botrytis cinerea* and the ongoing emergence of resistant strains attracted our attention for the development of new fungicides.

In current research and development of fungicides, amide and sulfonamide compounds are the center of attraction. Compounds with amide structures have been reported to have broad pharmaceutical activities against bacteria [12], virus [13], inflammation [14], tumors [15] diabetes [16], and also showed excellent fungicidal activity in agrochemicals. The molecular size and charge distribution characteristics of sulfonamide are similar to amide. Additionally, reasonable introduction of sulfonyl can increase metabolic stability of the functional molecule and prolong the duration of action by blocking the metabolic instability site and improve the bioavailability [17]. Compounds with active sulfonamide and amide substructure may have a new mechanism of action. Therefore, while designing the new molecular structures, we introduced both sulfonamide and amide groups to examine the fungicidal activity. 

**Figure 1:**
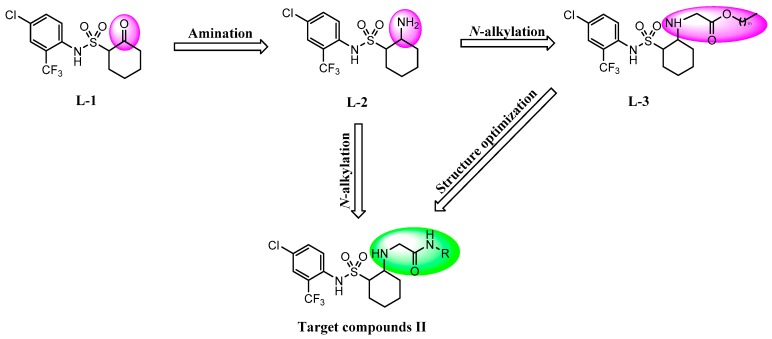
The designed strategy for title compounds **II**.

**Figure 2:**
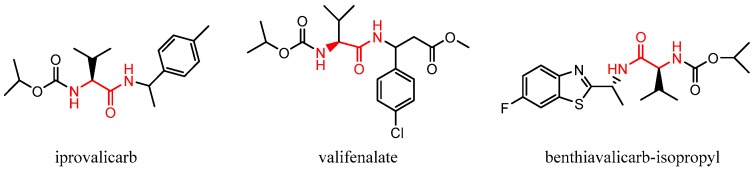
Chemical structures of some fungicides.

**Figure 3:**
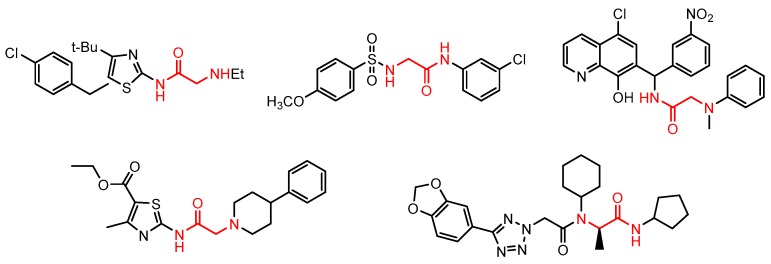
Chemical structures of several *N*-substituted glycinamide compounds.

**Scheme 1:**
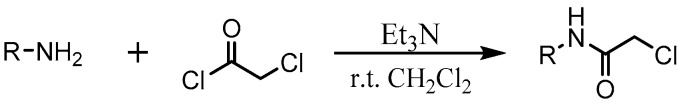
Synthesis of substituted chloroacetamides **I**.

**Scheme 2:**
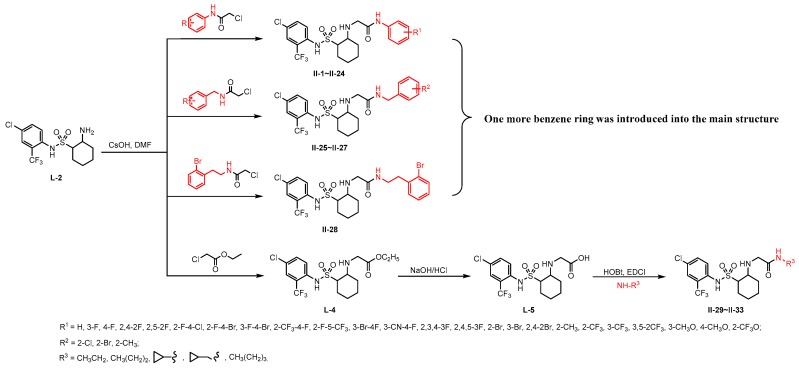
Synthesis of *N*-substituted glycinamide derivatives of 2-amino-cyclohexylsulfonamides **II**.

In our previous work, we found that chesulfamide (L-1) [18,19,20,21] can effectively control *B. cinerea* and *Corynespora cassiicola* with strong preventive, therapeutic and osmotic activity [18]. It is worth mentioning that chesulfamide has a unique mechanism of action, which includes (1) acting on mycelium cell membrane, (2) reducing the content of DNA and polysaccharide in mycelium and having certain binding effects with DNA, and (3) inducing disease resistance of plants [18]. Using chesulfamide as the lead compound, we reduced the oxo group to the hydroxyl group [22,23] to make related esters [23,24], and replaced the hydroxyl group by the amino group [25], which enabled the introduction of aromatic and heterocyclic rings [26]. After analyzing the structure–activity relationship (SAR), we found that converting carbonyl to amino group (**L-2**) improved the reactivity, made the structure more variable, and enhanced the anticipation of introducing diversified active structures into the main structure. Afterwards, the amino group was transformed to aminoacetate (**L-3**), a glycine derivative structure, and we attempted to exploit the high affinity of glycine for organisms to increase biological activity, but unfortunately we did not achieve the desired bioassay results [25]. In order to get improved activity, aminoacetate was converted into aminoacetamide with alkyl, phenethyl, benzyl, and substituted benzene ring (Figure 1). Aminoacetamide structure was found in the listed fungicides, such as iprovalicarb, valifenalate and benthiavalicarb-isopropyl (Figure 2) [27], and previous reports also showed that compounds containing this structure have outstanding pharmacological activity [26,28,29,30,31]. Therefore, among the structures, nitrogen was functionalized as alkyl, sulfonyl, benzene, piperidine, and acylated tetrazole, in order to get more diverse products (Figure 3), but the cyclohexylsulfonamide structure that we linked in target compounds had not yet been done before. Here, we designed and synthesized a series of 2-glycinamide cyclohexyl sulfonamide derivatives. Their comprehensive fungicidal activity against *B. cinerea* were evaluated, and the structure–activity relationship was analyzed. The synthetic routes of intermediates and target compounds were shown in Scheme 1 and Scheme 2, respectively.

## 2. Result and Discussion

### 2.1. Chemistry

The synthetic routes for the target compounds are shown in Scheme 2. Substituted chloroacetamide (Intermediate I) was synthesized by the reaction of amines and chloroacetyl chloride in dichloromethane (CH_2_Cl_2_) as solvent in the presence of triethylamine (Et_3_N). The target compounds **II-1** to **II-28** were obtained by the nitrogen alkylation reaction of *N*-(2-trifluoromethyl-4-chlorophenyl)-2-amino cyclohexyl sulfonamide (the key intermediate **L-2**) with intermediate I in the presence of cesium hydroxide (CsOH) in *N,N*-dimethylformamide (DMF). *N*-(2-Trifluoromethyl-4-chlorophenyl)-2-ethoxyacylmethyl amino cyclohexyl sulfonamide **L-4** was transformed to *N*-(2-trifluoromethyl-4-chlorophenyl)-2-carboxymethyl amino cyclohexyl sulfonamide L-5 by consecutive hydrolysis and acidification reaction. Then, the free acid of **L-5** was coupled with different amines in presence of 1-hydroxybenzotriazole (HOBt) and *N*-(3-dimethylaminopropyl)-*N*′-ethylcarbodiimide (EDCI) at 0 °C to obtain the target compounds **II-29** to **II-33**.

By comparing the two methods used to synthesize the target compounds, we found the later method resulted in higher yields and easier purification. Prior to this, we also tried to use potassium *tert*-butoxide [32], magnesium methoxide, and calcium chloride [33] as catalysts, trying to promote the amidation reaction of **L-4** with amines, but we abandoned this method finally, because of its low yield.

**Figure 4:**
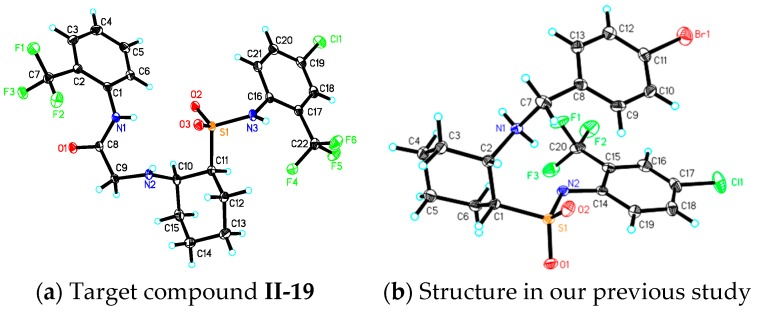
X-ray crystal structures (red represents oxygen, green is fluorin, light blue stands for hydrogen, deep blue symbolizes nitrogen).

The structures of the target compounds were characterized by ^1^H-NMR, ^13^C-NMR, MS and elemental analysis. Twenty two of them were published in a Chinese patent [34]. The ^1^H-NMR and ^13^C-NMR spectra are available in Supplementary Data. Additionally, the structure of **II-19** was confirmed by X-ray single crystal diffraction (Figure 4a). The molecular structure of **II-19** existed as a chair conformation, the sulfonamide moiety on the equatorial bond position and the glycinamide moiety was linked to cyclohexane by an axial bond. The first chiral carbon atom (C11) had the *S* configuration and the second chiral carbon atom (C10) had the *R* configuration. Based on the structural similarities of all the target compounds, it was supposed that all the compounds had the same configuration, the same as **II-19**. Interestingly, a reported compound (Figure 4b) with similar structure showed proton transfer [25], which was not observed in this study. We found that the H atom on SO_2_–NH (N2) was trapped by the N atom (N1) attached to the cyclohexane, whereas compound **II-19** did not present this behavior. Since the amino group (N2) linked to the acetamido structure (which probably exerts an electron withdrawing effect) may acquire a weaker basicity, the lack of proton migration phenomenon appears to be justified.

### 2.2. Biological Activity and Structure–Activity Relationship Study

The mycelium inhibition of all target compounds against *B. cinerea* was initially tested at 50 μg mL^−1^, and most of them exhibited significant fungicidal activity similar or superior to control (Table 1 and Table 2). The inhibitory rate of **II-4**, **II-12**, and **II-13** (90.66%, 90.41%, and 90.90%) was higher than boscalid (89.18%) against CY-09. Next, we further carried out a concentration gradient test on CY-09 and calculated the EC_50_ values (Table 1). The result showed that the EC_50_ values of 14 compounds were less than procymidone (EC_50_ = 10.31 μg/mL). **II-5**, **II-6**, **II-19**, **II-26**, and **II-28** had higher fungicidal activity than all positive control, with the EC_50_ values of 3.38, 3.38, 3.26, 3.40, and 3.57 μg mL^−1^. **II-4**, **II-11**, **II-18**, **II-30**, **II-31**, and **II-33** also showed outstanding fungicidal activity, which were significantly better than carbendazim, and substantially the same or better than the other four. Based on the results, 9 compounds were tested on HLD-15 and DL-11 in the same manner, and further confirmed the high fungicidal activity again (Table 2).

The compounds with outstanding fungicidal activity in vitro tests also demonstrated excellent control effect and stable performance in cucumber and tomato pot experiments (Table 3 and Table 4). Meanwhile, there was no phytotoxicity occurred during the tests. **II-4**, **II-5**, **II-12** and **II-13**, which showed superb activity in vitro tests, had higher inhibition rates (90.48%, 93.45%, 92.86%, and 91.07%) in cucumber pot test than carbendazim (59.52%), procymidone (83.33%), boscalid (88.10%), pyrimethanil (82.14%), and cyprodinil (88.69%). In tomato pot test, the control efficacy of **II-4**, **II-5**, **II-7**, **II-8**, **II-15**, and **II-33** were 67.19%, 68.43%, 73.83%, 80.14%, 76.96%, and 72.02% at the concentration of 500 μg mL^−1^, which were similar to boscalid (77.05%), the best performing amongst the controls. Subsequently, we selected 9 compounds to test, and reduced the concentration to 200 μg mL^−1^. The results showed that the test compounds basically maintained high control effect, and the control efficacy of **II-4**, **II-5, II-8**, and **II-15** (78.51%, 78.91%, 87.98%, and 87.97%) were better than all control agents. Due to the growth status of tomato seedlings and the pathogenicity of spores at different stages, the two batches of results did not show a directly proportional to the concentration, but overall, results showed the same trend, that specific compounds showed higher effect than control.

Accordingly, by analyzing the experimental results, we determined the relationship between chemical structures and fungicidal activity as (1) the compounds with substituents on benzene ring were better than that without substituents (**II-1**), and fluorine and bromine atoms on benzene ring showed better activity, while fluorine was the best; (2) Substituents on phenyl ring were more active at *para*- and *ortho*-position than at *meta*-position. Activity was generally low with substituents only at *meta*-position (**II-2**, **II-16**, **II-20**, **II-22**) and with little activity at both 3- and 5-position (**II-21**); (3) Compared **II-3**, **II-4**, **II-5**, **II-13** and **II-14**, we found that there was no significant difference between di- and multi-substituted compounds, but it was obviously better than the mono-substituted; (4) The halogen atoms on the benzene ring were superior to the electron-donating groups (CH_3_O-); (5) Comparison of **II-18** and **II-27** or comparison among **II-15**, **II-26**, and **II-28** showed that extension of the carbon chain between the amine group and benzene ring increased in vitro fungicidal activity; (6) By comparing **II-29** to **II-33**, it can be concluded that increasing the length of the alkyl carbon chain enhanced fungicidal activity, and the cyclic structure (**II-31**) had a higher activity than the chain structure (**II-30**) of the same carbon number. In total, compounds with substituted benzene ring showed more stable and highly fungicidal activity than compounds with alkyl substituents.

Based on overall bioassay results, the compounds had a stronger fungicidal effect on in vitro assays and cucumber pot experiments (infected with mycelium) compared to tomato pot experiments (infected with spraying spores), that is to say, the compounds had slightly better inhibitory effects on mycelium than spores.

## 3. Materials and Method

### 3.1. Materials and Instrumentation

All solvents and reagents were commercially available for analytical reagent (AR) grade and dried prior to use. Column chromatography (Silica gel: 200–300 mesh) was used to purify target compounds. The melting points were determined by the X-5 binocular microscope melting point apparatus (Beijing Tech Instrument Co. Ltd., Beijing, China). ^1^H-NMR and ^13^C-NMR spectra were recorded on a Bruker 600 MHz and 101 MHz spectrometer (Bruker, Karlsruhe, Germany), using dimethyl sulfoxide (DMSO-*d*_6_) as solvent and tetramethylsilane (TMS) as the internal standard. MS data were obtained on the 7000C Triple Quad GC/MS and 6460 Triple Quad LC/MS Mass Spectrometers (Agilent Technologies, Santa Clara, CA, USA).

### 3.2. Synthesis

#### 3.2.1. Synthesis of *N*-(2-Trifluoromethyl-4-chlorophenyl)-2-aminocyclohexyl Sulfonamide **L-2**

The synthesis of intermediate **L-2** was done according to the reported method [35].

#### 3.2.2. Synthesis of Substituted Chloroacetamide **I**

Under nitrogen atmosphere, dry CH_2_Cl_2_ (30 mL), amine (0.02 mol), and Et_3_N (0.05 mol) were added to a three-necked round bottom flask and stirred for 0.5 h, then chloroacetyl chloride dropped slowly and reacted for 3 h at room temperature. Then, the solution was washed with 2 mol L^−1^ hydrochloric acid (30 mL), saturated aq NaHCO_3_ solution (30 mL), and brine (40 mL), successively, then dried over anhydrous Na_2_SO_4_ and filtered. After evaporating CH_2_Cl_2_ in vacuum, the obtained crude product was refined by recrystallization using ethyl acetate/petroleum ether.

#### 3.2.3. Synthesis of 2-Glycinamide Cyclohexyl Sulfonamide Derivatives **II-1** to **II-28**

Under nitrogen atmosphere, DMF (10 mL), 4 Å molecular sieves (0.5 g), and CsOH (2 mmol) were placed in a three-necked round bottom flask and stirred for 10 min, then compound **L-2** (1.5 mmol) was added to the flask and reacted for 0.5 h. Hereafter, intermediate I (1.8 mmol) was slowly added at room temperature, and reaction was terminated according to TLC monitoring results [25,36]. The solution was filtered in vacuum and 100 mL distilled water added. Organic compounds were extracted with ether (3 × 120 mL), organic solvent was dried over anhydrous Na_2_SO_4_ and filtered. Solvent was evaporated in vacuum, and the residue was purified by silica gel column chromatography (petroleum ether/ethyl acetate as eluents). Finally, the samples were recrystallized to give target compounds with higher purity.

*(1R,2S)-2-(2-(N-(4-Chloro-2-trifluoromethylphenyl)sulfamoyl)-cyclohexylamino)-N-phenylacetamide* **II-1**. White solid, yield: 57.2%. mp 190–192 °C. ^1^H-NMR (600 MHz, DMSO-*d*_6_): *δ*(ppm) 10.13 (s, 1H, NH–C=O), 7.63–7.06 (m, 10H, NH + NH + C_6_H_3_ + C_6_H_5_), 3.65 (d, *J* = 16.0 Hz, 1H, CH–SO_2_), 3.50–3.39 (m, 2H, CH_2_), 3.29–3.23 (m, 1H, CH–N), 1.97–1.29 (m, 8H, 4CH_2_). ^13^C-NMR (101 MHz, DMSO-*d*_6_): *δ*(ppm) 168.46, 138.81, 133.14, 129.19, 128.02, 126.67, 126.28, 124.46, 123.87, 122.65, 120.84, 119.33 (2), 62.67, 54.56, 49.95, 27.79, 23.93, 22.01, 19.24. EIMS, *m*/*z* 490.2 [M]^+^. Elemental analysis for C_21_H_23_ClF_3_N_3_O_3_S: found C 51.66, H 4.58, N 8.39; calcd C 51.48, H 4.73, N 8.58.

*(1R,2S)-2-(2-(N-(4-Chloro-2-trifluoromethylphenyl)sulfamoyl)-cyclohexylamino)-N-(3-fluorophenyl)acetamide* **II-2**. White solid, yield: 62.4%. mp 179–180 °C. ^1^H-NMR (600 MHz, DMSO-*d*_6_): *δ*(ppm) 10.29 (s, 1H, NH–C=O), 7.67–6.89 (m, 9H, NH + NH + C_6_H_3_ + C_6_H_4_), 3.61 (d, *J* = 16.7 Hz, 1H, CH–SO_2_), 3.45–3.35 (m, 2H, CH_2_), 3.29–3.19 (m, 1H, CH–N), 2.00–1.29 (m, 8H, 4CH_2_). ^13^C-NMR (101 MHz, DMSO-*d*_6_): *δ*(ppm) 169.50, 163.33, 161.73, 140.56, 133.26, 130.89, 128.87, 126.83, 124.31, 122.50, 115.09, 110.22, 106.16, 105.99, 63.21, 54.28, 50.09, 27.98, 24.05, 21.90, 19.11. EIMS, *m*/*z* 508.4 [M]^+^. Elemental analysis for C_21_H_22_ClF_4_N_3_O_3_S: found C 49.82, H 4.15, N 8.37; calcd C 49.66, H 4.37, N 8.27.

*(1R,2S)-2-(2-(N-(4-Chloro-2-trifluoromethylphenyl)sulfamoyl)-cyclohexylamino)-N-(4-fluorophenyl)acetamide* **II-3**. White solid, yield: 56.5%. mp 157–158 °C. ^1^H-NMR (600 MHz, DMSO-*d*_6_): *δ*(ppm) 10.18 (s, 1H, NH–C=O), 7.66–7.15 (m, 9H, NH + NH + C_6_H_3_ + C_6_H_4_), 3.63 (d, *J* = 15.3 Hz, 1H, CH–SO_2_), 3.48–3.36 (m, 2H, CH_2_), 3.29–3.20 (m, 1H, CH–N), 1.95–1.30 (m, 8H, 4CH_2_). ^13^C-NMR (101 MHz, DMSO-*d*_6_): *δ*(ppm) 168.55, 159.27, 157.68, 135.25, 133.18, 128.28, 126.71, 124.41, 122.60, 121.01, 115.85 (2), 115.70 (2), 62.84, 54.46, 49.91, 27.84, 23.96, 21.97, 19.20. EIMS, *m*/*z* 508.4 [M]^+^. Elemental analysis for C_21_H_22_ClF_4_N_3_O_3_S: found C 49.38, H 4.61, N 8.52; calcd C 49.66, H 4.37, N 8.27.

*(1R,2S)-2-(2-(N-(4-Chloro-2-trifluoromethylphenyl)sulfamoyl)-cyclohexylamino)-N-(2,4-difluorophenyl)acetamide* **II-4**. White solid, yield: 30.3%. mp 145–146 °C. ^1^H-NMR (600 MHz, DMSO-*d*_6_): *δ*(ppm) 9.95 (s, 1H, NH–C=O), 7.88–7.07 (m, 6H, C_6_H_3_ + C_6_H_3_), 6.59–6.38 (m, 2H, NH + NH), 3.76 (s, 1H, CH–SO_2_), 3.58–3.41 (m, 2H, CH_2_), 3.29–3.21 (m, 1H, CH–N), 1.94–1.30 (m, 8H, 4CH_2_). ^13^C-NMR (101 MHz, DMSO-*d*_6_): *δ*(ppm) 170.05, 160.24, 158.62, 133.23, 131.71, 130.72, 128.66, 126.73, 124.30, 123.95, 122.49, 120.57, 114.17, 63.04, 54.09, 49.81, 27.97, 24.00, 22.03, 19.31. EIMS, *m*/*z* 526.4 [M]^+^. Elemental analysis for C_21_H_21_ClF_5_N_3_O_3_S: found C 47.72, H 3.89, N 8.16; calcd C 47.96, H 4.02, N 7.99.

*(1R,2S)-2-(2-(N-(4-Chloro-2-trifluoromethylphenyl)sulfamoyl)-cyclohexylamino)-N-(2,5-difluorophenyl)acetamide* **II-5**. White solid, yield: 57.9%. mp 95–97 °C. ^1^H-NMR (600 MHz, DMSO-*d*_6_): *δ*(ppm) 9.89 (s, 1H, NH–C=O), 8.01–7.13 (m, 6H, C_6_H_3_ + C_6_H_3_), 4.02 (m, 2H, NH + NH), 3.67 (s, 1H, CH–SO_2_), 3.53–3.43 (m, 2H, CH_2_), 3.35–3.27 (m, 1H, CH–N), 1.98–1.32 (m, 8H, 4CH_2_). ^13^C-NMR (101 MHz, DMSO-*d*_6_): *δ*(ppm) 169.12, 159.74, 157.09, 139.96, 133.65, 134.41, 128.96, 127.07, 123.95, 122.51, 116.66, 107.28, 101.23, 63.45, 54.09, 50.13, 28.09, 24.03, 22.01, 19.09. EIMS, *m*/*z* 526.3 [M]^+^. Elemental analysis for C_21_H_21_ClF_5_N_3_O_3_S: found C 48.17, H 4.28, N 7.75; calcd C 47.96, H 4.02, N 7.99.

*(1R,2S)-2-(2-(N-(4-Chloro-2-trifluoromethylphenyl)sulfamoyl)-cyclohexylamino)-N-(2-fluoro-4-chloro phenyl)acetamide* **II-6**. White solid, yield: 49.9%. mp 116–118 °C. ^1^H-NMR (600 MHz, DMSO-*d*_6_): *δ*(ppm) 10.27 (s, 1H, NH–C=O), 8.32–7.57 (m, 6H, C_6_H_3_ + C_6_H_3_), 6.92 (s, 2H, NH + NH), 3.68 (d, *J* = 15.4 Hz, 1H, CH–SO_2_), 3.52–3.40 (m, 2H, CH_2_), 3.27 (s, 1H, CH–N), 1.96–1.25 (m, 8H, 4CH_2_). ^13^C-NMR (101 MHz, DMSO-*d*_6_): *δ*(ppm) 170.70, 152.66, 151.02, 133.26, 131.34, 129.94, 126.76, 122.44, 119.84, 119.69, 118.23, 106.45, 63.30, 60.12, 54.04, 50.48, 24.10, 21.89, 21.12, 19.22, 14.45. EIMS, *m*/*z* 542.3 [M]^+^. Elemental analysis for C_21_H_21_Cl_2_F_4_N_3_O_3_S: found C 46.72, H 4.11 N 7.52; calcd C 46.51, H 3.90, N 7.75.

*(1R,2S)-2-(2-(N-(4-Chloro-2-trifluoromethylphenyl)sulfamoyl)-cyclohexylamino)-N-(2-fluoro-4-bromo phenyl)acetamide* **II-7**. White solid, yield: 52.1%. mp 88–90 °C. ^1^H-NMR (600 MHz, DMSO-*d*_6_): *δ*(ppm) 10.03 (s, 1H, NH–C=O), 7.96–7.38 (m, 6H, C_6_H_3_ + C_6_H_3_), 7.12 (s, 2H, NH + NH), 3.68 (d, *J* = 15.3 Hz, 1H, CH–SO_2_), 3.53–3.41 (m, 2H, CH_2_), 3.27 (s, 1H, CH–N), 1.99–1.21 (m, 8H, 4CH_2_). ^13^C-NMR (101 MHz, DMSO-*d*_6_): *δ*(ppm) 169.35, 154.12, 152.46, 133.16, 128.22, 127.90, 126.68, 125.79, 124.59, 124.48, 122.57, 119.29, 119.14, 115.90, 62.88, 54.29, 50.10, 28.01, 24.00, 21.97, 19.32. EIMS, *m*/*z* 588.2 [M]^+^. Elemental analysis for C_21_H_22_BrClF_4_N_3_O_3_S: found C 42.76, H 3.39, N 7.31; calcd C 42.98, H 3.61, N 7.16.

*(1R,2S)-2-(2-(N-(4-Chloro-2-trifluoromethylphenyl)sulfamoyl)-cyclohexylamino)-N-(3-fluoro-4-bromo phenyl)acetamide* **II-8**. White solid, yield: 64.8%. mp 149–151 °C. ^1^H-NMR (600 MHz, DMSO-*d*_6_) :*δ*(ppm) 10.39 (s, 1H, NH–C=O), 7.72–7.31 (m, 6H, C_6_H_3_ + C_6_H_3_), 6.92 (s, 2H, NH + NH), 3.59 (d, *J* = 16.7 Hz, 1H, CH–SO_2_), 3.38 (d, *J* = 23.7 Hz, 2H, CH_2_), 3.32 (s, 1H, CH–N), 1.97–1.29 (m, 8H, 4CH_2_). ^13^C-NMR (101 MHz, DMSO-*d*_6_): *δ*(ppm) 170.02, 159.24, 157.64, 140.03, 133.82, 133.31, 129.16, 126.87, 124.25, 122.43, 116.64, 107.31, 107.13, 101.37, 63.46, 54.13, 50.20, 28.10, 24.12, 21.83, 19.03. EIMS, *m*/*z* 588.1 [M]^+^. Elemental analysis for C_21_H_21_BrClF_4_N_3_O_3_S: found C 42.72, H 3.78, N 6.99; calcd C 42.98, H 3.61, N 7.16.

*(1R,2S)-2-(2-(N-(4-Chloro-2-trifluoromethylphenyl)sulfamoyl)-cyclohexylamino)-N-(2-trifluoromethyl-4-fluorophenyl)acetamide* **II-9**. White solid, yield: 61.4%. mp 153–155 °C. ^1^H-NMR (600 MHz, DMSO-*d*_6_) :*δ*(ppm) 9.86 (s, 1H, NH–C=O), 7.74–7.55 (m, 6H, C_6_H_3_ + C_6_H_3_), 7.01 (s, 2H, NH + NH), 3.61 (d, *J* = 16.4 Hz, 1H, CH–SO_2_), 3.53–3.42 (m, 2H, CH_2_), 3.31 (s, 1H, CH–N), 1.97–1.31 (m, 8H, 4CH_2_). ^13^C-NMR (101 MHz, DMSO-*d*_6_): *δ*(ppm) 170.00, 160.24, 158.62, 133.23, 131.71, 130.72, 128.63, 126.73, 124.30, 123.95, 122.49, 122.14, 120.58, 120.50, 114.20, 63.08, 54.05, 49.82, 28.11, 23.99, 21.93, 19.38. EIMS, *m*/*z* 576.4 [M]^+^. Elemental analysis for C_22_H_21_ClF_7_N_3_O_3_S: found C45.62, H 3.41, N 7.49; calcd C 45.88, H 3.68, N 7.30.

*(1R,2S)-2-(2-(N-(4-Chloro-2-trifluoromethylphenyl)sulfamoyl)-cyclohexylamino)-N-(2-fluoro-5-trifluoromethylphenyl)acetamide* **II-10**. White solid, yield: 65.3%. mp 163–165 °C. ^1^H-NMR (600 MHz, DMSO-*d*_6_): *δ*(ppm) 10.23 (s, 1H, NH–C=O), 8.48–7.51 (m, 8H, NH + NH + C_6_H_3_ + C_6_H_3_), 3.70 (d, *J* = 16.8 Hz, 1H, CH–SO_2_), 3.45 (m, 2H, CH_2_), 3.33 (s, 1H, CH–N), 2.01–1.26 (m, 8H, 4CH_2_). ^13^C-NMR (101 MHz, DMSO-*d*_6_): *δ*(ppm) 170.30, 155.83, 154.16, 133.25, 127.22, 127.14, 126.77, 125.75, 124.27, 123.19, 122.46, 119.65, 117.17, 117.04, 63.24, 54.02, 50.11, 49.35, 28.09, 24.06, 21.89, 19.24. EIMS, *m*/*z* 574.93 [M]^+^. Elemental analysis for C_22_H_21_ClF_3_N_3_O_3_S: found C 45.99, H 3.45, N 7.11; calcd C 45.88, H 3.68, N 7.30.

*(1R,2S)-2-(2-(N-(4-Chloro-2-trifluoromethylphenyl)sulfamoyl)-cyclohexylamino)-N-(3-bromo-4-fluorophenyl)acetamide* **II-11**. White solid, yield: 63.2%. mp 180–182 °C. ^1^H-NMR (600 MHz, DMSO-*d*_6_) :*δ*(ppm) 10.29 (s, 1H, NH–C=O), 8.04–7.33 (m, 8H, NH + NH + C_6_H_3_ + C_6_H_3_), 3.59 (d, *J* = 16.7 Hz, 1H, CH–SO_2_), 3.39 (d, *J* = 29.7 Hz, 2H, CH_2_), 3.33 (d, *J* = 10.8 Hz, 1H, CH–N), 2.01–1.27 (m, 8H, 4CH_2_). ^13^C-NMR (101 MHz, DMSO-*d*_6_): *δ*(ppm) 169.56, 155.43, 153.83, 136.35, 133.30, 126.83, 123.43, 122.45, 120.20, 120.16, 117.27, 117.12, 108.11, 107.97, 63.38, 54.16, 50.04, 28.04, 24.10, 21.84, 19.06. EIMS, *m*/*z* 586.72 [M]^+^. Elemental analysis for C_21_H_21_BrClF_4_N_3_O_3_S: found C 43.18, H 3.88, N 6.98; calcd C 42.98, H 3.61, N 7.16.

*(1R,2S)-2-(2-(N-(4-Chloro-2-trifluoromethylphenyl)sulfamoyl)-cyclohexylamino)-N-(3-cyano-4-fluorophenyl)acetamide* **II-12**. White solid, yield: 57.1%. mp 114–116 °C. ^1^H-NMR (600 MHz, DMSO-*d*_6_): *δ*(ppm) 10.43 (s, 1H, NH–C=O), 8.07–7.52 (m, 6H, C_6_H_3_ + C_6_H_3_), 6.86 (s, 2H, NH + NH), 3.59 (d, *J* = 16.9 Hz, 1H, CH–SO_2_), 3.40 (s, 1H, CH–N), 3.31 (s, 2H, CH_2_), 1.98–1.30 (m, 8H, 4CH_2_). ^13^C-NMR (101 MHz, DMSO-*d*_6_): *δ*(ppm) 169.89, 135.30, 133.57, 133.24, 128.11, 126.69, 126.25, 124.96, 124.31, 123.15, 122.49, 121.34, 63.10, 54.06, 50.05, 28.27, 24.03, 21.89, 19.41. EIMS, *m*/*z* 533.2 [M]^+^. Elemental analysis for C_22_H_21_ClF_4_N_4_O_3_S: found C 49.72 H 3.81, N 10.69; calcd C 49.58, H 3.97, N 10.51.

*(1R,2S)-2-(2-(N-(4-Chloro-2-trifluoromethylphenyl)sulfamoyl)-cyclohexylamino)-N-(2,3,4-trifluorophenyl)acetamide* **II-13**. White solid, yield: 58.2%. mp 159–161 °C. ^1^H-NMR (600 MHz, DMSO-*d*_6_) :*δ*(ppm) 10.09 (s, 1H, NH–C=O), 7.65–7.30 (m, 5H, C_6_H_2_ + C_6_H_3_), 7.05 (s, 2H, NH + NH), 3.67 (d, *J* = 16.4 Hz, 1H, CH–SO_2_), 3.51–3.41 (m, 2H, CH_2_), 3.29–3.23 (m, 1H, CH–N), 1.95–1.25 (m, 8H, 4CH_2_). ^13^C-NMR (101 MHz, DMSO-*d*_6_): *δ*(ppm) 169.56, 148.01, 146.38, 144.13, 142.46, 140.44, 138.79, 133.20, 128.45, 126.75, 124.33, 122.51, 118.38, 112.30, 63.03, 54.21, 49.91, 27.97, 24.01, 21.94, 19.26. EIMS, *m*/*z* 544.3 [M]^+^. Elemental analysis for C_21_H_20_ClF_6_N_3_O_3_S: found C 46.60, H 3.92, N 7.55; calcd C 46.37, H 3.71, N 7.73.

*(1R,2S)-2-(2-(N-(4-Chloro-2-trifluoromethylphenyl)sulfamoyl)-cyclohexylamino)-N-(2,3,4-trifluorophenyl)acetamide* **II-14**. White solid, yield: 67.5%. mp 156–158 °C. ^1^H-NMR (600 MHz, DMSO-*d*_6_) :*δ*(ppm) 10.08 (s, 1H, NH–C=O), 8.05–7.56 (m, 5H, C_6_H_2_ + C_6_H_3_), 6.99 (s, 2H, NH + NH), 3.66 (d, *J* = 16.5 Hz, 1H, CH–SO_2_), 3.50–3.40 (m, 2H, CH_2_), 3.28 (s, 1H, CH–N), 1.95–1.25 (m, 8H, 4CH_2_). ^13^C-NMR (101 MHz, DMSO-*d*_6_): *δ*(ppm) 169.75, 164.12, 137.38, 133.22, 128.62, 126.76, 126.12, 124.30, 122.93, 122.49, 117.37, 111.12, 106.98, 106.32, 54.11, 52.37, 50.03, 28.05, 24.03, 21.92, 19.25. EIMS, *m*/*z* 544.2 [M]^+^. Elemental analysis for C_21_H_20_ClF_6_N_3_O_3_S: found C 46.17, H 3.95, N 7.58; calcd C 46.37, H 3.71, N 7.73.

*(1R,2S)-2-(2-(N-(4-Chloro-2-trifluoromethylphenyl)sulfamoyl)-cyclohexylamino)-N-(2-bromophenyl)acetamide* **II-15**. White solid, yield: 68.7%. mp 173–175 °C. ^1^H-NMR (600 MHz, DMSO-*d*_6_) :*δ*(ppm) 9.91 (s, 1H, NH–C=O), 7.96–7.10 (s, 1H m, 7H, C_6_H_4_ + C_6_H_3_), 7.05–6.79 (m, 2H, NH + NH), 3.54 (s, 2H, CH_2_), 3.46 (s, 1H, CH–SO_2_), 3.30–3.21 (m, 1H, CH–N), 1.97–1.33 (m, 8H, 4CH_2_). ^13^C-NMR (101 MHz, DMSO-*d*_6_): *δ*(ppm) 169.52, 136.06, 133.30, 133.05, 129.11, 128.62, 126.84, 126.80, 126.60, 126.08, 124.26, 122.45, 120.63, 115.56, 63.25, 54.11, 50.46, 28.50, 24.05, 21.91, 19.53. EIMS, *m*/*z* 570.1 [M]^+^. Elemental analysis for C_21_H_22_BrClF_4_N_3_O_3_S: found C 44.55, H 4.13, N 7.19; calcd C 44.34, H 3.90, N 7.39.

*(1R,2S)-2-(2-(N-(4-Chloro-2-trifluoromethylphenyl)sulfamoyl)-cyclohexylamino)-N-(3-bromophenyl)acetamide* **II-16**. White solid, yield: 60.3%. mp 166–168 °C. ^1^H-NMR (600 MHz, DMSO-*d*_6_): *δ*(ppm) 10.25 (s, 1H, NH–C=O), 7.93–7.23 (m, 7H, C_6_H_4_ + C_6_H_3_), 7.00 (s, 2H, NH + NH), 3.59 (d, *J* = 16.2 Hz, 1H, CH–SO_2_), 3.46–3.33 (m, 2H, CH_2_), 3.30–3.19 (m, 1H, CH–N), 2.01–1.29 (m, 8H, 4CH_2_). ^13^C-NMR (101 MHz, DMSO-*d*_6_): *δ*(ppm) 169.60, 140.39, 137.17, 133.28, 131.20, 128.94, 128.29, 126.81, 126.44, 124.29, 122.47, 121.99, 121.63, 118.06, 63.28, 54.23, 50.11, 28.02, 24.06, 21.88, 19.08. EIMS, *m*/*z* 490.2 [M]^+^. Elemental analysis for C_21_H_22_BrClF_3_N_3_O_3_S: found C 44.73, H 4.11, N 7.14; calcd C 44.34, H 3.90, N 7.39.

*(1R,2S)-2-(2-(N-(4-Chloro-2-trifluoromethylphenyl)sulfamoyl)-cyclohexylamino)-N-(2,4-dibromophenyl)acetamide* **II-17**. White solid, yield: 57.8%. mp 178–180 °C. ^1^H-NMR (600 MHz, DMSO-*d*_6_): *δ*(ppm) 9.97 (s, 1H, NH–C=O), 8.02–7.53 (m, 8H, NH + NH + C_6_H_3_ + C6H_3_), 3.54 (m, *J* = 16.7 Hz, 2H, CH_2_), 3.45 (s, 1H, CH–SO_2_), 3.33 (s, 1H, CH–N), 2.06–1.25 (m, 8H, 4CH_2_). ^13^C-NMR (101 MHz, DMSO-*d*_6_): *δ*(ppm) 169.90, 135.66, 134.84, 133.35, 131.57, 130.02, 126.88, 126.85, 125.23, 124.20, 122.39, 117 .12, 116.15, 63.46, 54.00, 50.60, 48.98, 28.66, 24.10, 21.87, 19.48. EIMS, *m*/*z* 648.10 [M]^+^. Elemental analysis for C_21_H_21_Br_2_ClF_3_N_3_O_3_S: found C 39.11, H 3.43, N 6.68; calcd C 38.94, H 3.27, N 6.49.

*(1R,2S)-2-(2-(N-(4-Chloro-2-trifluoromethylphenyl)sulfamoyl)-cyclohexylamino)-N-(2-methylphenyl)acetamide* **II-18**. White solid, yield: 43.8%. mp 184–185 °C. ^1^H-NMR (600 MHz, DMSO-*d*_6_): *δ*(ppm) 9.58 (s, 1H, NH–C=O), 7.61–7.06 (m, 9H, NH + NH + C_6_H_3_ + C_6_H_4_), 3.71 (d, *J* = 16.2 Hz, 1H, CH–SO_2_), 3.52 (d, *J* = 16.0 Hz, 2H, CH_2_), 3.26 (s, 1H, CH–N), 2.21 (s, 3H, CH_3_), 2.05–1.30 (m, 8H, 4CH_2_). ^13^C-NMR (101 MHz, DMSO-*d*_6_): *δ*(ppm) 168.12, 136.10, 133.07, 131.16, 130.91, 130.73, 127.41, 126.64, 126.60, 126.42, 125.36, 124.52, 124.02, 122.71, 62.44, 54.62, 49.84, 27.74, 23.89, 22.07, 19.36, 18.00. EIMS, *m*/*z* 504.3 [M]^+^. Elemental analysis for C_22_H_25_ClF_3_N_3_O_3_S: found C 52.26, H 5.21, N 8.16; calcd C 52.43, H5.00, N 8.34.

*(1R,2S)-2-(2-(N-(4-Chloro-2-trifluoromethylphenyl)sulfamoyl)-cyclohexylamino)-N-(2-trifluoromethylphenyl)acetamide* **II-19**. White solid, yield: 40.3%. mp 151–152 °C. ^1^H-NMR (600 MHz, DMSO-*d*_6_) :*δ*(ppm) 9.89 (s, 1H, NH–C=O), 7.79–7.41 (m, 6H, C_6_H_3_ + C_6_H_3_), 6.99 (s, 2H, NH + NH), 3.59 (s, 1H, CH–SO_2_), 3.52 (m, 2H, CH_2_), 3.44 (s, 1H, CH–N), 1.96–1.32 (m, 8H, 4CH_2_). ^13^C-NMR (101 MHz, DMSO-*d*_6_): *δ*(ppm) 169.89, 135.30, 133.57, 133.24, 128.11, 126.69, 126.25, 124.96, 124.31, 123.15, 122.49, 121.34, 63.10, 54.06, 50.05, 28.27, 24.03, 21.89, 19.41. EIMS, *m*/*z* 558.3 [M]^+^. Elemental analysis for C_22_H_22_ClF_6_N_3_O_3_S: found C 47.21, H 4.11, N 7.72; calcd C 47.36, H 3.97, N 7.53.

*(1R,2S)-2-(2-(N-(4-Chloro-2-trifluoromethylphenyl)sulfamoyl)-cyclohexylamino)-N-(3-trifluoromethylphenyl)acetamide* **II-20**. White solid, yield: 71.5%. mp 175–177 °C. ^1^H-NMR (600 MHz, DMSO-*d*_6_) :*δ*(ppm) 10.41 (s, 1H, NH–C=O), 8.07–7.41 (m, 7H, C_6_H_4_ + C_6_H_3_), 6.94 (s, 2H, NH + NH), 3.61 (d, *J* = 16.2 Hz, 1H, CH–SO_2_), 3.39 (d, *J* = 21.9 Hz, 2H, CH_2_), 3.32 (s, 1H, CH–N), 1.99–1.30 (m, 8H, 4CH_2_). ^13^C-NMR (101 MHz, DMSO-*d*_6_): *δ*(ppm) 170.08, 164.71, 139.57, 133.28, 130.44, 129.83, 126.82, 125.33, 124.23, 123.53, 122.80, 122.41, 120.15, 115.36, 63.50, 54.10, 52.73, 50.10, 28.04, 24.12, 21.83, 19.04. EIMS, *m*/*z* 558.2 [M]^+^. Elemental analysis for C_22_H_22_ClF_6_N_3_O_3_S: found C 47.58, H 3.75, N 7.68; calcd C 47.36, H 3.97, N 7.53.

*(1R,2S)-2-(2-(N-(4-Chloro-2-trifluoromethylphenyl)sulfamoyl)-cyclohexylamino)-N-(3,5-ditrifluoromethylphenyl)acetamide* **II-21**. White solid, yield: 53.0%. mp 213–215 °C. ^1^H-NMR (600 MHz, DMSO-*d*_6_): *δ*(ppm) 10.69 (s, 1H, NH–C=O), 8.28–7.58 (m, 6H, C_6_H_3_ + C_6_H_3_), 6.65 (s, 2H, NH + NH), 3.59 (d, *J* = 17.0 Hz, 1H, CH–SO_2_), 3.37 (d, *J* = 14.8 Hz, 2H, CH_2_), 3.31 (s, 1H, CH–N), 2.03–1.31 (m, 8H, 4CH_2_). ^13^C-NMR (101 MHz, DMSO-*d*_6_): *δ*(ppm) 171.48, 140.66, 133.37, 131.35, 131.13, 130.92, 130.24, 126.94, 126.25, 124.45, 124.03, 122.64, 122.22, 120.83, 118.89, 116.57, 64.18, 53.74, 50.21, 28.24, 24.29, 21.66, 18.84. EIMS, *m*/*z* 626.3 [M]^+^. Elemental analysis for C_23_H_21_ClF_9_N_3_O_3_S: found C 44.39, H 3.19, N 6.57; calcd C 44.13, H 3.38, N 6.71.

*(1R,2S)-2-(2-(N-(4-Chloro-2-trifluoromethylphenyl)sulfamoyl)-cyclohexylamino)-N-(3-methoxyphenyl)acetamide* **II-22**. White solid, yield: 62.4%. mp 185–187 °C. ^1^H-NMR (600 MHz, DMSO-*d*_6_) :*δ*(ppm) 10.12 (s, 1H, NH–C=O), 7.60–6.64 (m, 9H, NH + NH + C_6_H_3_ + C_6_H_4_), 3.71 (s, 3H, CH_3_), 3.62 (d, *J* = 15.3 Hz, 1H, CH–SO_2_), 3.48–3.35 (m, 2H, CH_2_), 3.30–3.21 (m, 1H, CH–N), 1.97–1.29 (m, 8H, 4CH_2_). ^13^C-NMR (101 MHz, DMSO-*d*_6_): *δ*(ppm) 168.68, 159.92, 139.96, 133.17, 130.01, 128.25, 127.35, 126.73, 126.23, 124.42, 122.60, 111.63, 109.30, 105.17, 62.82, 55.30, 54.47, 53.69, 50.00, 27.83, 23.97, 21.97, 19.19. EIMS, *m*/*z* 520.2 [M]^+^. Elemental analysis for C_22_H_25_ClF_3_N_3_O_4_S: found C 51.03, H 5.09, N 7.84; calcd C 50.82, H 4.85, N 8.08.

*(1R,2S)-2-(2-(N-(4-Chloro-2-trifluoromethylphenyl)sulfamoyl)-cyclohexylamino)-N-(4-methoxyphenyl)acetamide* **II-23**. White solid, yield: 59.1%. mp 158–160 °C. ^1^H-NMR (600 MHz, DMSO-*d*_6_): *δ*(ppm) 10.02 (s, 1H, NH–C=O), 7.60–6.88 (m, 9H, NH + NH + C_6_H_3_ + C_6_H_4_), 3.71 (s, 3H, CH_3_), 3.64 (d, *J* = 16.6 Hz, 1H, CH–SO_2_), 3.51–3.37 (m, 2H, CH_2_), 3.26 (s, 1H, CH–N), 2.02–1.29 (m, 8H, 4CH_2_). ^13^C-NMR (101 MHz, DMSO-*d*_6_): *δ*(ppm) 167.59, 155.76, 133.07, 131.97, 127.64, 126.65, 126.61, 126.35, 124.53, 122.72, 120.84 (2), 114.32 (2), 62.42, 55.54, 54.66, 49.74, 27.67, 23.87, 22.04, 19.29. EIMS, *m*/*z* 520.1 [M]^+^. Elemental analysis for C_21_H_25_ClF_3_N_3_O_4_S: found C 51.01, H 4.65, N 7.90; calcd C 50.82, H 4.85, N 8.08.

*(1R,2S)-2-(2-(N-(4-Chloro-2-trifluoromethylphenyl)sulfamoyl)-cyclohexylamino)-N-(2-trifluoromethoxyphenyl)acetamide* **II-24**. White solid, yield: 75.3%. mp 147–148 °C. ^1^H-NMR (600 MHz, DMSO-*d*_6_): *δ*(ppm) 9.96 (s, 1H, NH–C=O), 8.07–7.24 (m, 7H, C_6_H_4_ + C_6_H_3_), 7.12 (s, 2H, NH + NH), 3.65 (s, 1H, CH–SO_2_), 3.57–3.45 (s, 2H, CH_2_), 3.29–3.21 (m, 1H, CH–N), 1.96–1.26 (m, 8H, 4CH_2_). ^13^C-NMR (101 MHz, DMSO-*d*_6_): *δ*(ppm) 169.28, 139.40, 133.18, 130.62, 128.08, 126.73, 125.59, 124.35, 123.82, 123.08, 122.54, 121.50, 121.37, 119.67, 117.96, 62.90, 54.19, 50.28, 28.25, 23.99, 21.87, 19.43. EIMS, *m*/*z* 574.3 [M]^+^. Elemental analysis for C_22_H_22_ClF_6_N_3_O_4_S: found C 45.91, H 4.02, N 7.51; calcd C 46.04, H 3.86, N 7.32.

*(1R,2S)-2-(2-(N-(4-Chloro-2-trifluoromethylphenyl)sulfamoyl)-cyclohexylamino)-N-(2-chlorobenzyl)acetamide* **II-25**. White solid, yield: 50.8%. mp 130–131 °C. ^1^H-NMR (600 MHz, DMSO-*d*_6_): *δ*(ppm) 8.70 (s, 1H, NH–C=O), 7.77 (s, 2H, NH + NH), 7.54–7.30 (m, 7H, C_6_H_4_ + C_6_H_3_), 4.38 (qd, *J* = 15.6, 5.9 Hz, 2H,CH_2_), 3.67 (d, *J* = 15.8 Hz, 1H, CH–SO_2_), 3.51 (d, *J* = 14.7 Hz, 2H, CH_2_), 3.25 (s, 1H, CH–N), 2.00–1.27 (m, 8H, 4CH_2_). ^13^C-NMR (101 MHz, DMSO-*d*_6_): *δ*(ppm) 168.59, 136.09, 132.82, 132.55, 129.91, 129.47 (2), 129.23, 127.82, 127.51, 126.39, 124.76, 122.95, 61.46, 54.94, 48.77, 27.22, 23.62, 22.18, 19.57. EIMS, *m*/*z* 538.4 [M]^+^. Elemental analysis for C_22_H_24_Cl_2_F_3_N_3_O_3_S: found C 48.98, H 4.58, N 7.97; calcd C 49.08, H4.49, N 7.80.

*(1R,2S)-2-(2-(N-(4-Chloro-2-trifluoromethylphenyl)sulfamoyl)-cyclohexylamino)-N-(2-bromobenzyl)acetamide* **II-26**. White solid, yield: 54.6%. mp 123–124 °C. ^1^H-NMR (600 MHz, DMSO-*d*_6_) :*δ*(ppm) 8.70 (m, *J* = 5.7 Hz, 1H, NH–C=O), 7.88–7.20 (m, 9H, NH + NH + C_6_H_3_ + C_6_H_4_), 4.35 (m, *J* = 5.9 Hz, 2H, CH_2_–Ph), 3.68 (d, *J* = 16.1 Hz, 1H, CH–SO_2_), 3.51 (m, 2H, CH_2_), 3.26 (s, 1H,CH–N), 2.04–1.26 (m, 8H, 4CH_2_). ^13^C-NMR (101 MHz, DMSO-*d*_6_): *δ*(ppm) 168.62, 137.60, 132.86, 132.79, 130.03, 129.50, 129.49, 128.08, 126.46, 126.42, 124.73, 121.11, 118.99, 61.46, 54.95, 48.98, 48.75, 43.10, 27.19, 23.59, 22.19, 19.59. EIMS, *m*/*z* 582.0 [M]^+^. Elemental analysis for C_22_H_24_BrClF_3_N_3_O_3_S: found C 45.21, H 3.99, N 7.36; calcd C 45.34, H 4.15, N 7.21.

*(1R,2S)-2-(2-(N-(4-Chloro-2-trifluoromethylphenyl)sulfamoyl)-cyclohexylamino)-N-(2-methylbenzyl)acetamide* **II-27**. White solid, yield: 52.1%. mp 135–137 °C. ^1^H-NMR (600 MHz, DMSO-*d*_6_) :*δ*(ppm) 8.54 (s, 1H, NH–C=O), 7.89 (s, 2H, NH + NH), 7.50–7.19 (m, 7H, C_6_H_4_ + C_6_H_3_), 4.54 (s, 3H,CH_3_), 4.28 (m, 2H, CH_2_), 3.65 (d, *J* = 15.4 Hz, 1H, CH–SO_2_), 3.49 (m, 2H, CH_2_), 3.22 (m, 1H, CH–N), 2.03–1.30 (m, 8H, 4CH_2_). ^13^C-NMR (101 MHz, DMSO-*d*_6_): *δ*(ppm) 167.86, 164.25, 136.66 (2), 136.14, 133.99, 132.74, 130.75, 130.34, 128.28, 127.87, 127.48, 126.32, 126.12, 61.15, 55.11, 49.45, 48.65, 46.48, 27.10, 22.22, 19.05. EIMS, *m*/*z* 518.3 [M]^+^. Elemental analysis for C_23_H_27_ClF_3_N_3_O_3_S: found C 53.46, H 5.01, N 8.39; calcd C 53.33, H 5.25, N 8.11.

*(1R,2S)-2-(2-(N-(4-Chloro-2-trifluoromethylphenyl)sulfamoyl)-cyclohexylamino)-N-(2-bromophenethyl)acetamide* **II-28**. White solid, yield: 45.2%. mp 113–115 °C. ^1^H-NMR (600 MHz, DMSO-*d_6_*): *δ*(ppm) ^1^H-NMR (600 MHz, DMSO) δ 8.33 (m, *J* = 5.2 Hz, 1H, NH–C=O), 7.94–7.11 (m, 9H, NH + NH + C_6_H_3_ + C_6_H_4_), 3.54 (d, *J* = 15.9 Hz, 1H, CH–SO_2_), 3.42 (d, *J* = 33.4 Hz, 2H, CH_2_), 3.35 (m, 2H), 3.22 (s, 1H, CH–N), 2.84 (t, *J* = 7.3 Hz, 2H), 2.01–1.26 (m, 8H, 4CH_2_). ^13^C-NMR (101 MHz, DMSO-*d*_6_): *δ*(ppm) 168.11, 138.52, 132.92, 132.82, 132.41, 131.37, 128.91, 128.22, 126.38, 125.96, 124.24, 122.98, 113.15, 61.24, 54.96, 48.72, 38.93, 35.50, 27.07, 27.05, 23.50, 22.23, 19.54. EIMS, *m*/*z* 597.0 [M]^+^. Elemental analysis for C_23_H_26_BrClF_3_N_3_O_4_S: found C 46.54, H 4.11, N 6.89; calcd C 46.28, H 4.39, N 7.04.

#### 3.2.4. Synthesis of *N*-(2-Trifluoromethyl-4-chlorophenyl)-2-ethoxyacylmethyl Amino Cyclohexyl Sulfonamide **L-4** and *N*-(2-Trifluoromethyl-4-chlorophenyl)-2-carboxymethyl Amino Cyclohexyl Sulfonamide **L-5**

**L-4** was synthesized according to the method in Section 3.2.3. Subsequently, **L-4** was dissolved in CH_3_OH and transferred to a round bottom flask containing 1 mol L^−1^ NaOH solution and stirred for 3 h. CH_3_OH was evaporated under vacuum on rotavapor, then 3 mol L^−1^ HCl solution was added to adjust to pH 3, and the formed precipitate was collected by filtration to obtain **L-5** as white solid.

#### 3.2.5. Synthesis of 2-Glycinamide Cyclohexyl Sulfonamide Derivatives **II-29** to **II-33**

Under nitrogen atmosphere, **L-5** (1.5 mmol), HOBt (1.8 mmol), EDCI (1.8 mmol), and Et_3_N (1.8 mmol) were placed in a three-necked flask with 30 mL CH_2_Cl_2_, and stirred for 3 h at 0 °C. Then, amine was added slowly and allowed to react for 8 h. The reaction was monitored by TLC and after completion of reaction, the solution was washed with saturated aq NaHCO_3_ solution and distilled water successively. Then it was dried over anhydrous Na_2_SO_4_, filtered and evaporated on rotavapor in vacuum. Finally, products were purified by silica gel column chromatography and recrystallized to obtain pure products **II-29** to **II-33**.

*(1R,2S)-2-(2-(N-(4-Chloro-2-trifluoromethylphenyl)sulfamoyl)-cyclohexylamino)-N-ethylacetamide* **II-29**. White solid, yield: 68.2%. mp 128–129 °C. ^1^H-NMR (600 MHz, DMSO-*d*_6_): *δ*(ppm) 8.18 (t, *J* = 5.1 Hz, 1H, NH–C=O), 7.90 (s, 1H, NH–Ph), 7.49 (m, 3H, C_6_H_3_), 3.57 (d, *J* = 15.8 Hz, 1H, CH–SO_2_), 3.50 (s, 2H, CH_2_–C=O), 3.39 (d, *J* = 15.8 Hz, 1H, NH), 3.21 (d, *J* = 9.8 Hz, 1H, CH–N), 3.11 (m, 2H, CH_2_), 2.04–1.27(m, 8H, 4CH_2_), 1.02 (t, *J* = 7.2 Hz, 3H, CH_3_). ^13^C-NMR (101 MHz, DMSO-*d*_6_): *δ*(ppm) 167.43, 132.75, 130.03, 126.69, 126.33, 124.82, 123.13, 61.05, 55.08, 48.61, 33.87, 27.00, 26.75, 23.54, 22.19, 19.56, 14.78. EIMS, *m*/*z* 442.1 [M]^+^. Elemental analysis for C_17_H_23_ClF_3_N_3_O_3_S: found C 46.17, H 5.04, N 9.76; calcd C 46.21, H 5.25, N 9.51.

*(1R,2S)-2-(2-(N-(4-Chloro-2-trifluoromethylphenyl)sulfamoyl)-cyclohexylamino)-N-propylacetamide* **II-30**. White solid, yield: 73.2%. mp 139–141 °C. ^1^H-NMR (600 MHz, DMSO-*d*_6_): *δ*(ppm) 8.19 (t, *J* = 5.5 Hz, 1H, NH–C=O), 7.94 (s, 1H, NH–Ph), 7.48 (m, 3H, C_6_H_3_), 3.59 (d, *J* = 15.9 Hz, 1H, CH–SO_2_), 3.51 (d, *J* = 3.0 Hz, 2H, CH_2_–C=O), 3.42 (d, *J* = 15.9 Hz, 1H, NH), 3.21 (d, *J* = 10.4 Hz, 1H, CH–N), 3.05 (m, 2H, CH_2_), 2.04–1.26 (m, 10H, 4CH_2_ + CH_2_), 0.84 (t, *J* = 7.4 Hz, 3H, CH_3_). ^13^C-NMR (101 MHz, DMSO-*d*_6_): *δ*(ppm) 167.57, 142.07, 132.73, 126.38, 125.63, 124.79, 123.06, 121.21, 61.03, 55.11, 48.59, 40.76, 26.99, 23.54, 22.62, 22.19, 19.55, 11.68. EIMS, *m*/*z* 456.0 [M]^+^. Elemental analysis for C_18_H_25_ClF_3_N_3_O_3_S: found C 47.16, H 5.35, N 9.47; calcd C 47.42, H 5.53, N 9.22.

*(1R,2S)-2-(2-(N-(4-Chloro-2-trifluoromethylphenyl)sulfamoyl)-cyclohexylamino)-N-cyclopropylacetamide* **II-31**. White solid, yield: 65.7%. mp 99–101 °C. ^1^H-NMR (600 MHz, DMSO-*d*_6_): *δ*(ppm) 8.23 (d, *J* = 3.9 Hz, 1H, NH–C=O), 7.72 (m, 1H, NH–Ph), 7.50 (m, 3H,C_6_H_3_), 3.52 (d, *J* = 15.9 Hz, 1H, CH–SO_2_), 3.47 (d, *J* = 3.0 Hz, 2H, CH_2_–C=O), 3.35 (s, 1H, NH), 3.20 (d, *J* = 10.6 Hz, 1H, CH–N), 2.65 (m, 1H, CH–N–C=O), 1.99–1.27 (m, 8H, 4CH_2_), 0.44–0.66 (m, 4H, CH_2_ + CH_2_). ^13^C-NMR (101 MHz, DMSO-*d*_6_): *δ*(ppm) 169.13, 132.84, 126.43, 124.72, 122.99, 61.26, 60.15, 54.99, 48.65, 27.12, 23.58, 22.55, 22.17, 21.13, 19.51, 14.46, 5.95, 5.81. EIMS, *m*/*z* 454.1 [M]^+^. Elemental analysis for C_18_H_23_ClF_3_N_3_O_3_S: found C 47.87, H 4.96, N 9.54; calcd C 47.63, H 5.11, N 9.26.

*(1R,2S)-2-(2-(N-(4-Chloro-2-trifluoromethylphenyl)sulfamoyl)-cyclohexylamino)-N-cyclopropylmethylacetamide* **II-32**. White solid, yield: 66.5%. mp 147–149 °C. ^1^H-NMR (600 MHz, DMSO-*d*_6_): *δ*(ppm) 8.29 (m, *J* = 5.2 Hz, 1H, NH–C=O), 7.92 (s, 1H, NH–Ph), 7.49 (dd, *J* = 8.7 Hz, 3H, C_6_H_3_), 3.60 (d, *J* = 15.8 Hz, 1H, CH–SO_2_), 3.51 (s, 2H, CH_2_–C=O), 3.42 (d, *J* = 15.9 Hz, 1H, NH), 3.21 (d, *J* = 9.3 Hz, 1H, CH–N), 2.98 (m, 2H, CH_2_), 2.05–1.26 (m, 8H, 4CH_2_), 0.12–0.09 (m, 5H, C_3_H_5_). ^13^C-NMR (101 MHz, DMSO-*d*_6_): *δ*(ppm) 167.54, 132.85, 126.34, 126.31, 124.94, 124.87, 123.06, 60.98, 55.14, 48.64, 43.44, 26.99, 26.93, 23.55, 22.20, 21.11, 19.74, 11.09, 3.50. EIMS, *m*/*z* 468.1 [M]^+^. Elemental analysis for C_19_H_25_ClF_3_N_3_O_3_S: found C 48.98, H 5.28, N 9.12; calcd C 48.77, H 5.39, N 8.98.

*(1R,2S)-2-(2-(N-(4-Chloro-2-trifluoromethylphenyl)sulfamoyl)-cyclohexylamino)-N-butylacetamide* **II-33**. White solid, yield: 68.9%. mp 122–124 °C. ^1^H-NMR (600 MHz, DMSO-*d*_6_): *δ*(ppm) 8.17 (t, *J* = 5.4 Hz, 1H, NH–C=O), 7.93 (s, 1H, NH–Ph), 7.49 (m, 3H,C_6_H_3_), 3.58 (d, *J* = 15.8 Hz, 1H, CH–SO_2_), 3.50 (d, *J* = 2.7 Hz, 2H, CH_2_–C=O), 3.41 (d, *J* = 15.9 Hz, 1H, NH), 3.21 (d, *J* = 10.4 Hz, 1H, CH–N), 3.09 (m, 2H, CH_2_–N–C=O), 2.04–1.22 (m, 12H, 4CH_2_ + CH_2_ + CH_2_), 0.85 (t, *J* = 7.3 Hz, 3H, CH_3_). ^13^C-NMR (101 MHz, DMSO-*d*_6_): *δ*(ppm) 167.61, 132.66, 130.05, 126.34, 125.65, 124.86, 123.05, 121.22, 61.05, 55.08, 48.58, 38.61, 31.41, 26.99, 23.54, 22.26, 19.81, 19.55, 13.89. EIMS, *m*/*z* 469.6 [M]^+^. Elemental analysis for C_19_H_27_ClF_3_N_3_O_3_S: found C 48.22, H 5.99, N 9.12; calcd C 48.56, H 5.79, N 8.94.

### 3.3. Fungal Bioassay

In vitro and in vivo fungicidal activities of all target compounds against *B. cinerea* were tested by mycelium growth inhibition tests and greenhouse pot experiments. Three strains of *B. cinerea* (CY-09, DL-11, HLD-15) were isolated from naturally infected tomato leaves in three different areas (CY-09, DL-11, and HLD-15 came from Chaoyang, Dalian, and Huludao, respectively) in Liaoning Province, China. Carbendazim, procymidone, boscalid, pyrimethamine, and cyprodinil were provided by Shenyang Research Institute of the Chemical Industry, National Pesticides Engineering Research Centre and used as positive control.

#### 3.3.1. Evaluation of Target Compounds **II** on the Inhibition of *B. cinerea*

Preliminary tests verified the in vitro fungicidal effect of *B. cinerea* on plates by the mycelium growth inhibition method. Commercial fungicides were used as positive control, while acetone was the blank. Compounds to be tested were dissolved in acetone and diluted with sterilized molten potato-dextrose agar (PDA) medium to obtain the drug-containing medium at the final concentration of 50 μg mL^−1^, and poured it into sterile 90 mm Petri dishes (15 mL per dish). The medium solidified and reduced to room temperature, and 5 mm plugs of *B. cinerea* culture were placed in the center of the PDA plates. Then, the plates were sealed with parafilm and incubated under a regular 12:12 h light/dark regimen at 26 °C for 96 h. The radial growth diameters were measured, and relative inhibition rate of treatment compared to blank assay was calculated by the following equation:

Relative inhibition rate (%) = (d_c_ − d_t_/d_c_ − d_d_) × 100%

where d_d_ was the diameter of the original disk (5 mm), d_c_ and d_t_ represented the average diameter of 3 replicates of the control and treatment plates, respectively.

#### 3.3.2. Evaluation of the Fungicidal Activity on *B. cinerea* by Concentration Gradient Test

Based on the result of multiple preliminary tests, the EC_50_ values of specific compounds were evaluated to verify further fungicidal effect on *B. cinerea* by the method as previously described. The four concentrations of the tested compounds in PDA were 50, 12.5, 3.125 and 0.78125 μg mL^−1^. The EC_50_ values were calculated using log-probit analysis.

#### 3.3.3. In Vivo Fungicidal Activity against *B. cinerea* (CY-09) by Greenhouse Pot Experiments

##### (1). Evaluation of Fungicidal Activity on Cucumber Leaves

The compounds to be tested were processed to 5% emulsifiable concentrate (EC) formulations and diluted with water to 500 μg mL^−1^. The rest of the procedures were given in ref [17]. The seedlings were maintained in a greenhouse pot at 24 ± 1 °C and relative humidity above 90%. When the blank control was infected completely, the lesion diameters of all cotyledons were measured. The calculation method was referred to the formula given in Section 3.3.1.

##### (2). Evaluation of Fungicidal Activity on Tomato Leaves

The conidia of *B. cinerea* were obtained from a 4-week-old PDA medium, which was cultured at 25 °C. The mixtures of 10 mL sterile distilled water and 0.05 mL Tween 80 were poured into culture dishes to let the spores fall off. The spore-rich suspension was filtered through four layers of sterilized gauze to remove mycelium. Finally, the conidia concentration was measured using a hemocytometer and diluted to 10^6^–10^7^ spores per mL. The processing method of the compound-containing EC formulation at the concentration of 500 μg mL^−1^ and 200 μg mL^−1^ was identical to Section 3.3.3 (1). When the number of tomato leaves was about 30, the solution of the compound to be tested were sprayed evenly onto tomato leaves and dried naturally. Subsequently, the spore suspension was sprayed on seedlings and plants kept in the same condition as test Section 3.3.3 (1). When the blank assay was infected completely, the Disease Index of all leaves were investigated and the Control Efficiency was calculated [37].

## 4. Conclusions

In summary, 33 novel 2-glycinamide cyclohexyl sulfonamide derivatives were designed and synthesized. **II-4**, **II-5**, and **II-15** were found as the best active compounds in inhibiting *B. cinerea* in this series of compounds, which were superior to the commercial fungicides carbendazim, procymidone, boscalid, pyrimethanil, and cyprodinil in both in vitro and in vivo tests. The structure–activity relationship showed that docking glycinamide on the lead compound and introducing a benzene ring structure with two fluorine atoms at 2,4- or 2,5-positions could greatly enhance activity. Studies on the structural optimization and mechanism of action are in progress. We believe these compounds will become promising candidates for pesticides.

## Figures and Tables

**Table 1:** In vitro fungicidal activities of target compounds against *Botrytis cinerea* (CY-09).

Compd.	R	Inhibition Rate (%)	EC_50_ Values
**II-1**	C_6_H_5_–	12.49	>100
**II-2**	3-F–C_6_H_4_–	51.08	>100
**II-3**	4-F–C_6_H_4_–	76.89	16.23
**II-4**	2,4-2F–C_6_H_3_–	90.66	4.01
**II-5**	2,5-2F–C_6_H_3_–	85.74	3.38
**II-6**	2-F–4-Cl–C_6_H_3_–	89.18	3.38
**II-7**	2-F–4-Br–C_6_H_3_–	81.32	9.44
**II-8**	3-F–4-Br–C_6_H_3_–	84.76	7.23
**II-9**	2-CF_3_–4-F–C_6_H_3_–	84.51	23.57
**II-10**	2-F–5-CF_3_–C_6_H_3_–	69.27	13.66
**II-11**	3-Br–4F–C_6_H_3_–	72.71	12.68
**II-12**	3-CN–4-F–C_6_H_3_–	90.41	16.27
**II-13**	2,3,4-3F–C_6_H_2_–	90.90	14.67
**II-14**	2,4,5-3F–C_6_H_2_–	88.94	8.9
**II-15**	2-Br–C_6_H_4_–	85.25	4.99
**II-16**	3-Br–C_6_H_4_–	53.05	>100
**II-17**	2,4-2Br–C_6_H_3_–	73.45	16.48
**II-18**	2-CH_3_–C_6_H_4_–	33.87	>100
**II-19**	2-CF_3_–C_6_H_4_–	82.79	3.26
**II-20**	3-CF_3_–C_6_H_4_–	6.59	>100
**II-21**	3,5-2CF_3_–C_6_H_3_–	31.91	>100
**II-22**	3-CH_3_O–C_6_H_4_–	14.70	>100
**II-23**	4-CH_3_O–C_6_H_4_–	33.38	>100
**II-24**	2-CF_3_O–C_6_H_4_–	51.57	>100
**II-25**	2-Cl-C_6_H_4_-CH_2_–	56.00	8.02
**II-26**	2-Br–C_6_H_4_–CH_2_–	88.20	3.4
**II-27**	2-CH_3_–C_6_H_4_–CH_2_–	66.81	15.13
**II-28**	2-Br–C_6_H_4_–CH_2_CH_2_–	75.42	3.57
**II-29**	CH_3_CH_2_–	55.75	34.18
**II-30**	CH_3_(CH_2_)_2_–	75.42	5.9
**II-31**	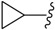	69.52	3.77
**II-32**	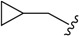	81.32	14.07
**II-33**	CH_3_(CH_2_)_3_–	83.78	6.5
Carbendazim	/	33.87	867.82
Procymidone	/	69.27	10.31
Boscalid	/	89.18	4.46

**Table 2:** In vitro fungicidal activities of specific target compounds against *B. cinerea* (HLD-15 and DL-11).

Compd.	R	HLD-15		DL-11	
		Inhibition Rate (%)	EC_50_ Values	Inhibition Rate (%)	EC_50_ Values
**II-4**	2,4-2F–C_6_H_3_–	83.41	1.88	89.47	2.07
**II-5**	2,5-2F–C_6_H_3_–	83.93	2.3	87.63	2.55
**II-6**	2-F–4-Cl–C_6_H_3_–	71.75	2.13	91.57	3.94
**II-26**	2-Br–C_6_H_4_–CH_2_–	70.19	2.96	94.47	2.92
**II-27**	2-CH_3_–C_6_H_4_–CH_2_–	79.52	7.25	78.94	5.55
**II-28**	2-Br–C_6_H_4_–CH_2_CH_2_–	96.89	2.26	95.00	2.47
**II-30**	CH_3_(CH_2_)_2_–	84.19	7.12	86.05	5.79
**II-31**	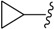	75.12	5.31	65.24	4.36
**II-33**	CH_3_(CH_2_)_3_–	76.67	6.34	95.26	2.97
Carbendazim	/	10.32	427.78	10.48	673.38
Procymidone	/	79.00	10.13	75.25	8.61
Boscalid	/	87.82	2.44	86.57	2.27

**Table 3:** Fungicidal activities of specific compounds in cucumber pot test at 500 μg mL^−1^.

Compd.	Inhibition Rate (%)	Compd.	Inhibition Rate (%)
**II-2**	33.33	**II-17**	59.52
**II-3**	79.76	**II-18**	20.83
**II-4**	90.48	**II-20**	64.88
**II-5**	93.45	**II-23**	27.38
**II-6**	86.31	**II-24**	71.43
**II-7**	80.36	**II-25**	35.71
**II-8**	79.17	**II-26**	53.57
**II-9**	73.81	**II-27**	37.50
**II-10**	72.62	**II-28**	51.19
**II-11**	62.50	**II-29**	66.07
**II-12**	92.86	**II-30**	42.86
**II-13**	91.07	**II-31**	63.09
**II-14**	75.60	**II-32**	71.43
**II-15**	88.69	**II-33**	75.00
**II-16**	30.95	Pyrimethanil	82.14
Carbendazim	59.52	Boscalid	88.10
Procymidone	83.33	Cyprodinil	88.69

**Table 4:** Control efficiency of specific compounds against *B. cinerea* in tomato pot tests.

Compd.	500 μg mL^−1^		200 μg mL^−1^	
	Disease Index	Control Efficacy (%)	Disease Index	Control Efficacy (%)
**II-2**	5.82	74.43	11.37	70.98
**II-4**	7.47	67.19	8.42	78.51
**II-5**	7.19	68.43	8.26	78.91
**II-6**	18.66	18.04	/	/
**II-7**	5.96	73.83	12.10	69.11
**II-8**	4.52	80.14	4.71	87.98
**II-10**	12.69	44.27	/	/
**II-11**	11.28	50.46	/	/
**II-13**	21.39	6.04	/	/
**II-14**	9.73	57.27	/	/
**II-15**	5.25	76.96	4.71	87.97
**II-16**	19.71	13.43	/	/
**II-17**	14.52	36.25	/	/
**II-18**	14.72	35.33	/	/
**II-19**	17.21	24.40	/	/
**II-20**	21.71	4.66	/	/
**II-24**	20.99	7.81	/	/
**II-26**	13.30	41.57	/	/
**II-28**	8.21	63.93	12.61	67.83
**II-29**	9.17	59.74	/	/
**II-30**	11.56	49.21	/	/
**II-31**	8.58	62.33	15.08	61.51
**II-32**	16.02	29.63	/	/
**II-33**	6.37	72.02	9.74	75.15
Pyrimethanil	15.25	33.00	19.62	49.93
Procymidone	5.91	74.05	13.45	65.67
Boscalid	5.23	77.05	8.58	78.10
CK	22.77	/	39.18	/

## Supplementary Materials

Supplementary materials are available online. The ^1^H-NMR and ^13^C-NMR spectra of target compounds **II** and detailed description of the crystal structure of **II-19**.
