# Supplementary material for: Design, Synthesis, and SAR of Novel 2-Glycinamide Cyclohexyl Sulfonamide Derivatives against Botrytis cinerea
Source: Molecules. 2018 Mar 23;23(4):740. doi: 10.3390/molecules23040740 (PMC6017058; doi:10.3390/molecules23040740)
Supplement: Supplementary file 1 [file molecules-23-00740-s001.pdf]

Design, synthesis and SAR of novel 2-glycinamide cyclohexyl sulfonamide derivatives against *Botrytis cinerea*

Nan Cai [1], Caixiu Liu [1], Zhihui Feng [1], Xinghai Li [1,*], Zhiqiu Qi [1], Mingshan Ji [1], Peiwen Qin [1], Wasim Ahmed [2] and Zining Cui [2,*]

[1] Department of Pesticide Science, Plant Protection College, Shenyang Agricultural University, Shenyang 110866, Liaoning, China

[2] State Key Laboratory for Conservation and Utilization of Subtropical Agro-bioresources, Integrative Microbiology Research Centre, Guangdong Province Key Laboratory of Microbial Signals and Disease Control, South China Agricultural University, Guangzhou 510642, Guangdong, China

Page 2-34: ^{1}H NMR and ^{13}C NMR spectra of target compounds II-1 to II-33

Page 35-42: Detailed description of the crystal structures of II-19

To whom Correspondence should be addressed. Tel.: +86-24-8834-2018(X. H. Li), +86-20-8528-8229 (Z. N. Cui); fax: +86-24-8848-7148(X. H. Li), +86-20-8528-8229 (Z. N. Cui). E-mail address: xinghai30@163.com (X. H. Li), ziningcui@scau.edu.cn (Z. N. Cui).

^{1}H NMR and ^{13}C NMR spectra and of target compounds II1-II33

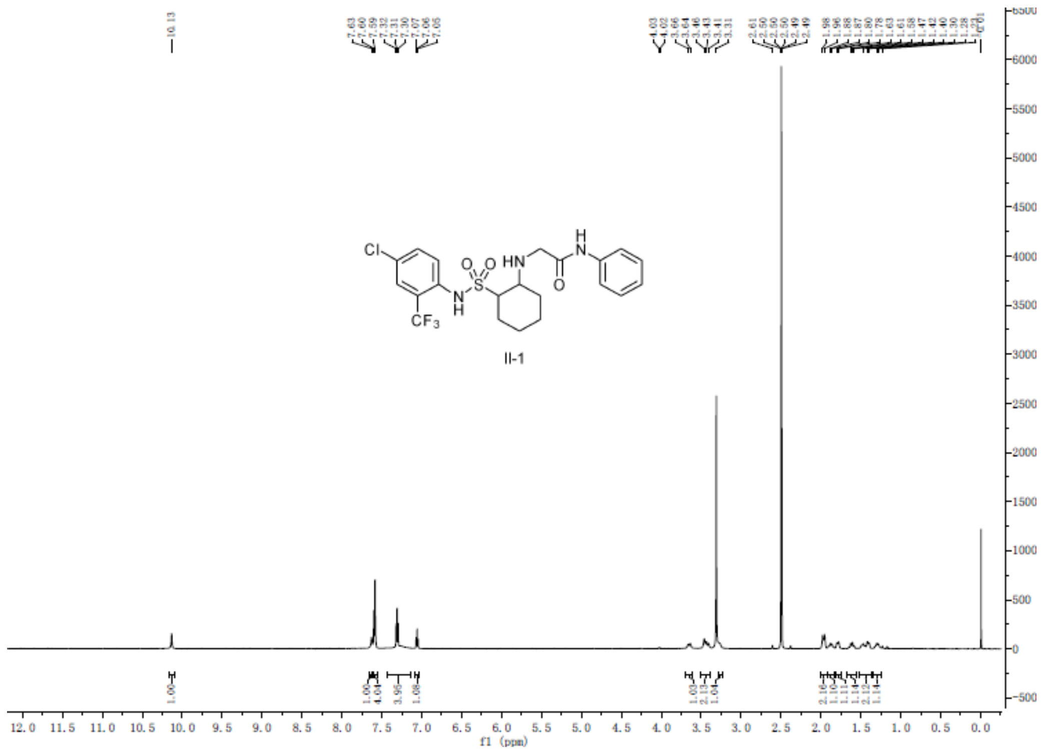

Figure S1-1 ^{1}H NMR spectrum of compound II-1

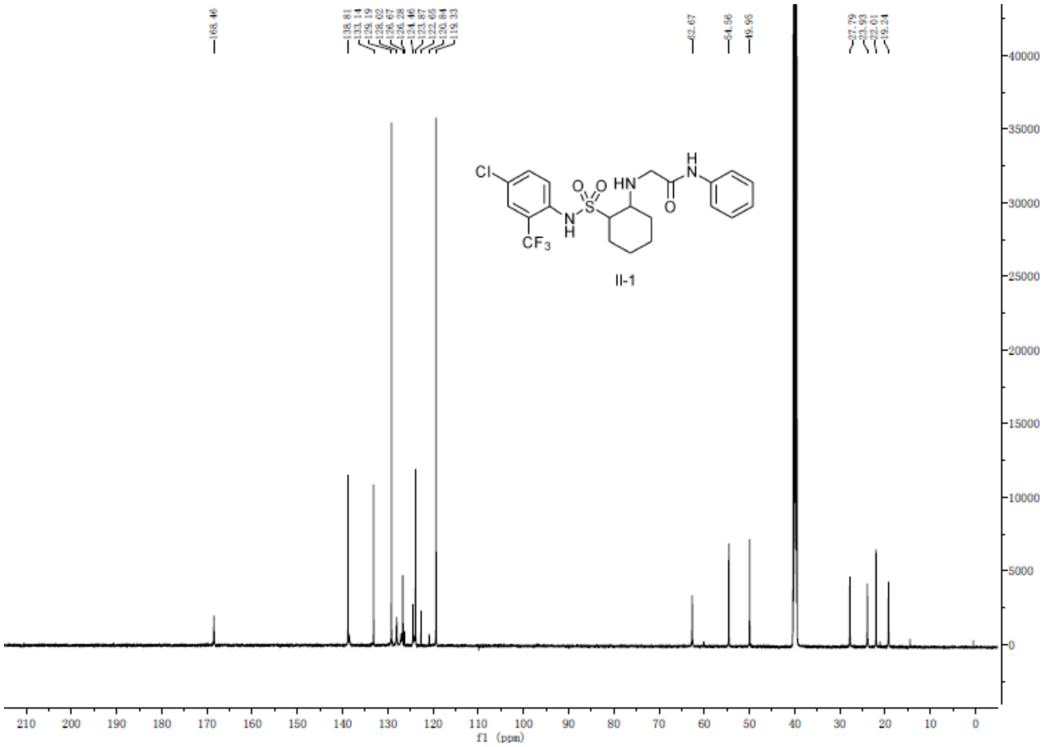

Figure S1-2 ^{13}C NMR spectrum of compound II-1.

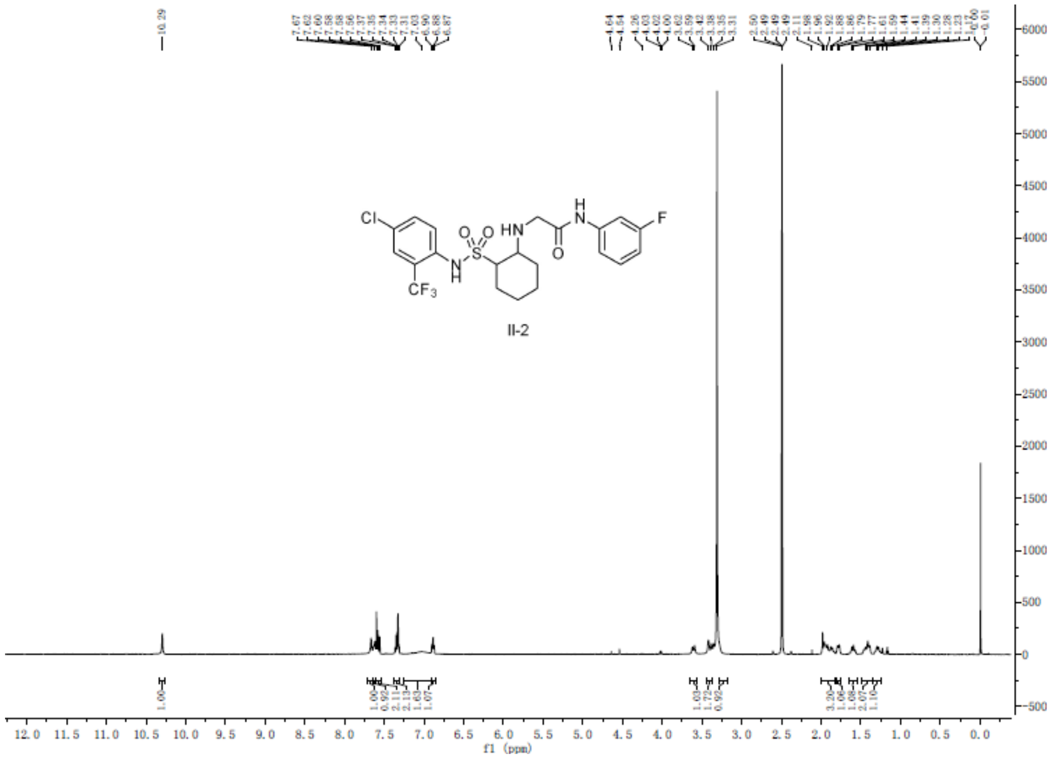

Figure S2-1 ^{1}H NMR spectrum of compound II-2.

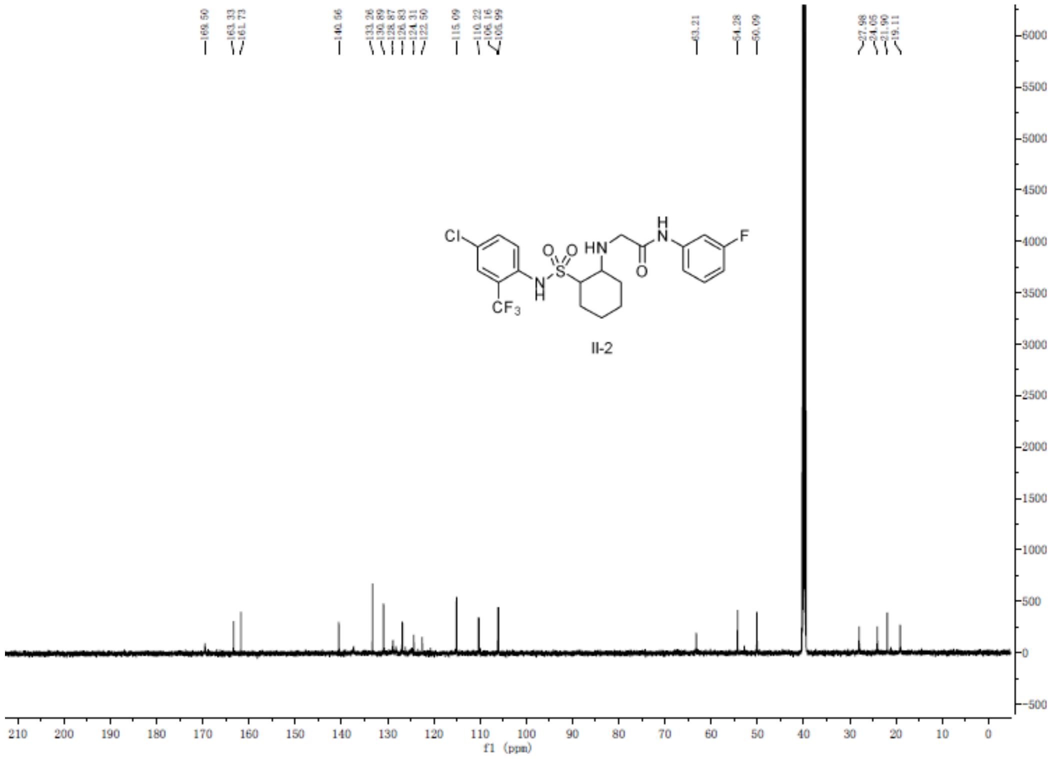

Figure S2-2 ^{13}C NMR spectrum of compound II-2.

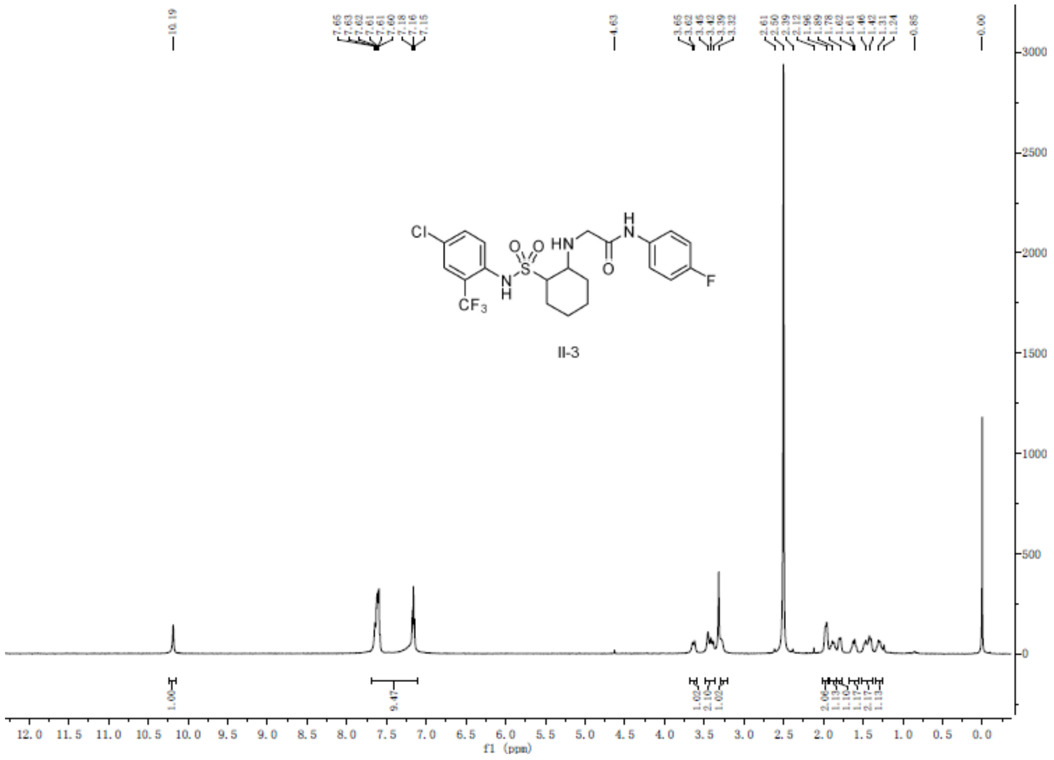

Figure S3-1 ^{1}H NMR spectrum of compound II-3.

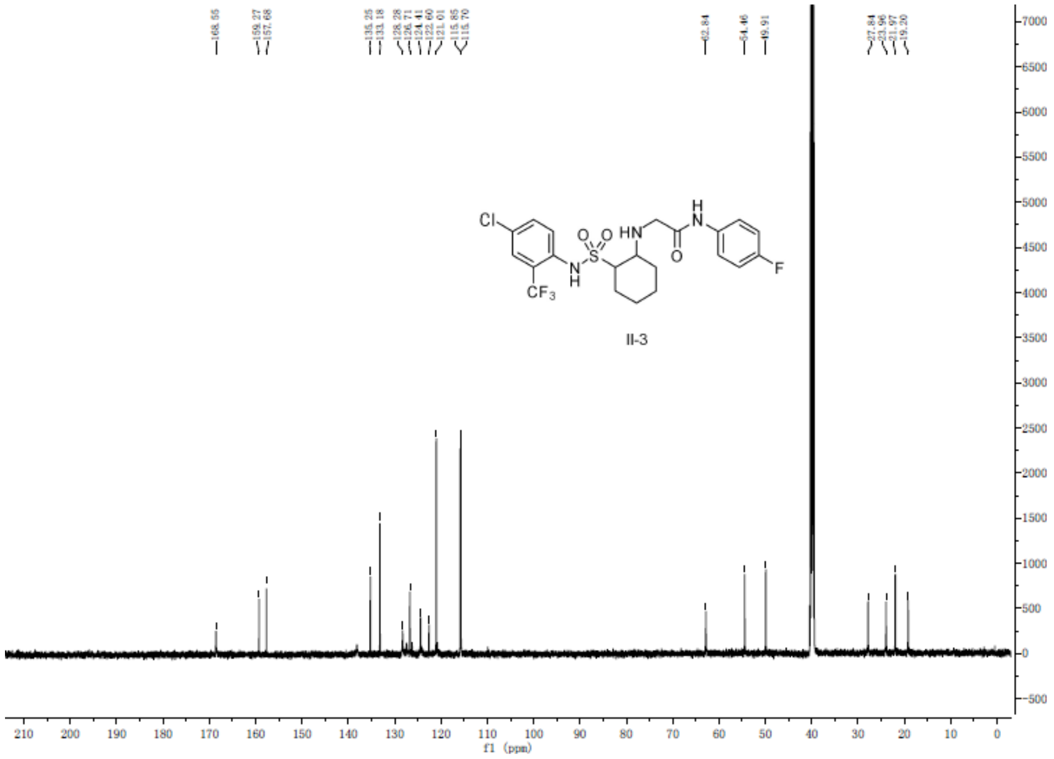

Figure S3-2 ^{13}C NMR spectrum of compound II-3.

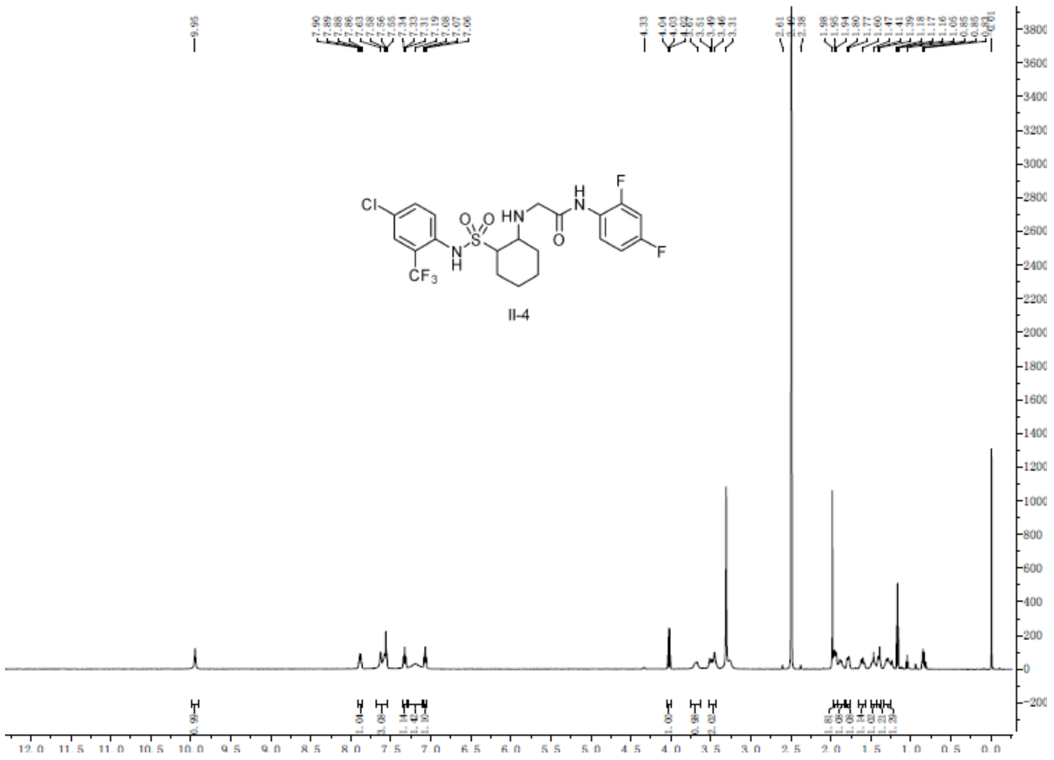

Figure S4-1 ^{1}H NMR spectrum of compound II-4.

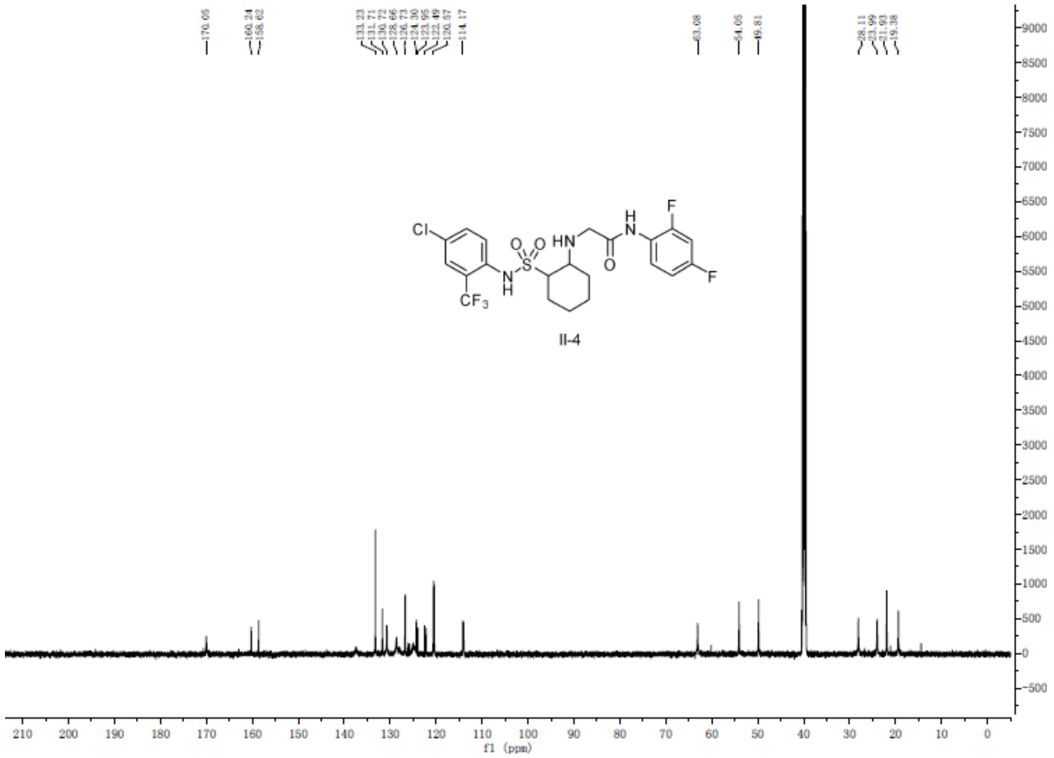

Figure S4-2 ^{13}C NMR spectrum of compound II-4.

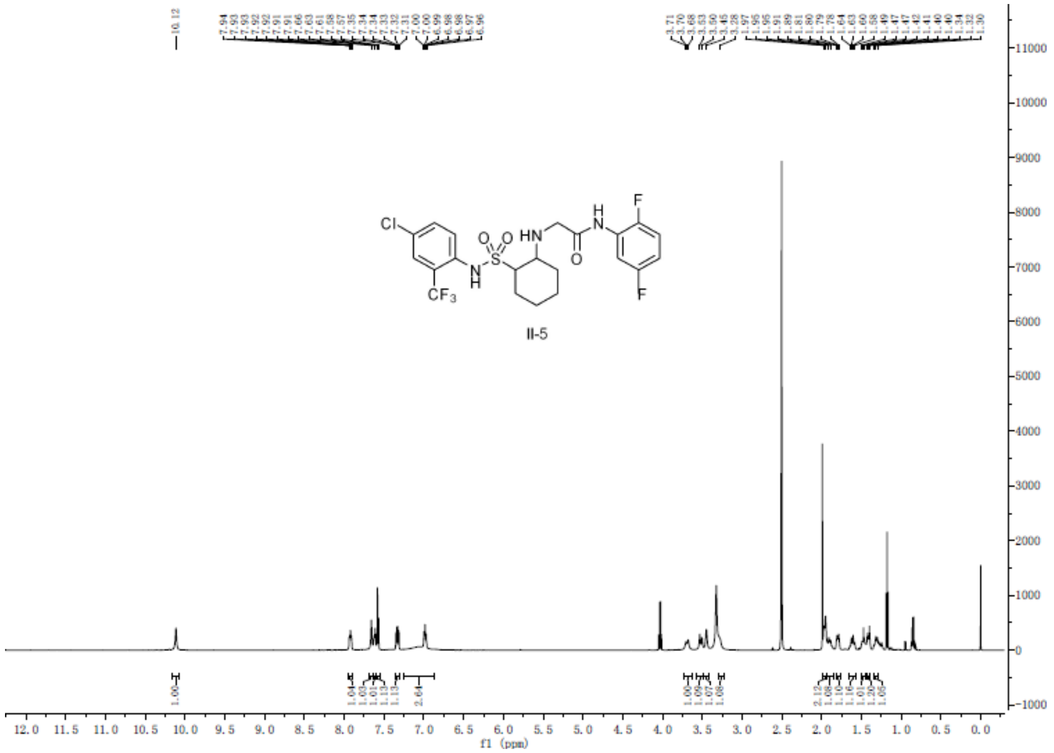

Figure S5-1 ^{1}H NMR spectrum of compound II-5.

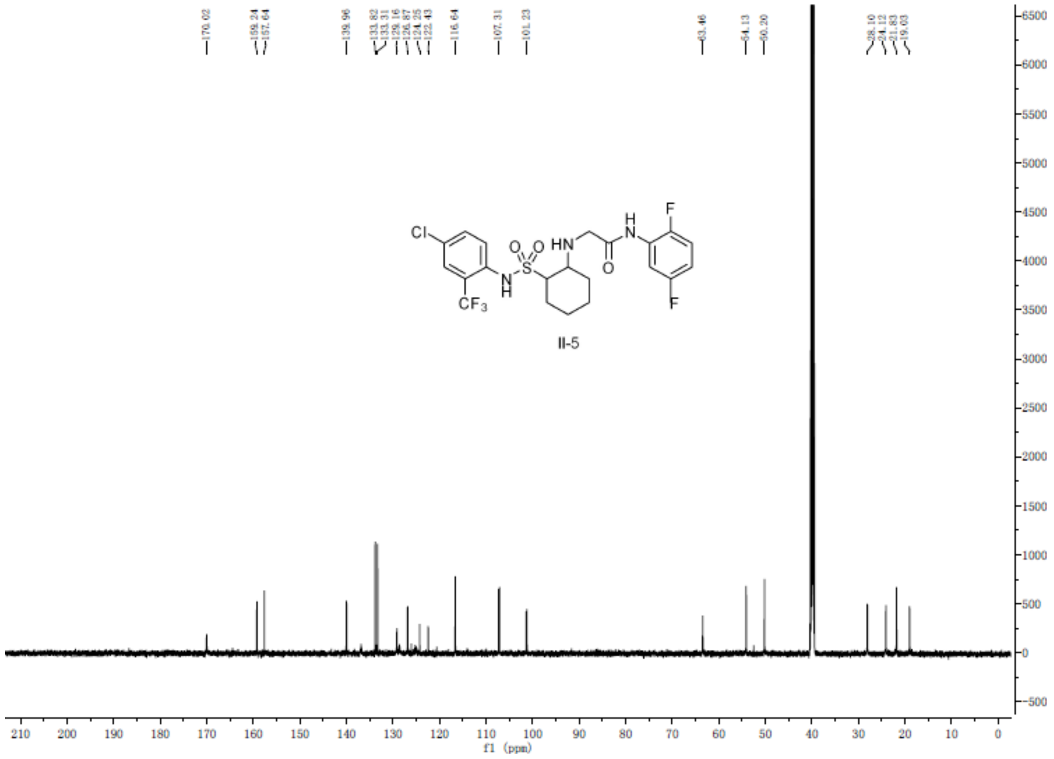

Figure S5-2 ^{13}C NMR spectrum of compound II-5.

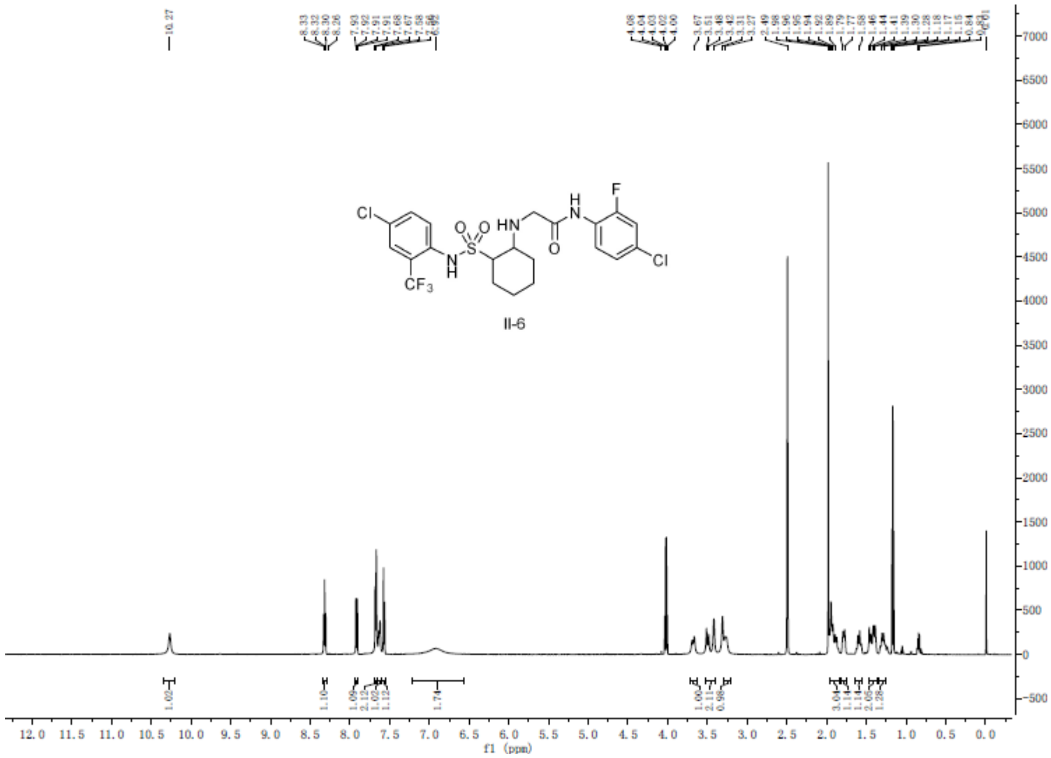

Figure S6-1 ^{1}H NMR spectrum of compound II-6.

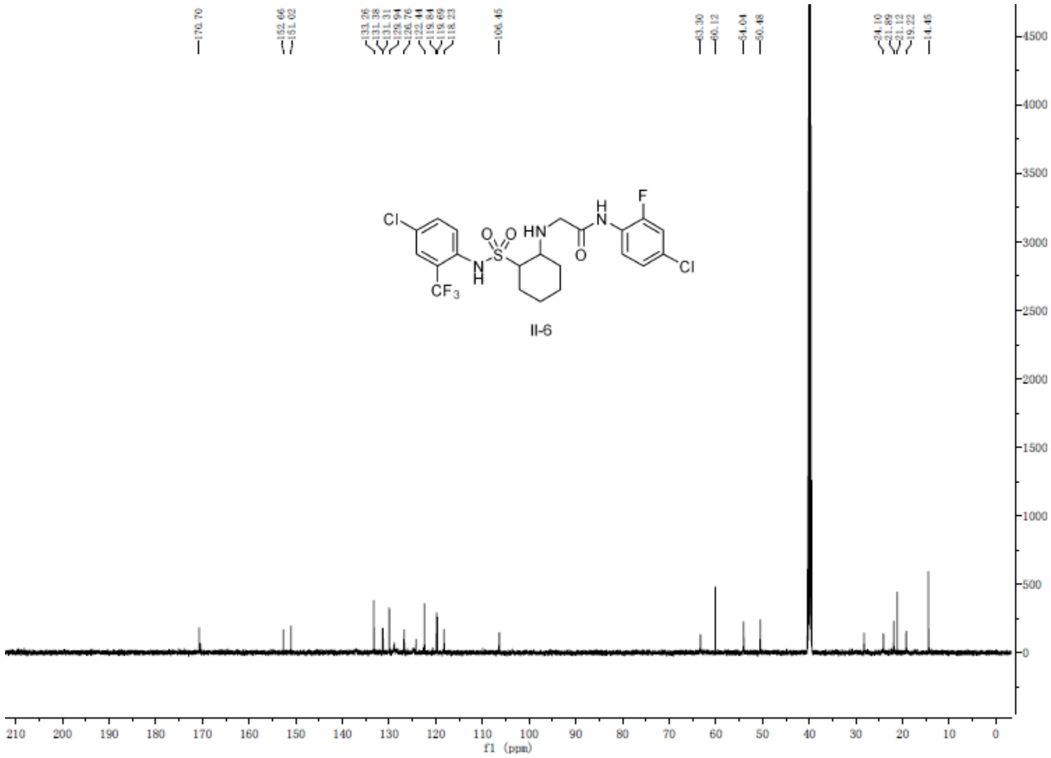

Figure S6-2 ^{13}C NMR spectrum of compound II-6.

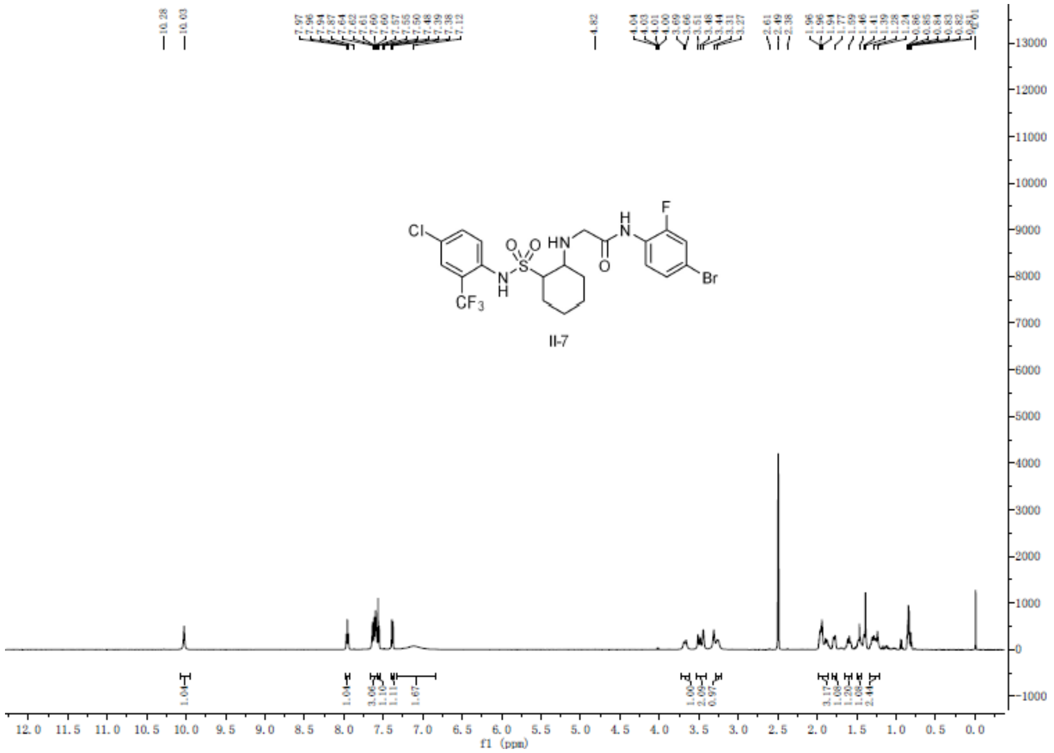

Figure S7-1 ^{1}H NMR spectrum of compound II-7.

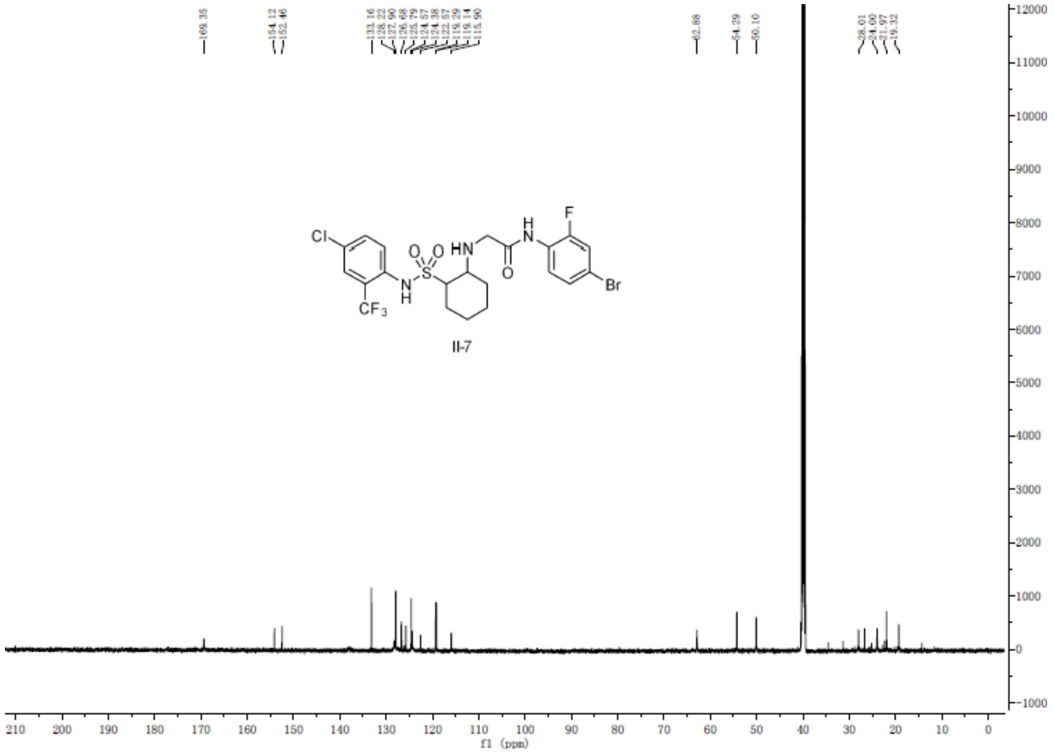

Figure S7-2 ^{13}C NMR spectrum of compound II-7.

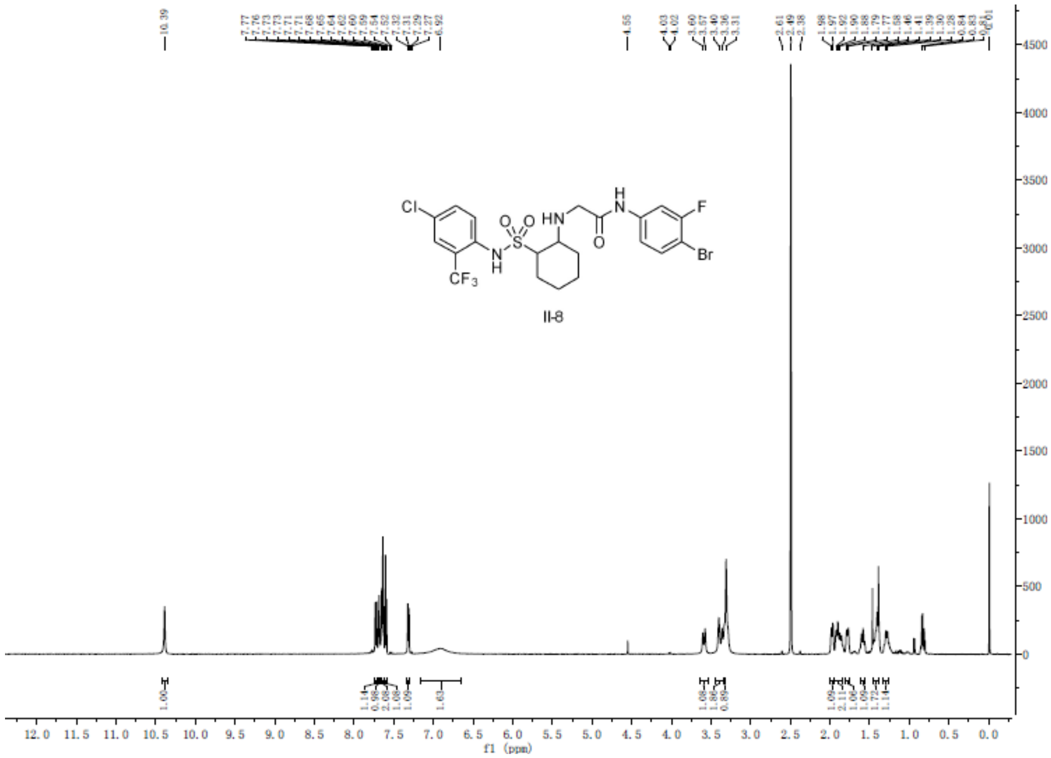

Figure S8-1 ^{1}H NMR spectrum of compound II-8.

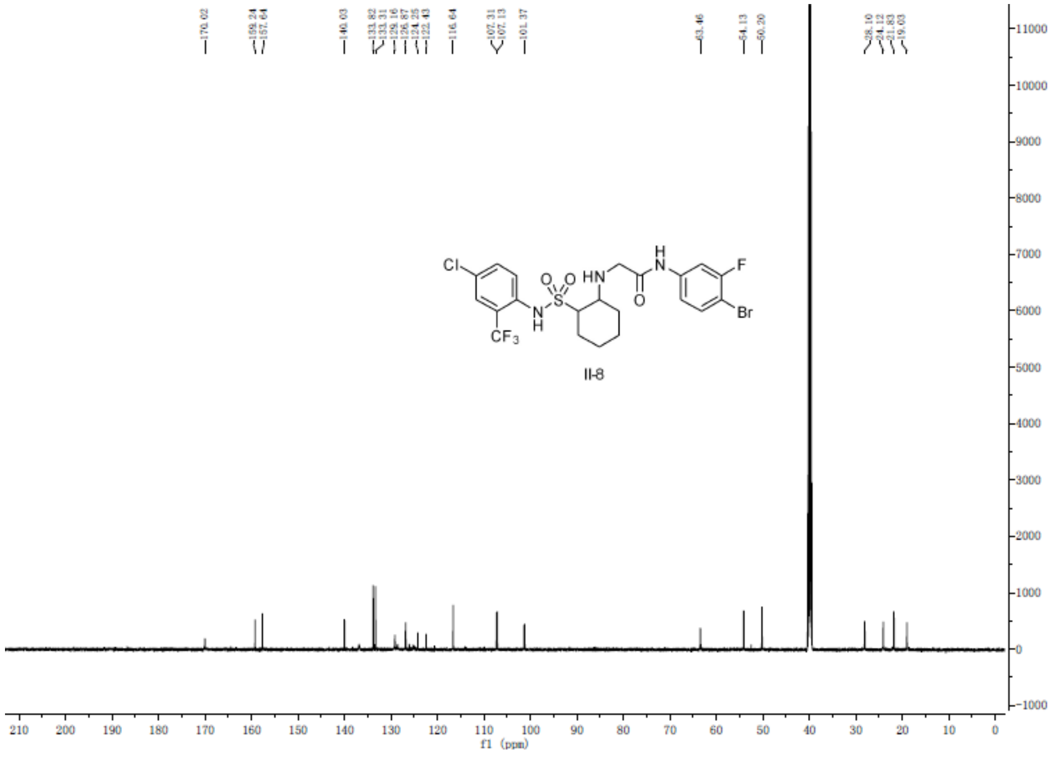

Figure S8-2 ^{13}C NMR spectrum of compound II-8.

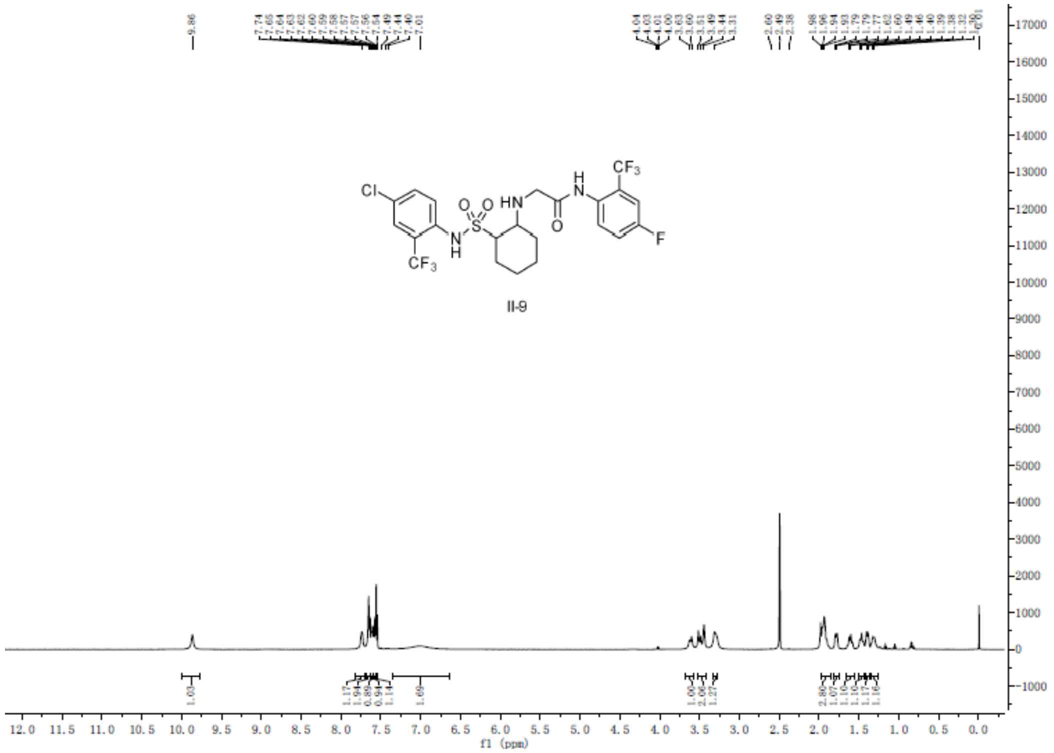

Figure S9-1 ^{1}H NMR spectrum of compound II-9.

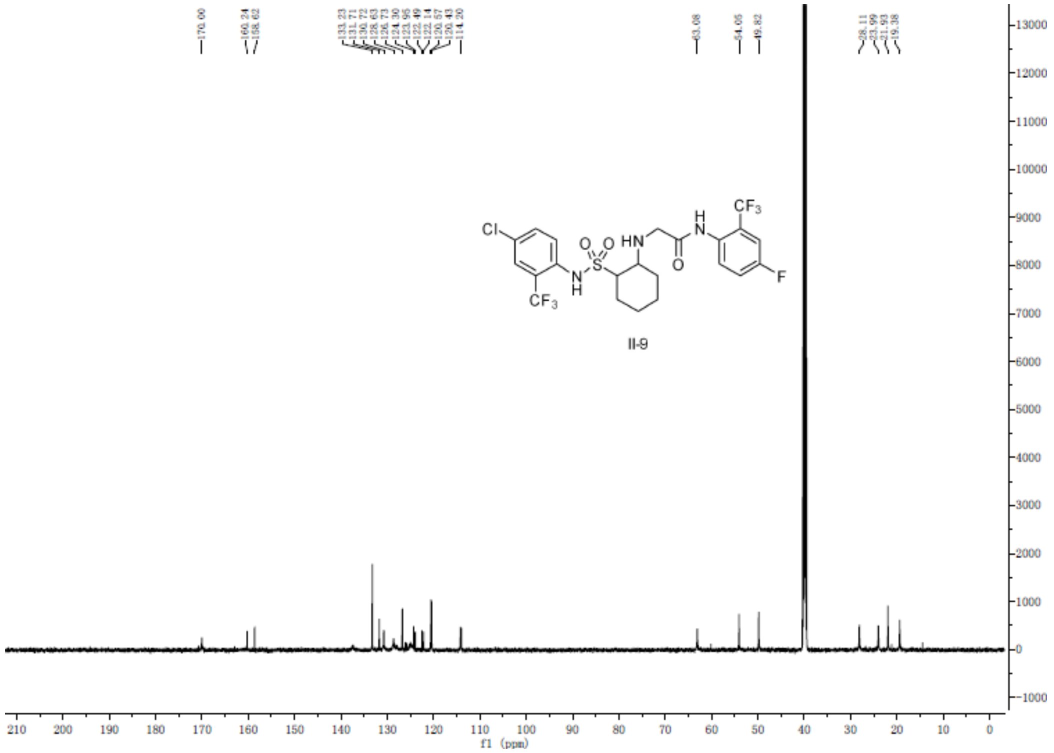

Figure S9-2 ^{13}C NMR spectrum of compound II-9.

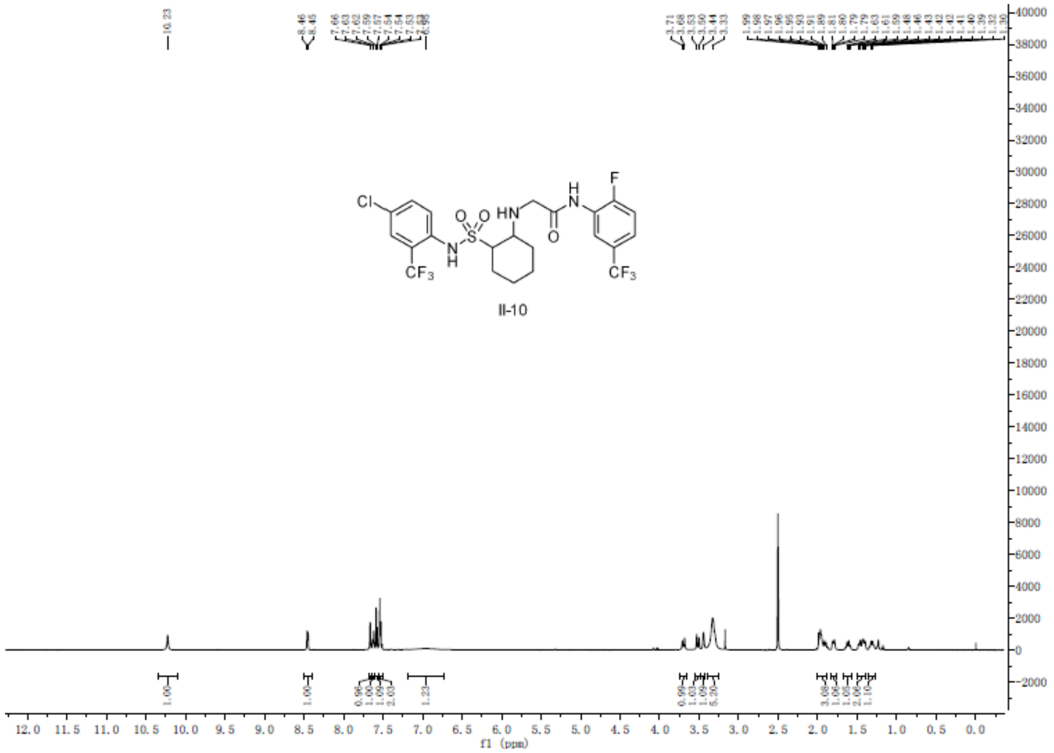

Figure S10-1 ^{1}H NMR spectrum of compound II-10.

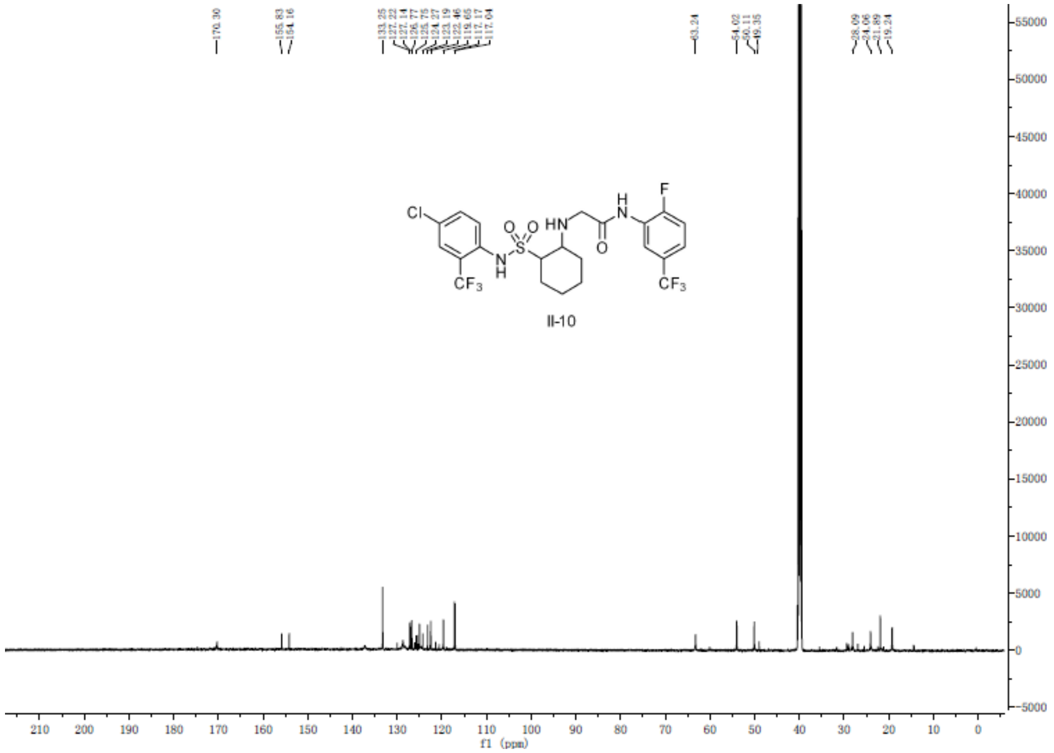

Figure S10-2 ^{13}C NMR spectrum of compound II-10.

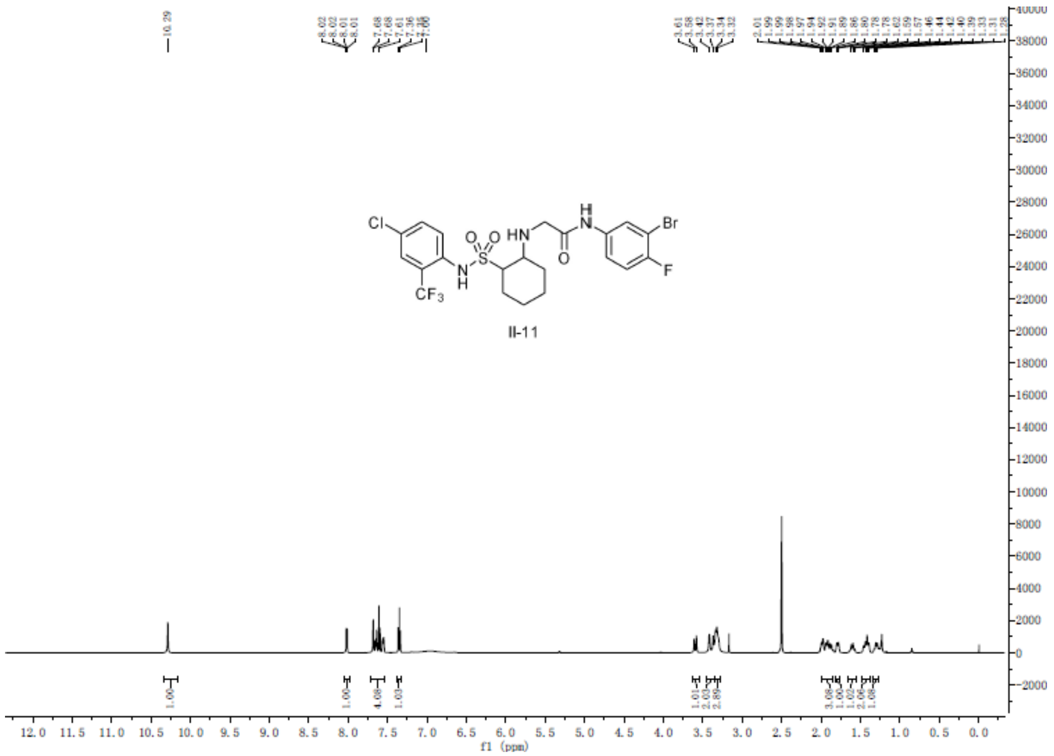

Figure S11-1 ^{1}H NMR spectrum of compound II-11.

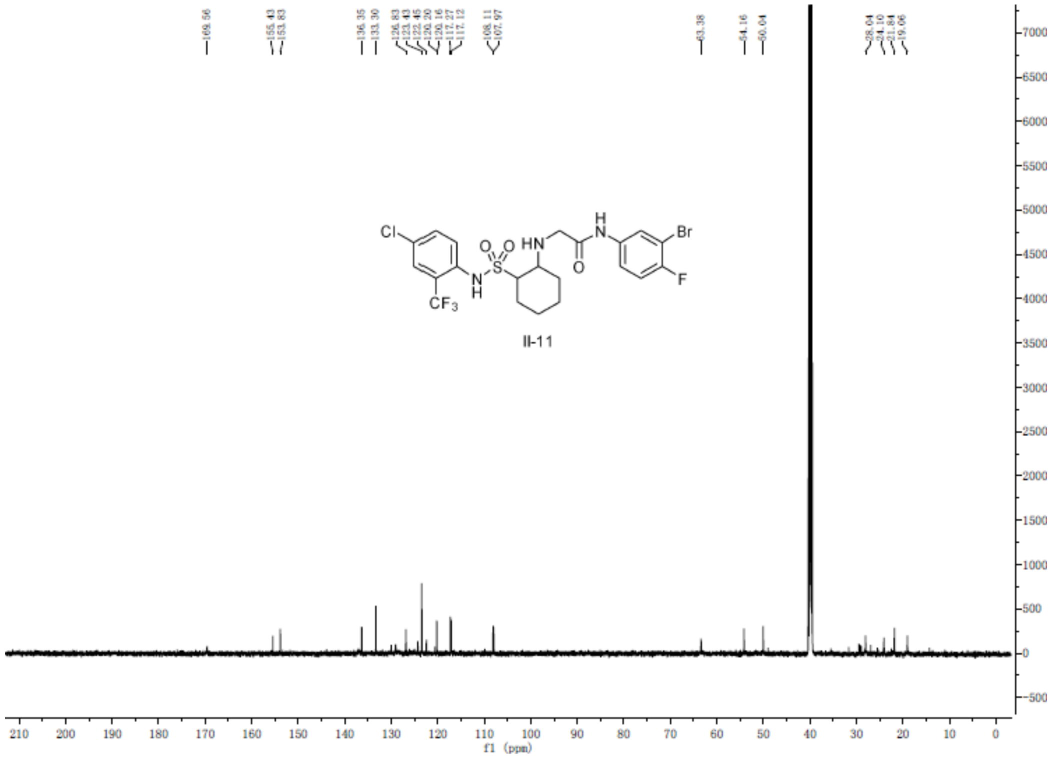

Figure S11-2 ^{13}C NMR spectrum of compound II-11.

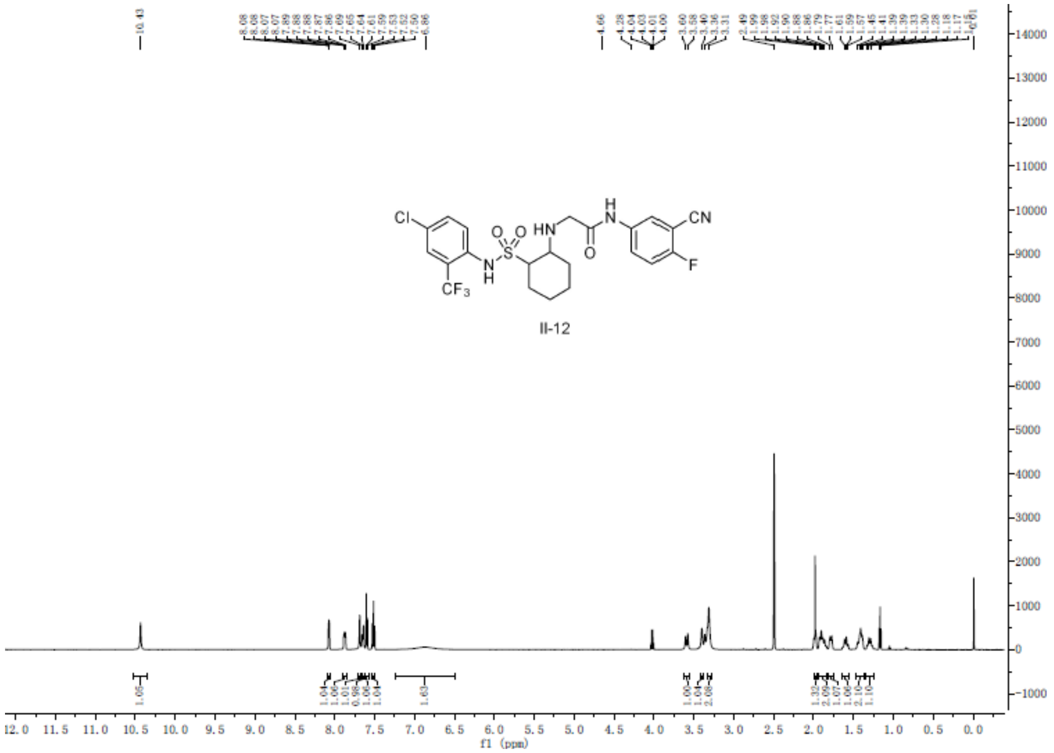

Figure S12-1 ^{1}H NMR spectrum of compound II-12.

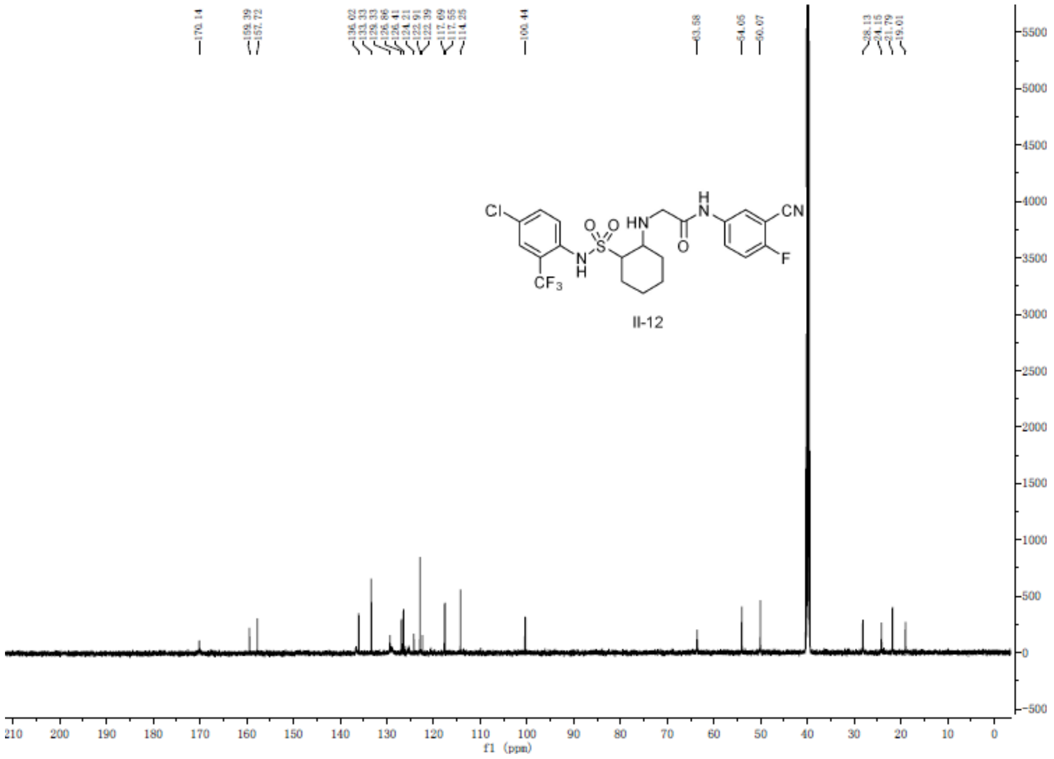

Figure S12-2 ^{13}C NMR spectrum of compound II-12.

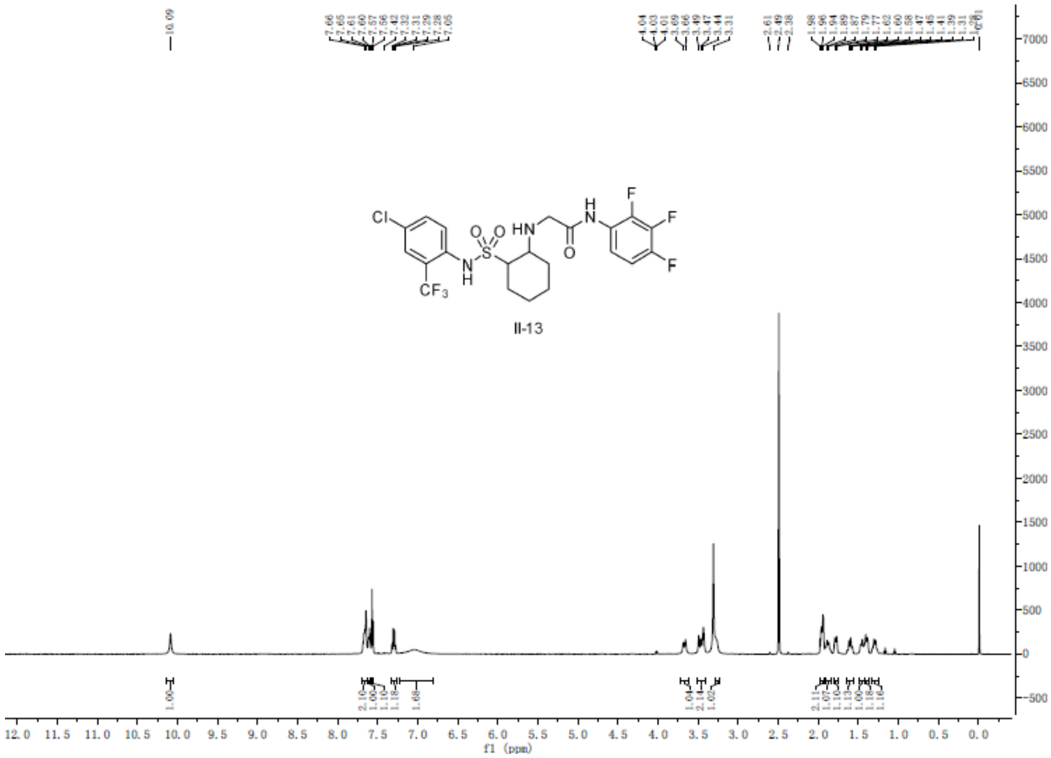

Figure S13-1 ^{1}H NMR spectrum of compound II-13.

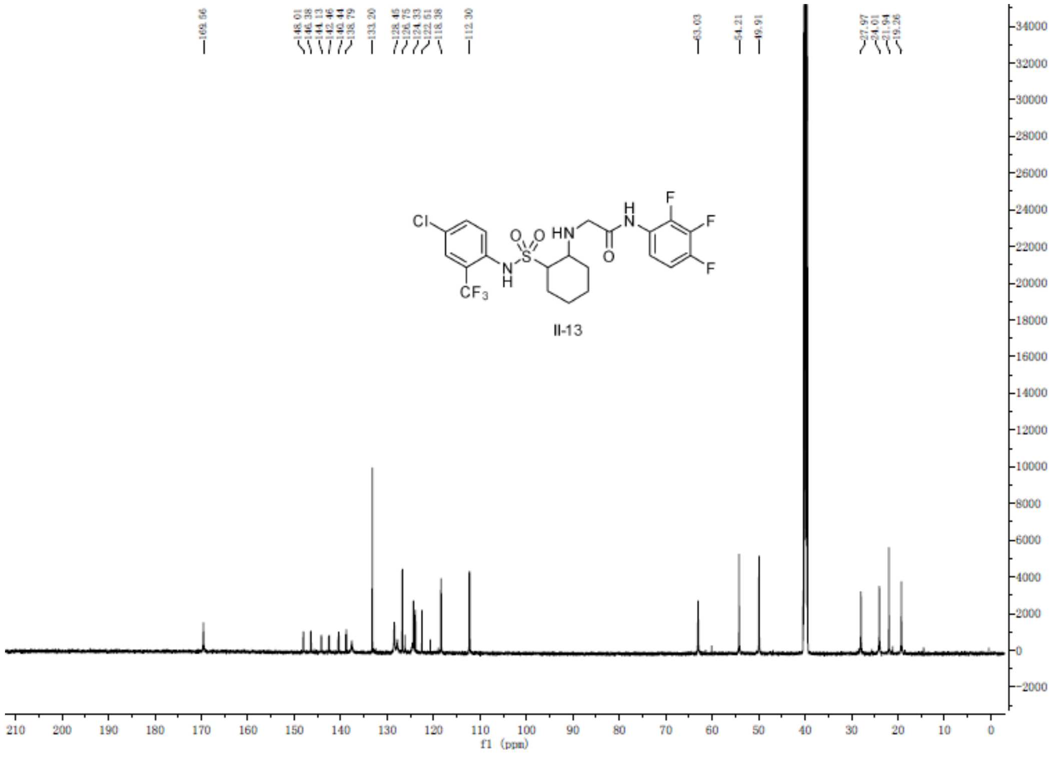

Figure S13-2 ^{13}C NMR spectrum of compound II-13.

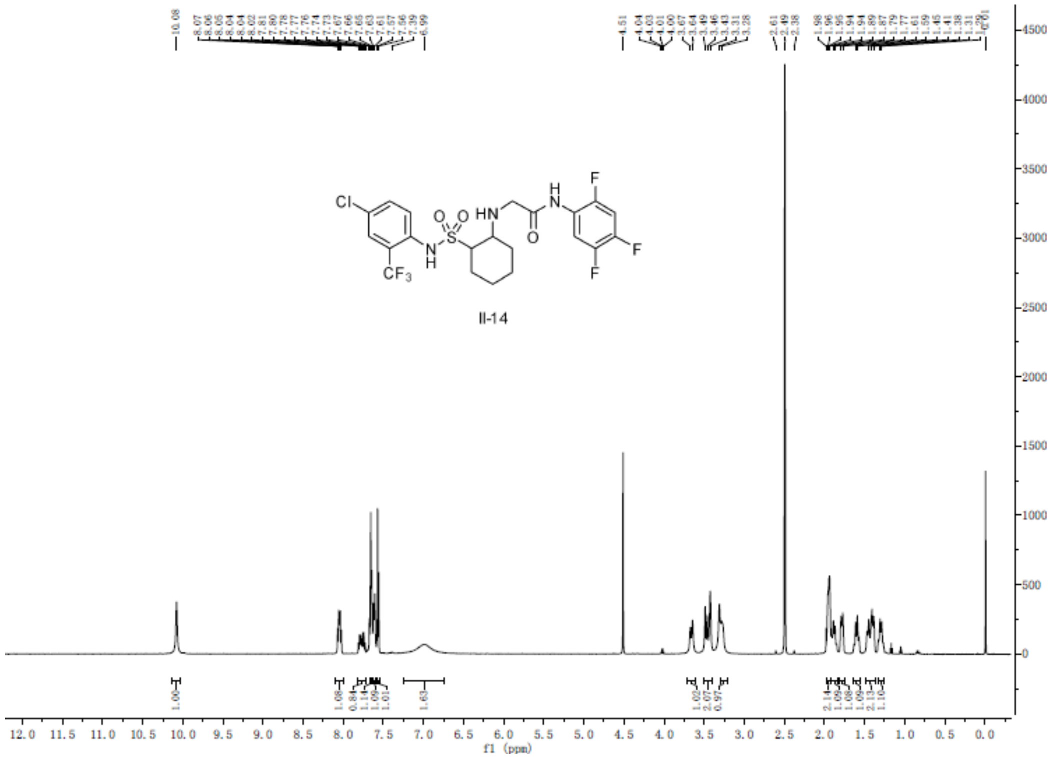

Figure S14-1 ^{1}H NMR spectrum of compound II-14.

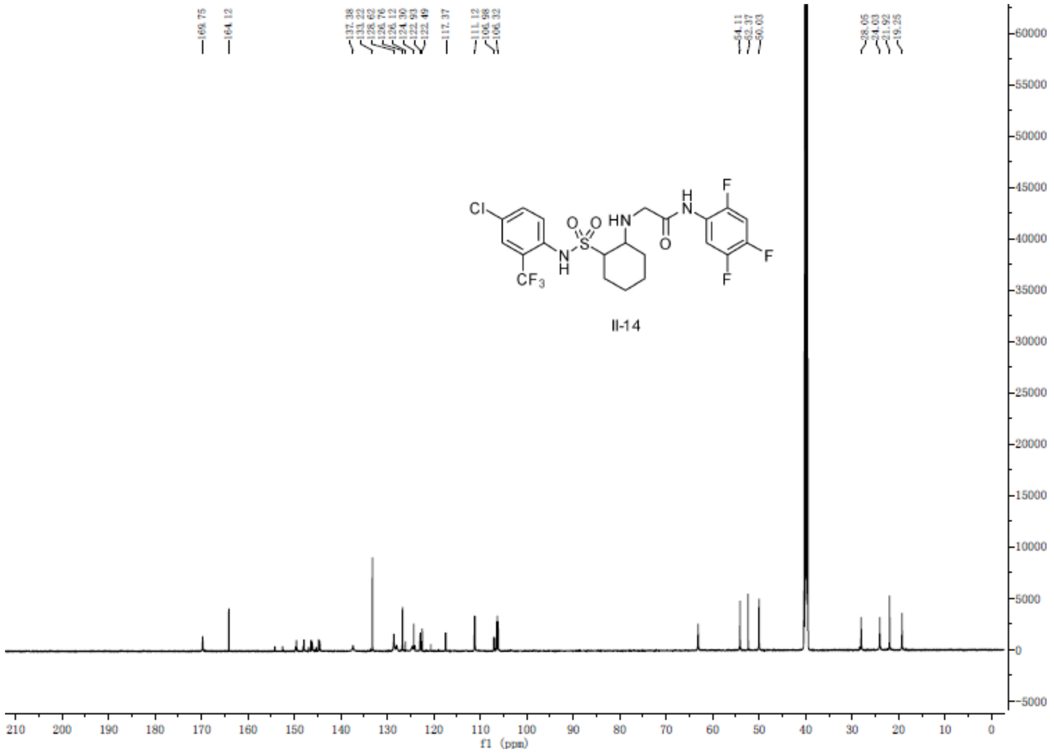

Figure S14-2 ^{13}C NMR spectrum of compound II-14.

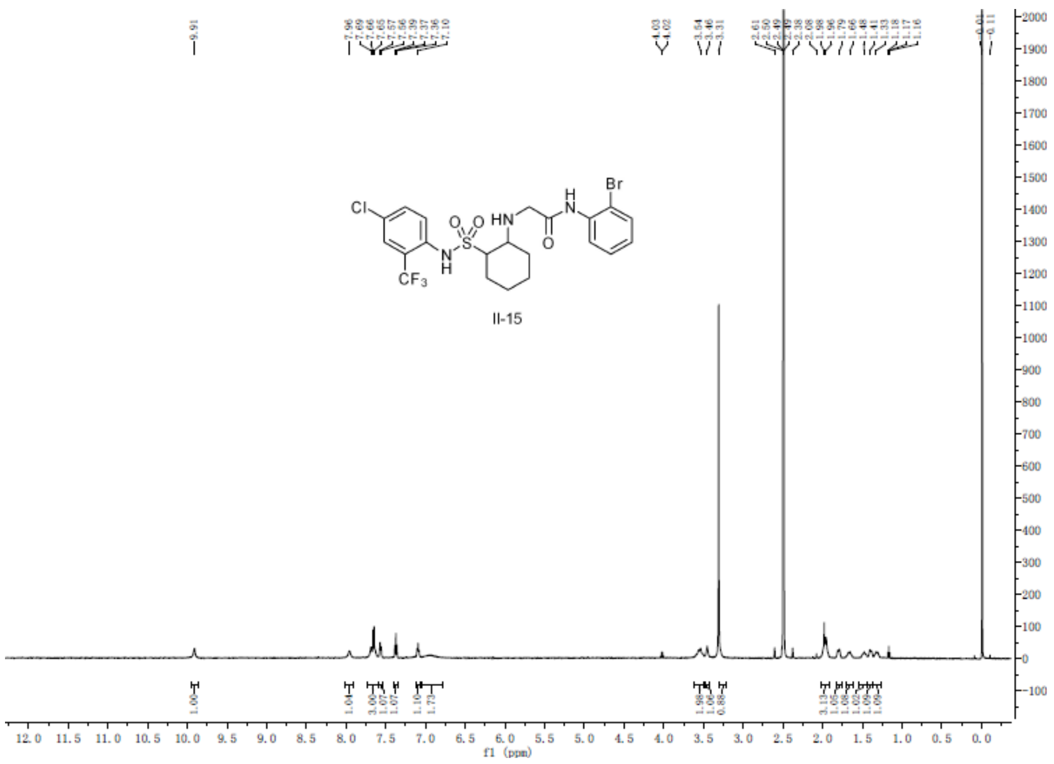

Figure S15-1 ^{1}H NMR spectrum of compound II-15.

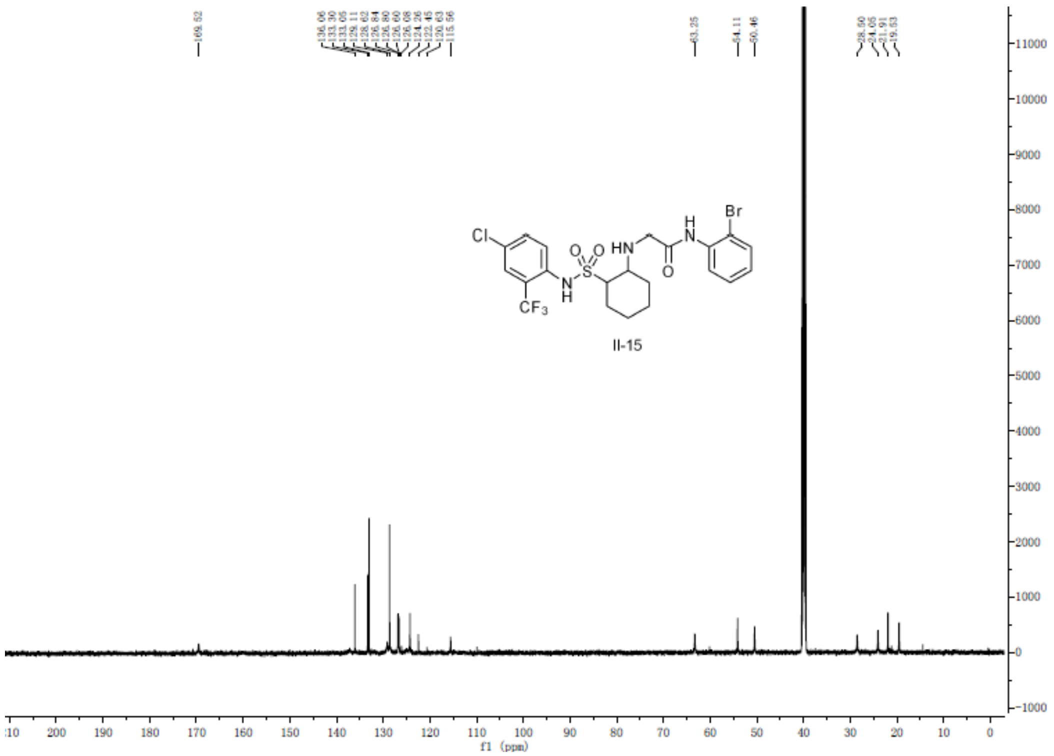

Figure S15-2 ^{13}C NMR spectrum of compound II-15.

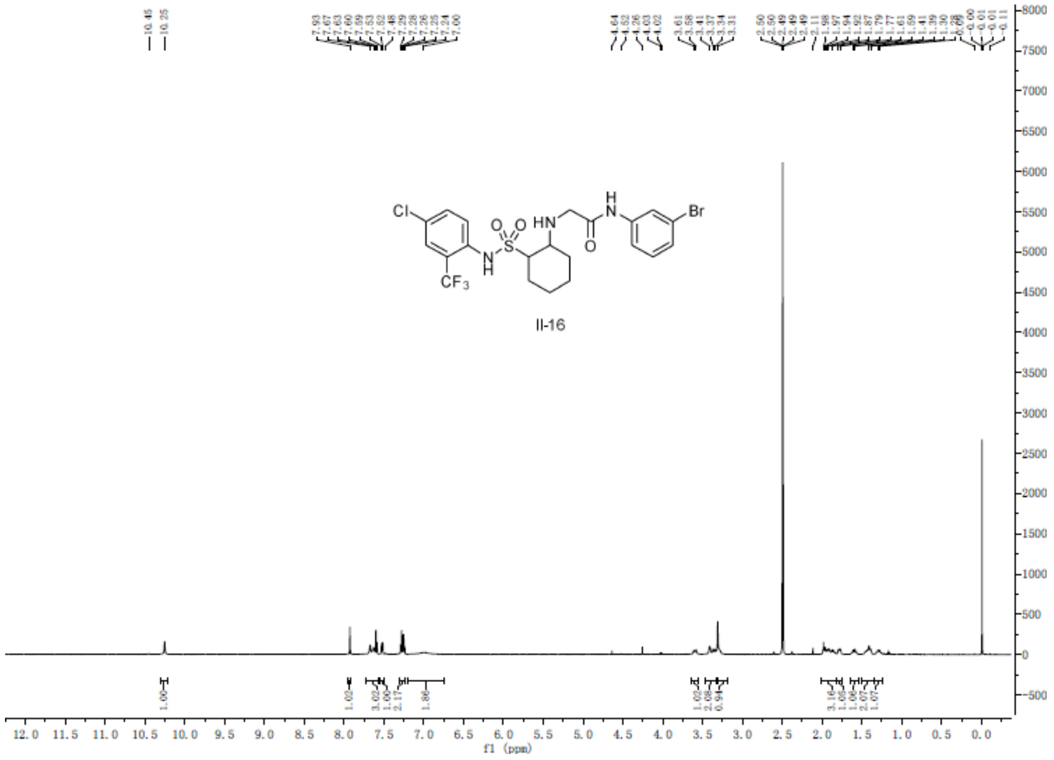

Figure S16-1 ^{1}H NMR spectrum of compound II-16.

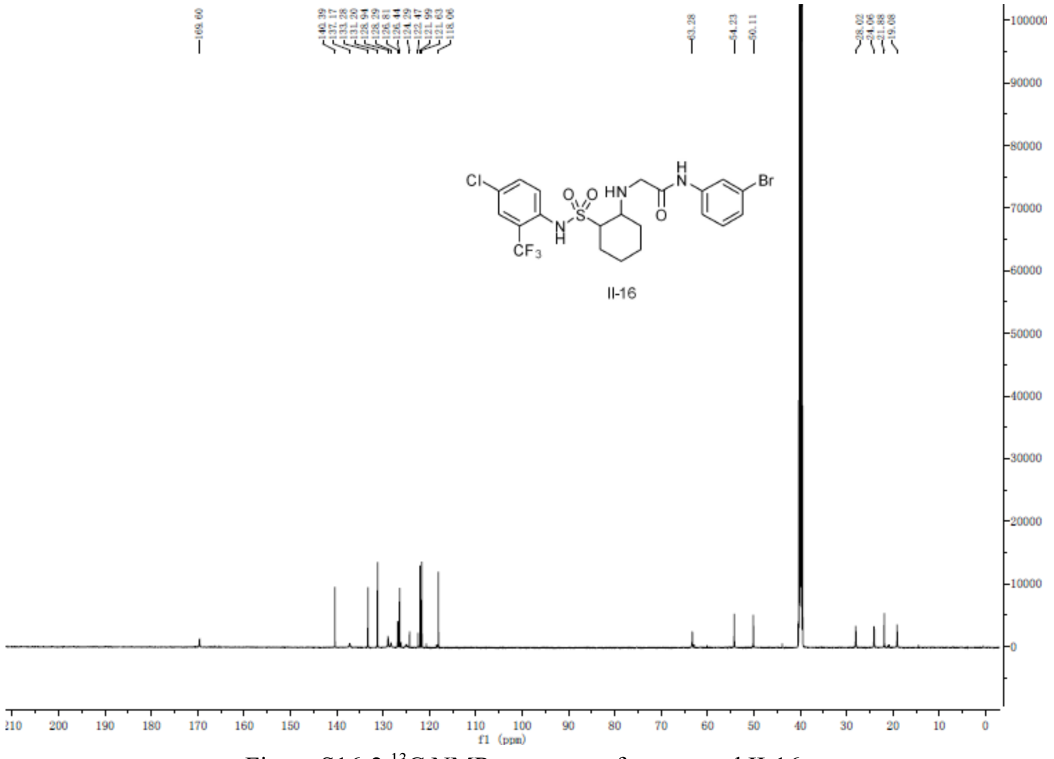

Figure S16-2 ^{13}C NMR spectrum of compound II-16.

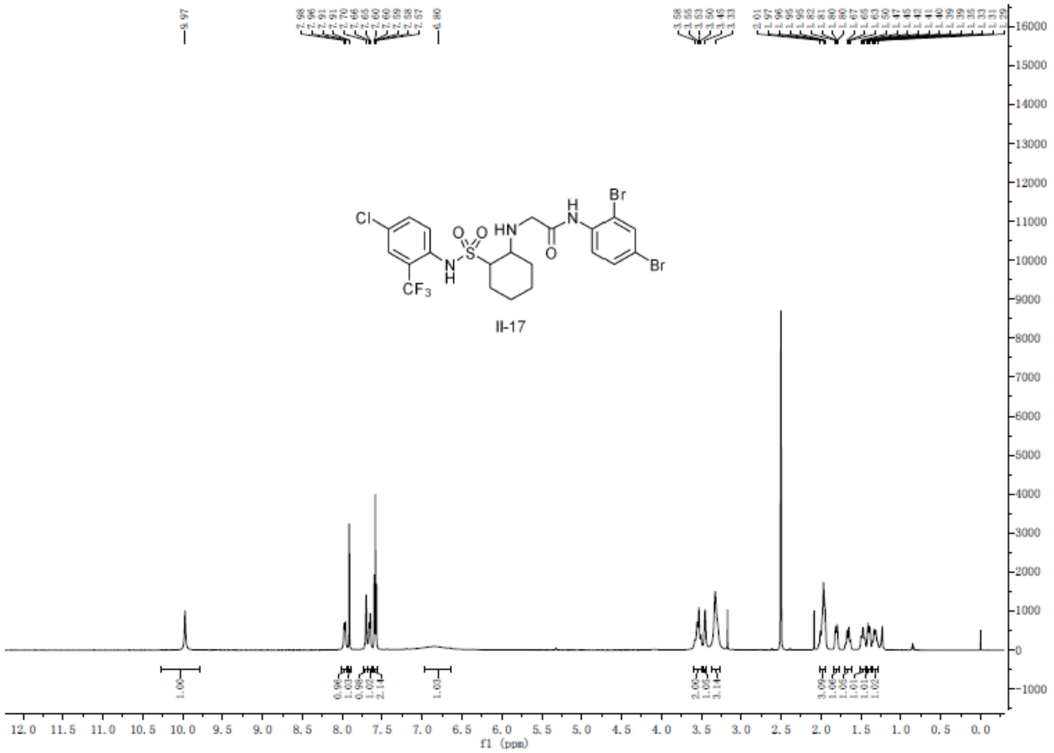

Figure S17-1 ^{1}H NMR spectrum of compound II-17.

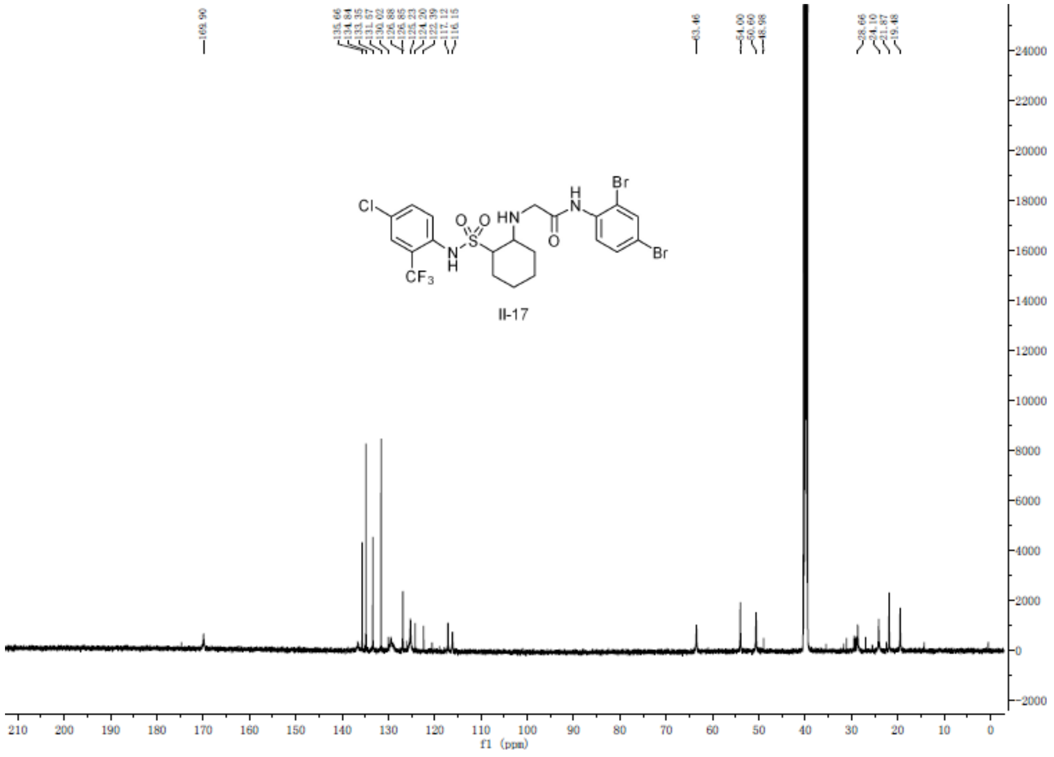

Figure S17-2 ^{13}C NMR spectrum of compound II-17.

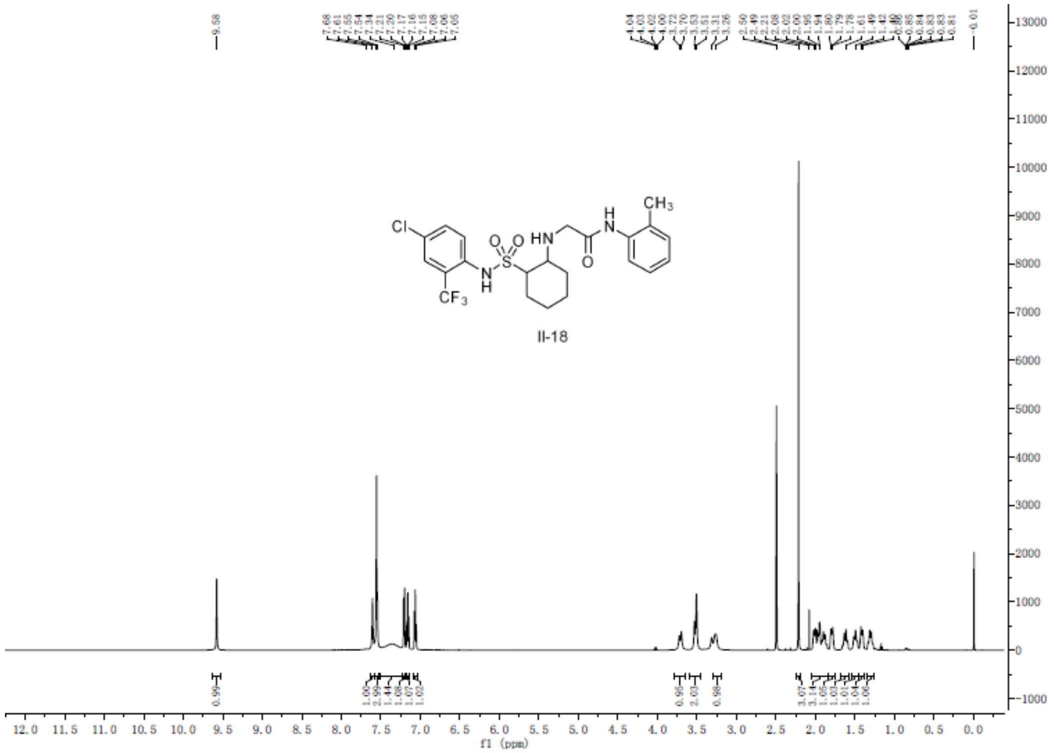

Figure S18-1 ^{1}H NMR spectrum of compound II-18.

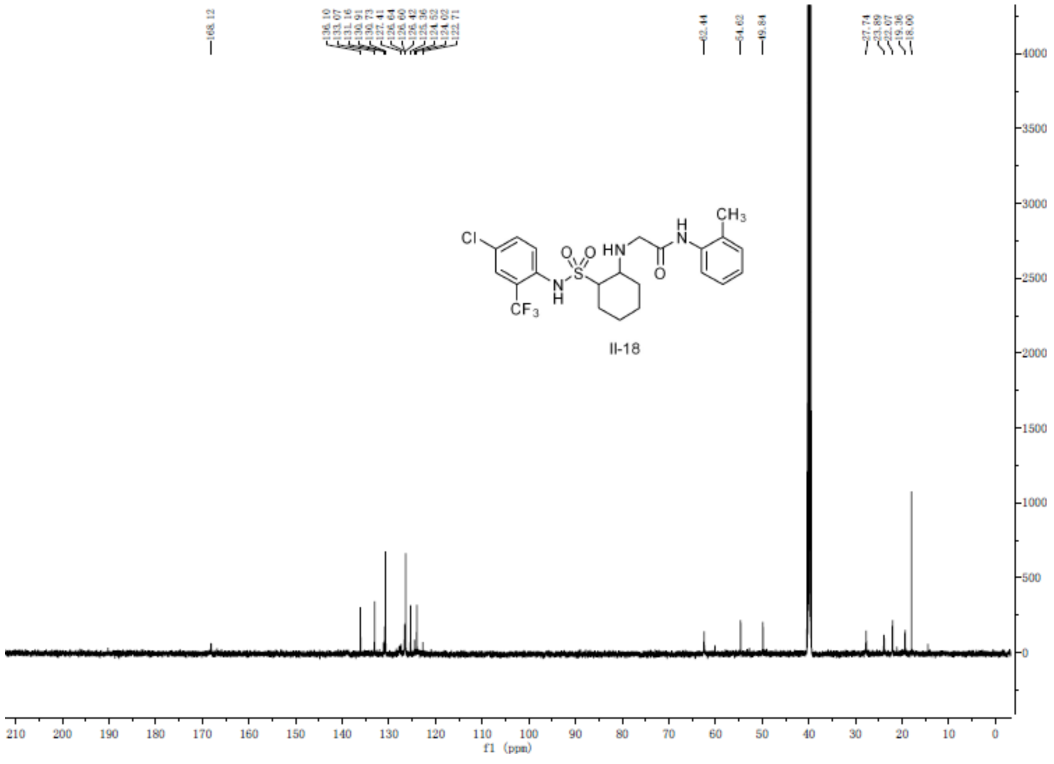

Figure S18-2 ^{13}C NMR spectrum of compound II-18.

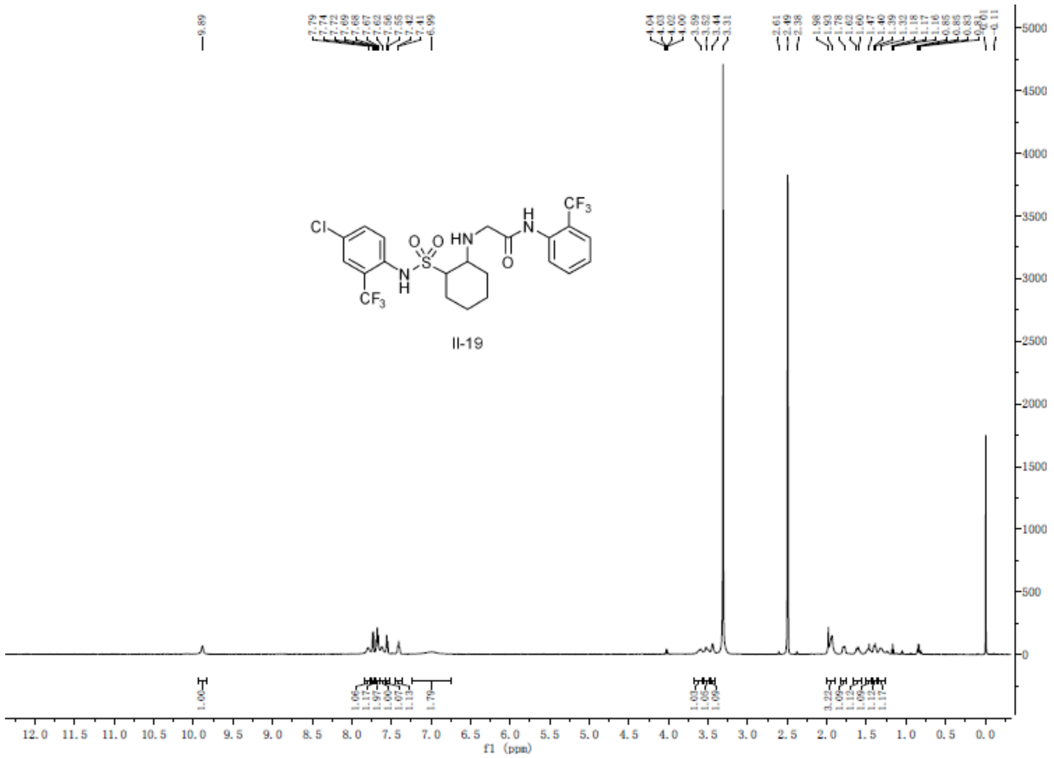

Figure S19-1 ^{1}H NMR spectrum of compound II-19.

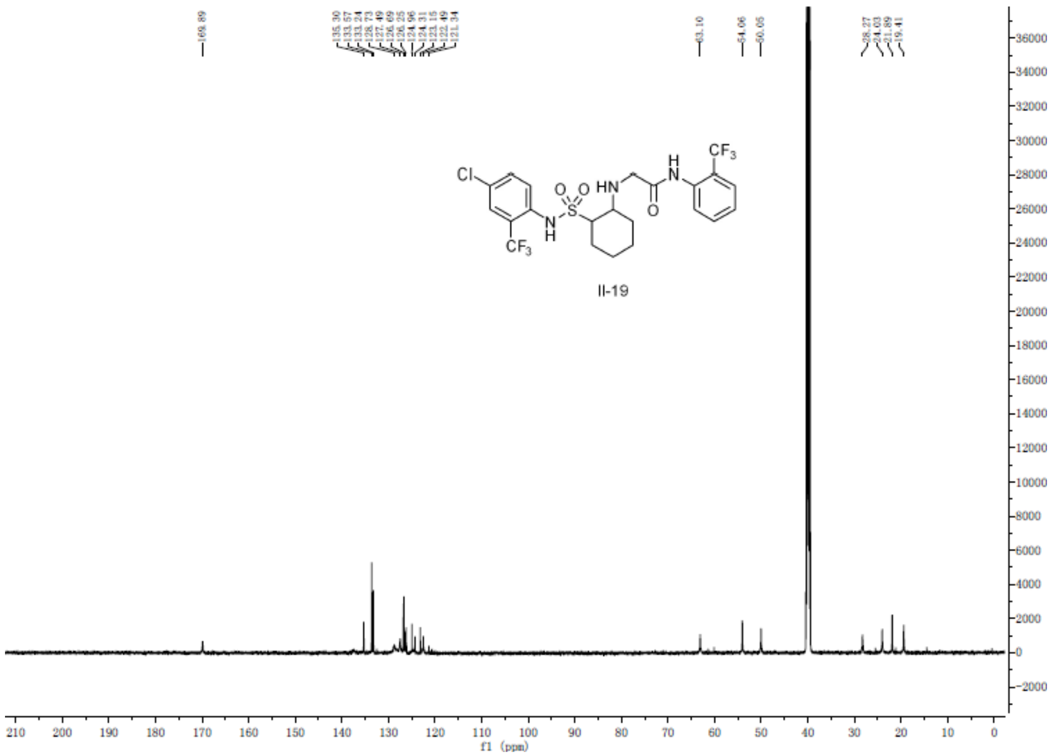

Figure S19-2 ^{13}C NMR spectrum of compound II-19.

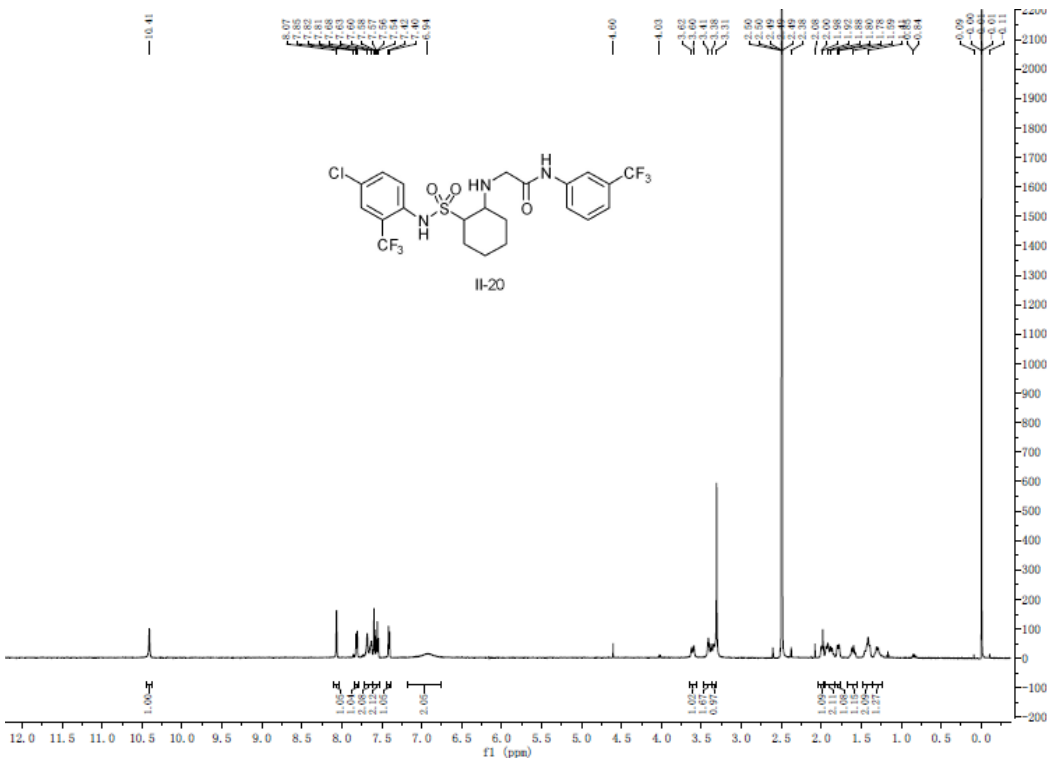

Figure S20-1 ^{1}H NMR spectrum of compound II-20.

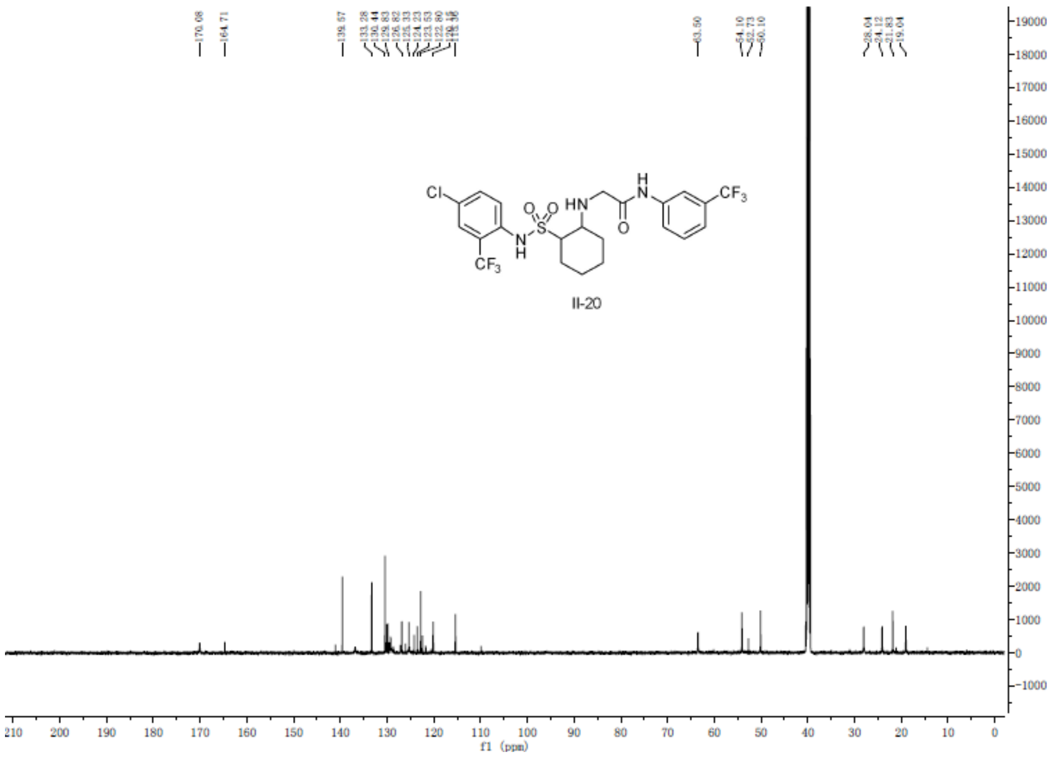

Figure S20-2 ^{13}C NMR spectrum of compound II-20.

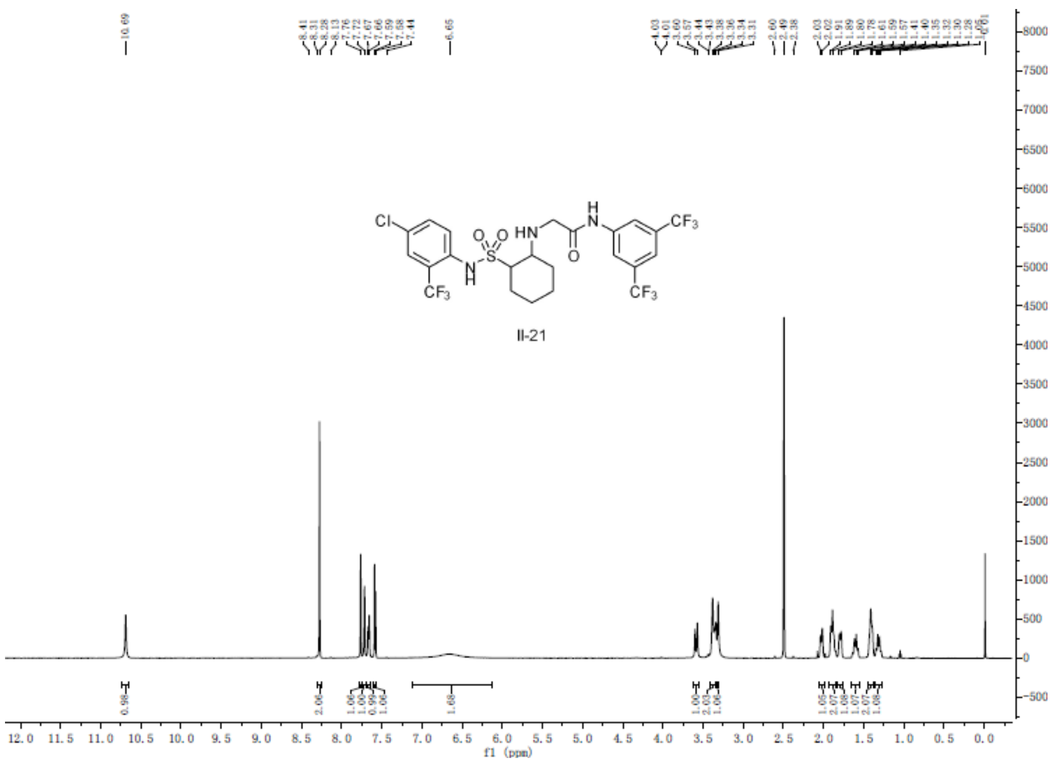

Figure S21-1 ^{1}H NMR spectrum of compound II-21.

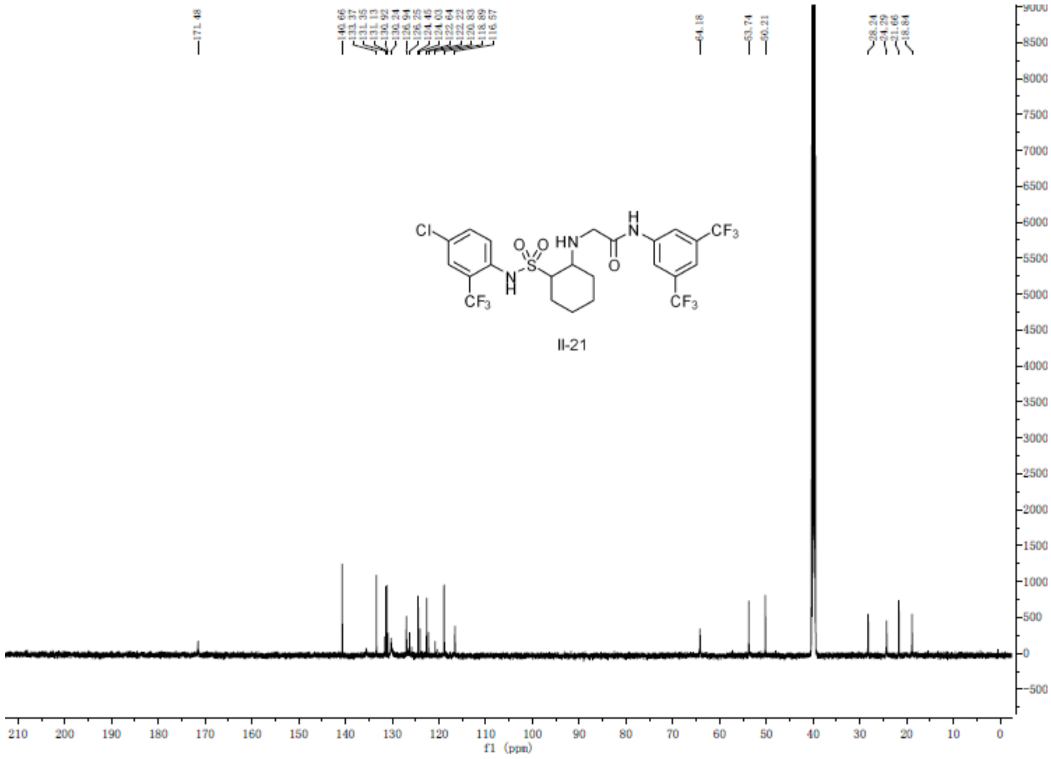

Figure S21-2 ^{13}C NMR spectrum of compound II-21.

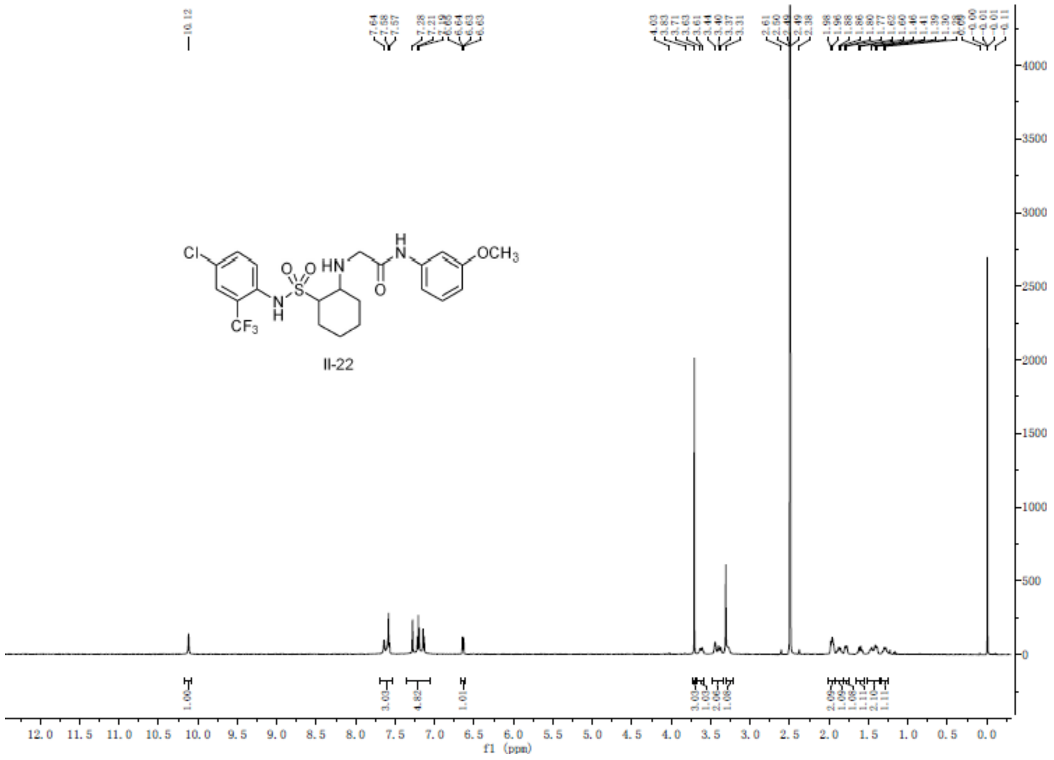

Figure S22-1 ^{1}H NMR spectrum of compound II-22.

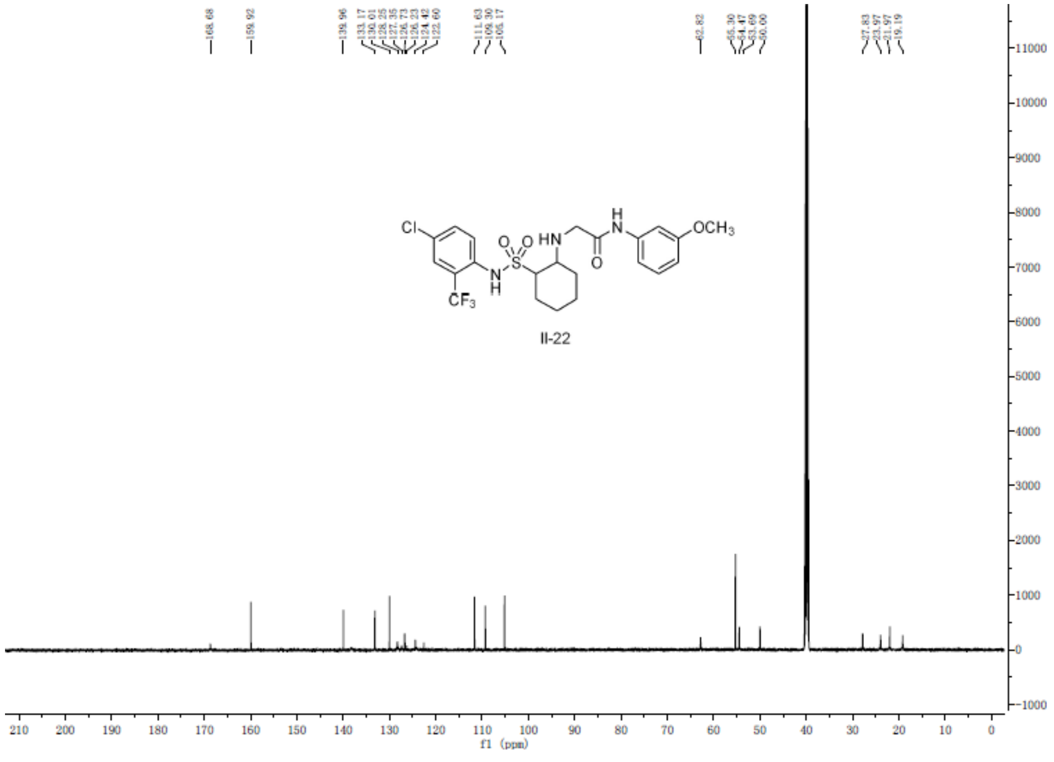

Figure S22-2 ^{13}C NMR spectrum of compound II-22.

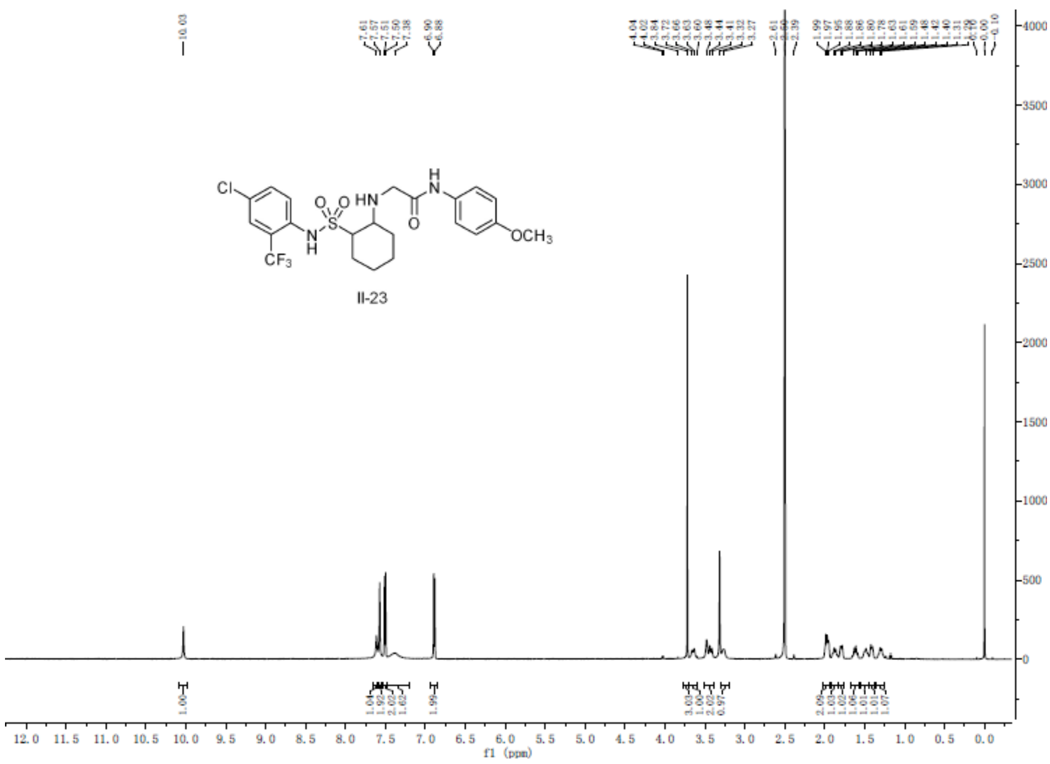

Figure S23-1 ^{1}H NMR spectrum of compound II-23.

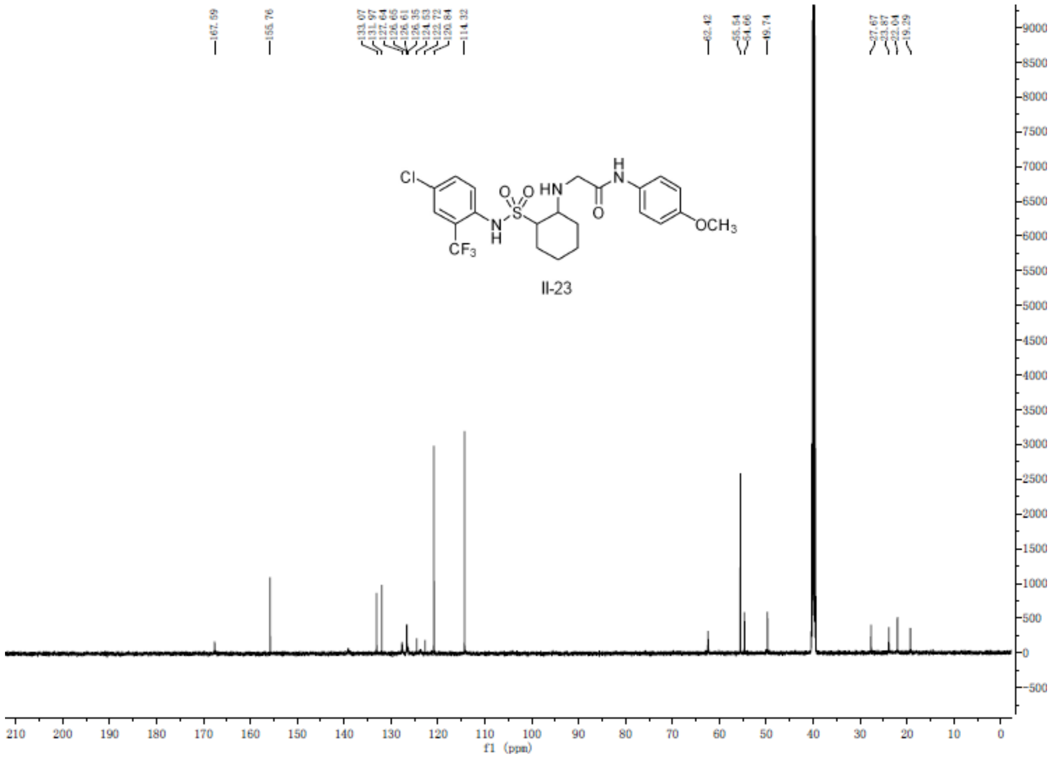

Figure S23-2 ^{13}C NMR spectrum of compound II-23.

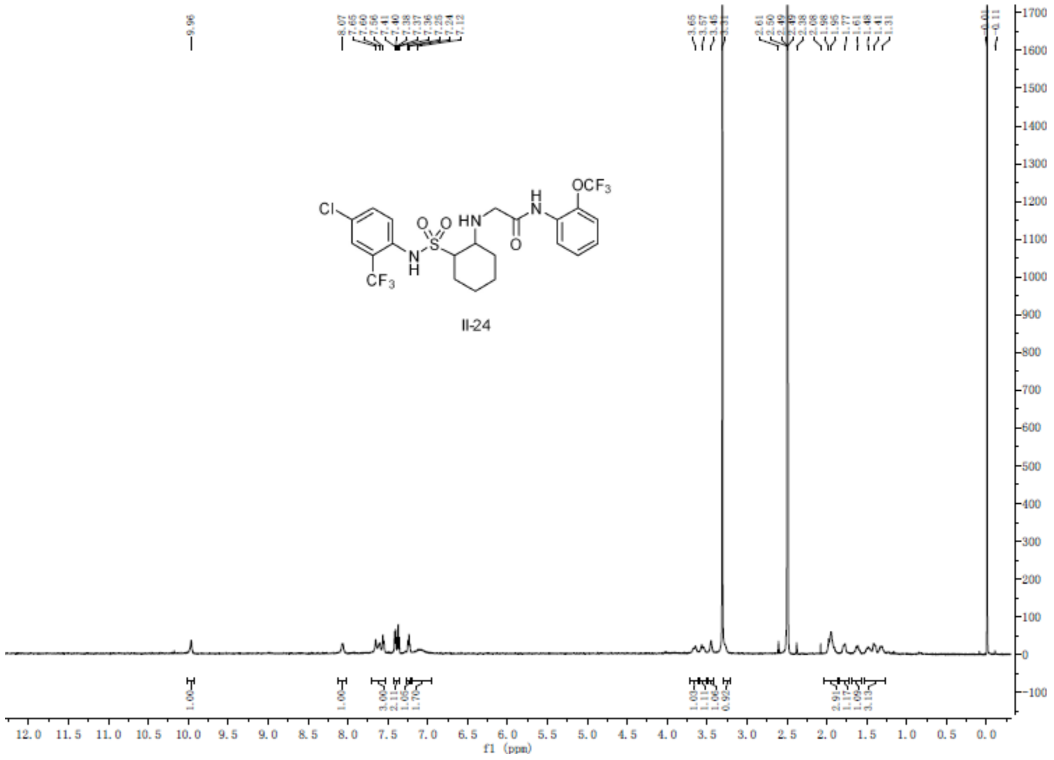

Figure S24-1 ^{1}H NMR spectrum of compound II-24.

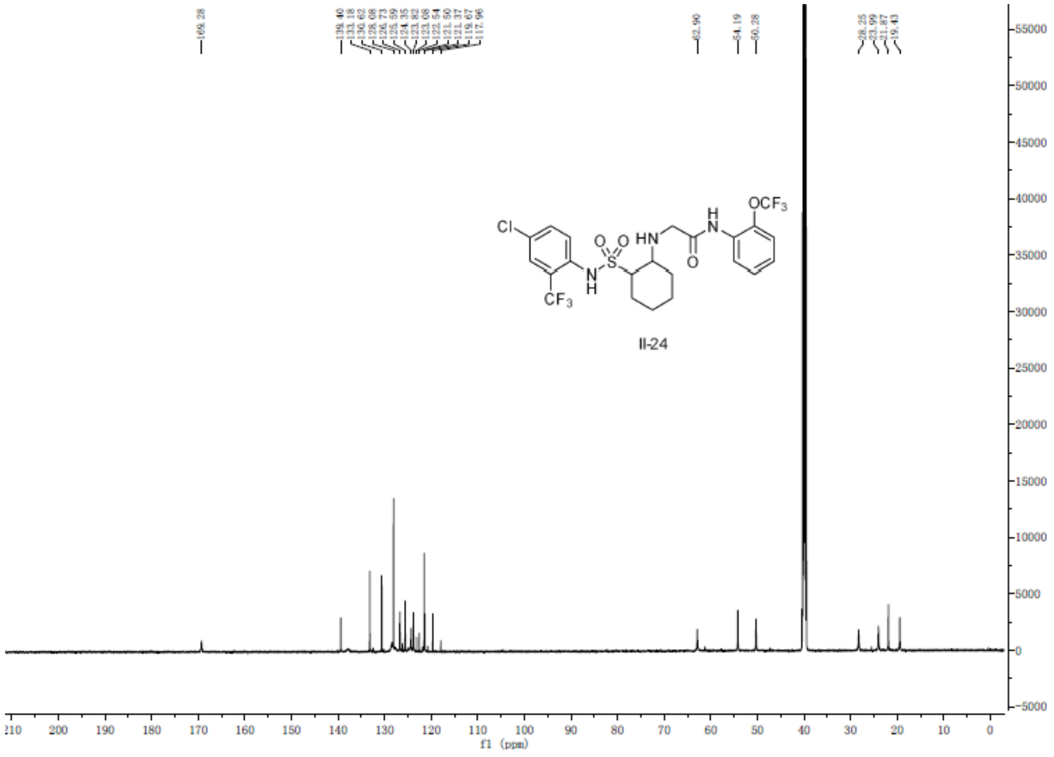

Figure S24-2 ^{13}C NMR spectrum of compound II-24.

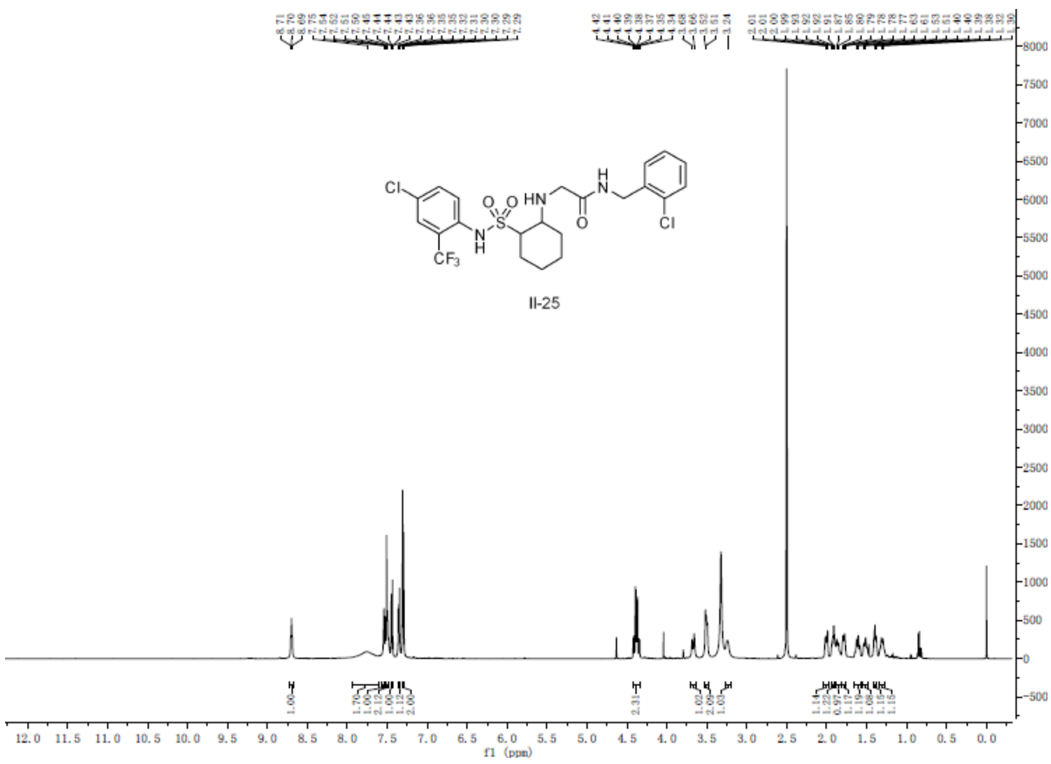

Figure S25-1 ^{1}H NMR spectrum of compound II-25.

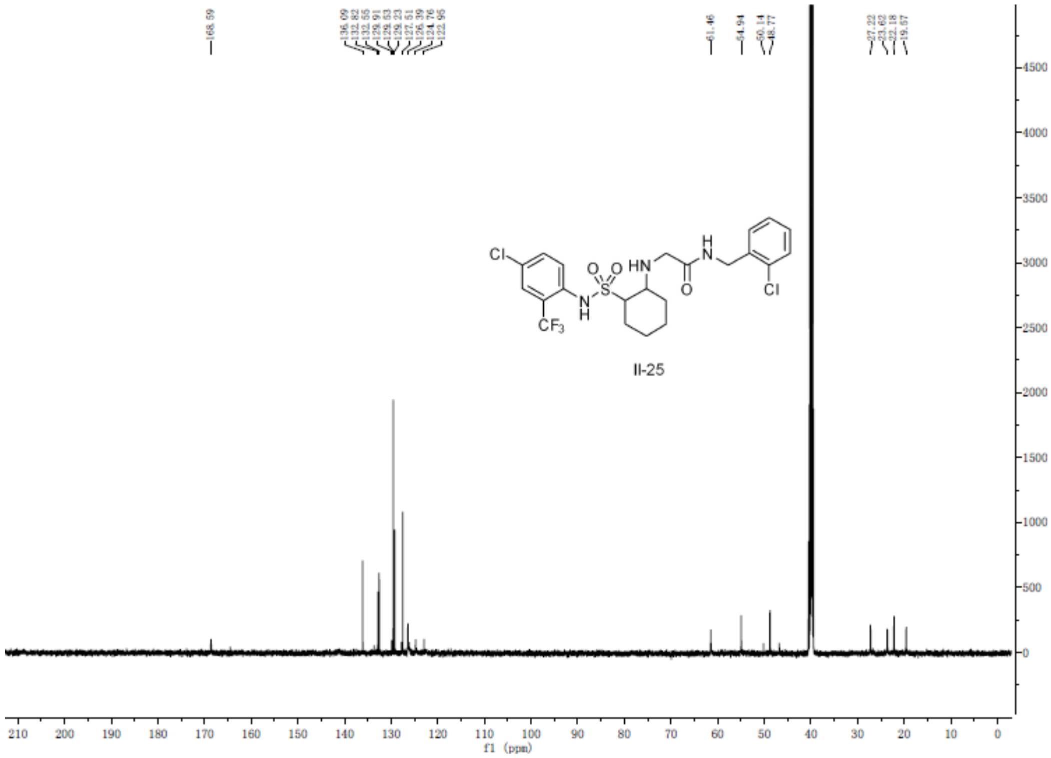

Figure S25-2 ^{13}C NMR spectrum of compound II-25.

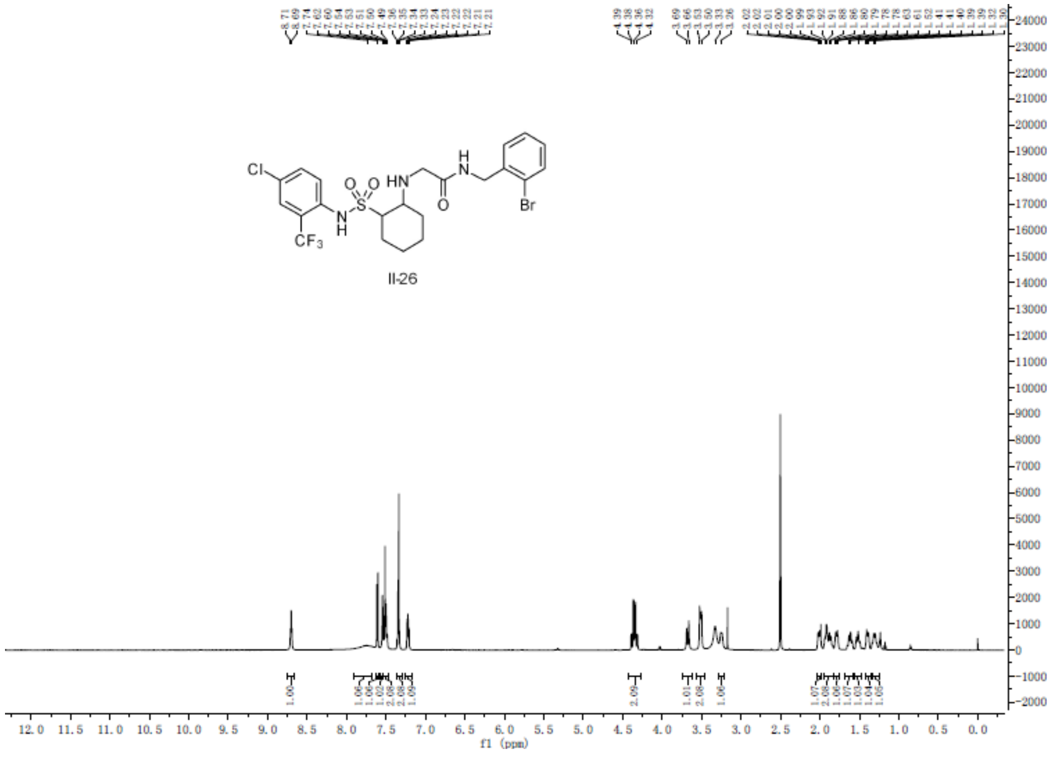

Figure S26-1 ^{1}H NMR spectrum of compound II-26.

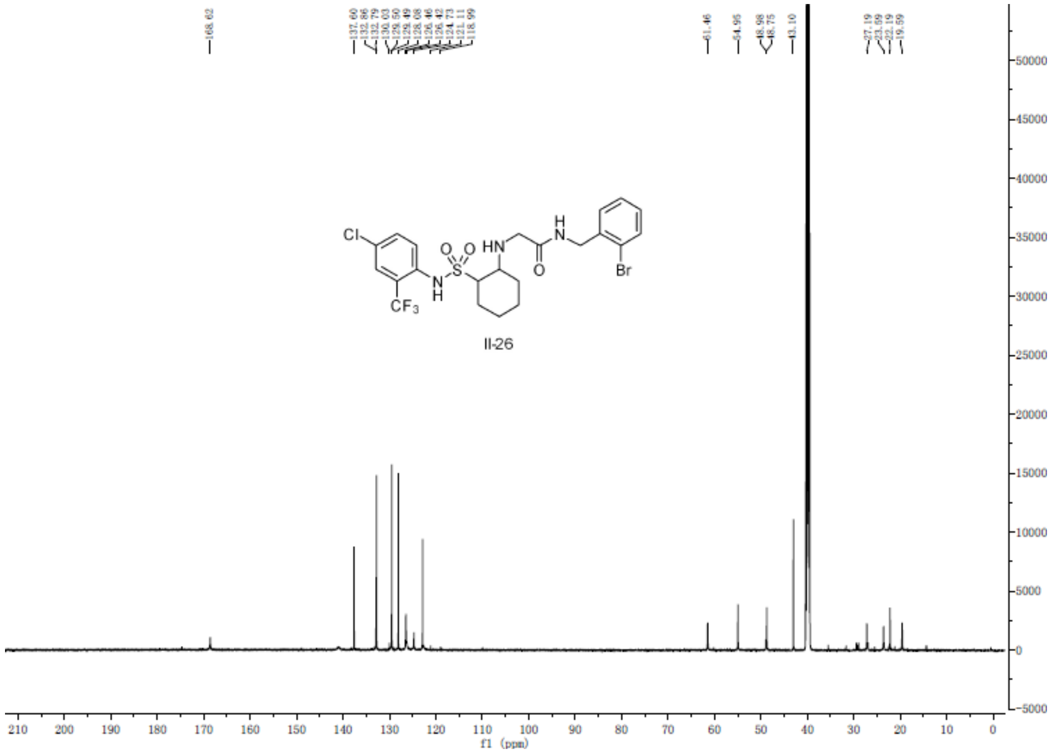

Figure S26-2 ^{13}C NMR spectrum of compound II-26.

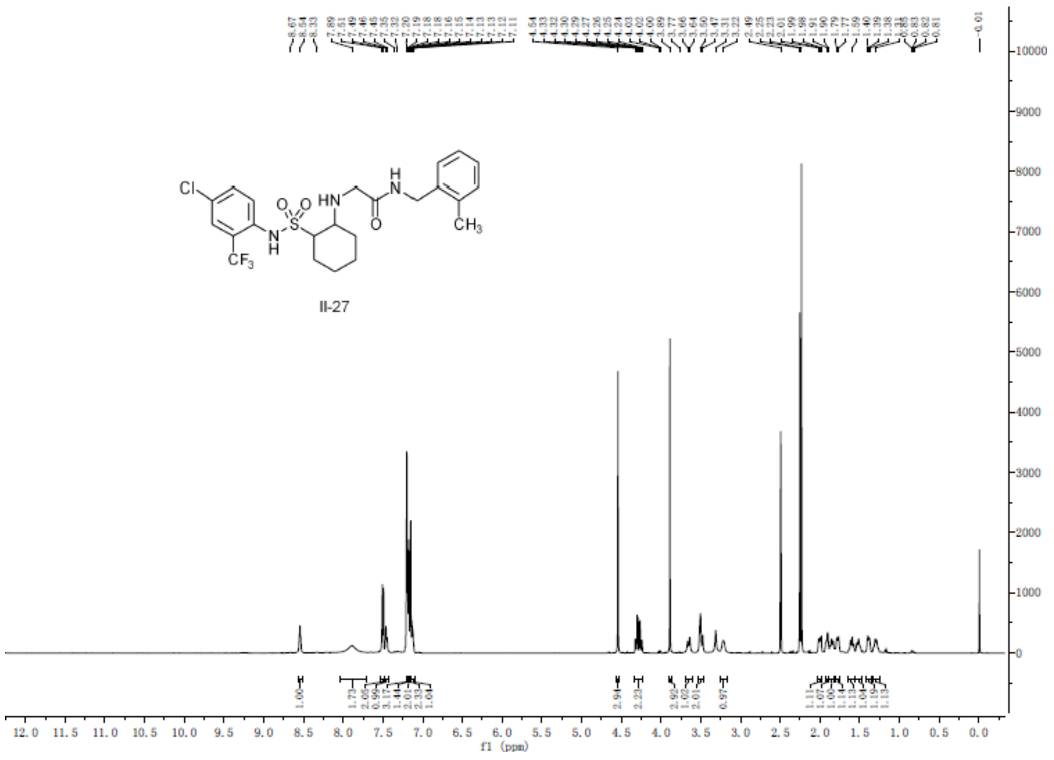

Figure S27-1 ^{1}H NMR spectrum of compound II-27.

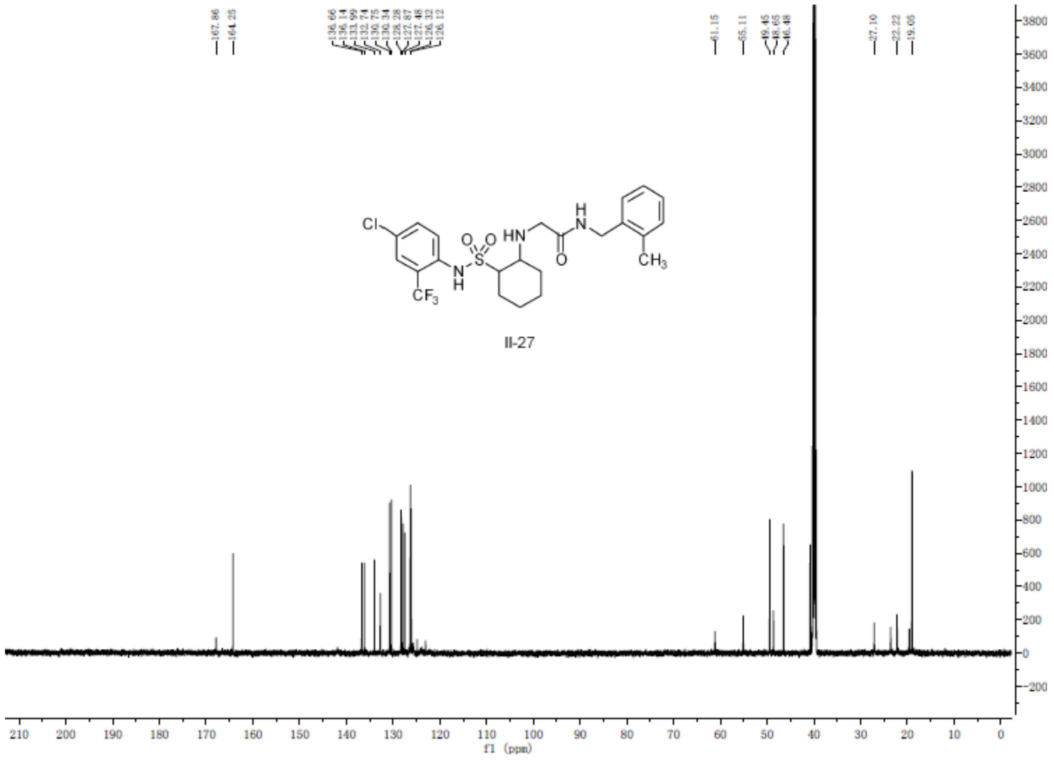

Figure S27-2 ^{13}C NMR spectrum of compound II-27.

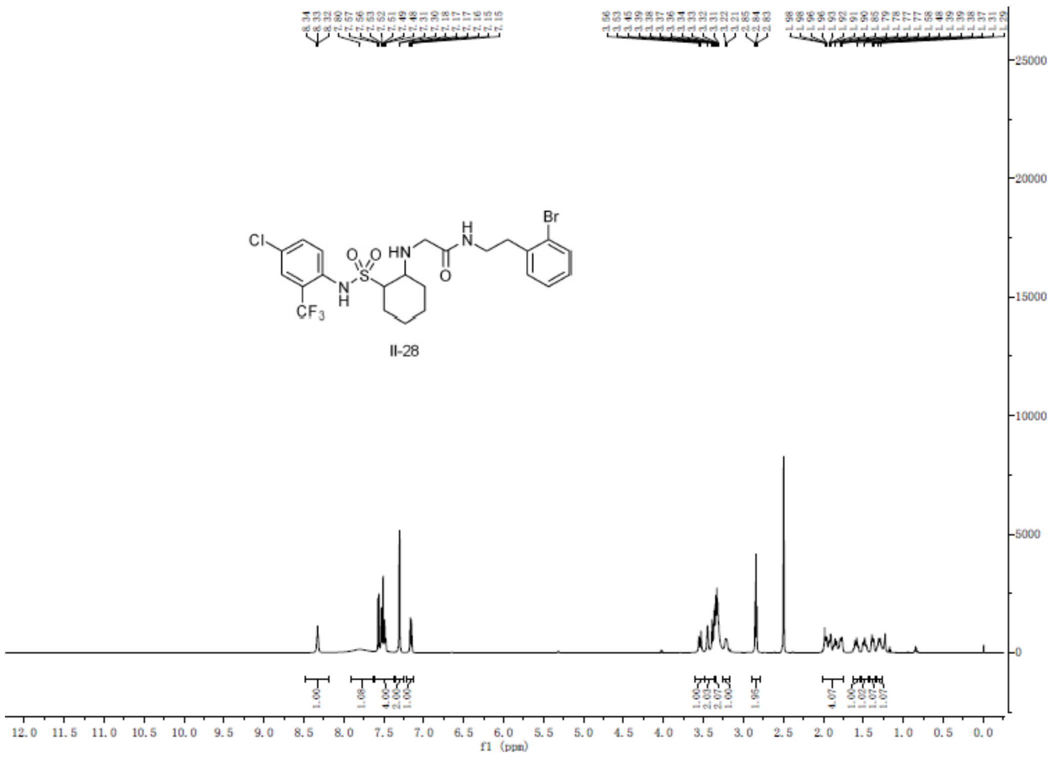

Figure S28-1 ^{1}H NMR spectrum of compound II-28.

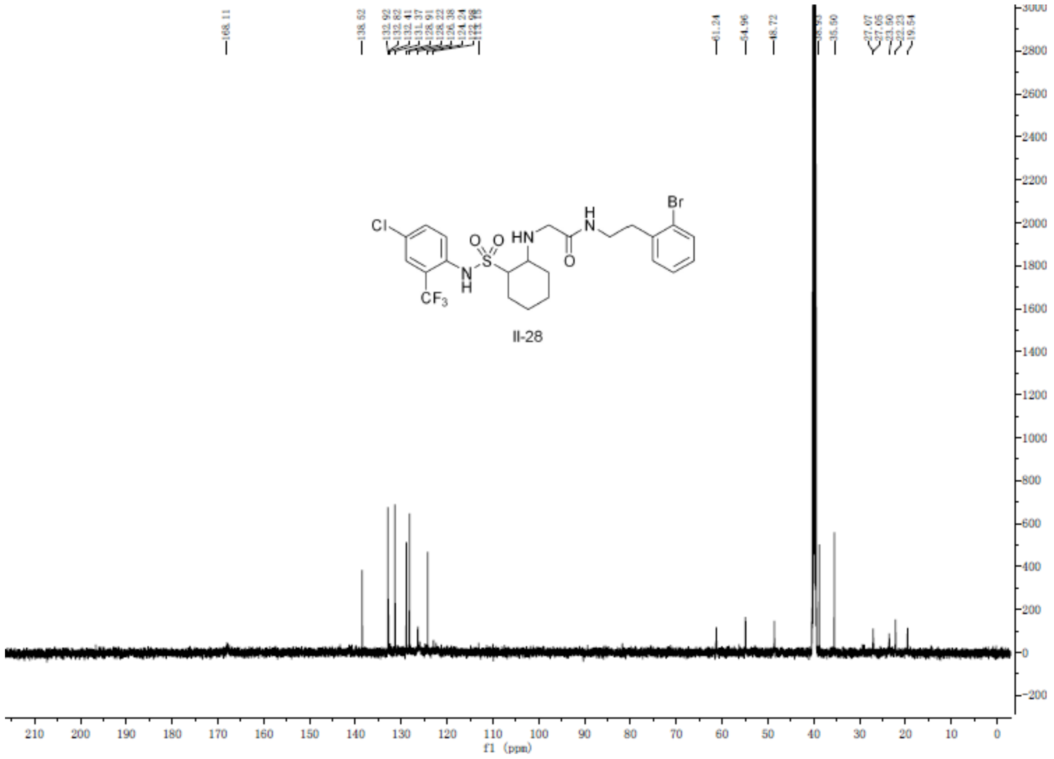

Figure S28-2 ^{13}C NMR spectrum of compound II-28.

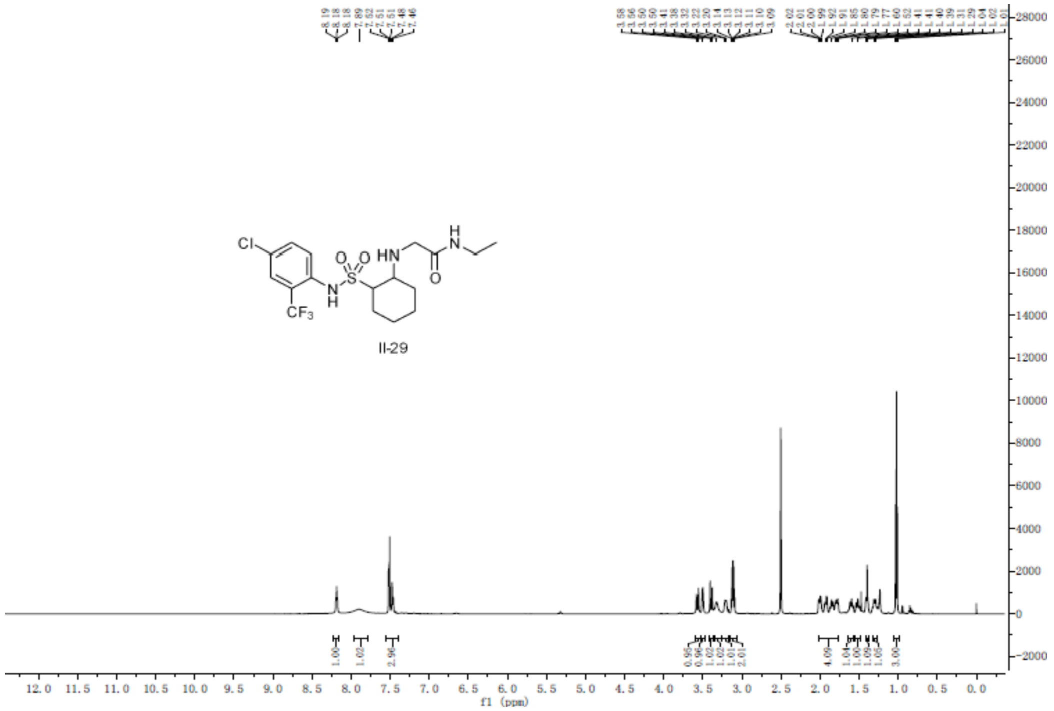

Figure S29-1 ^{1}H NMR spectrum of compound II-29.

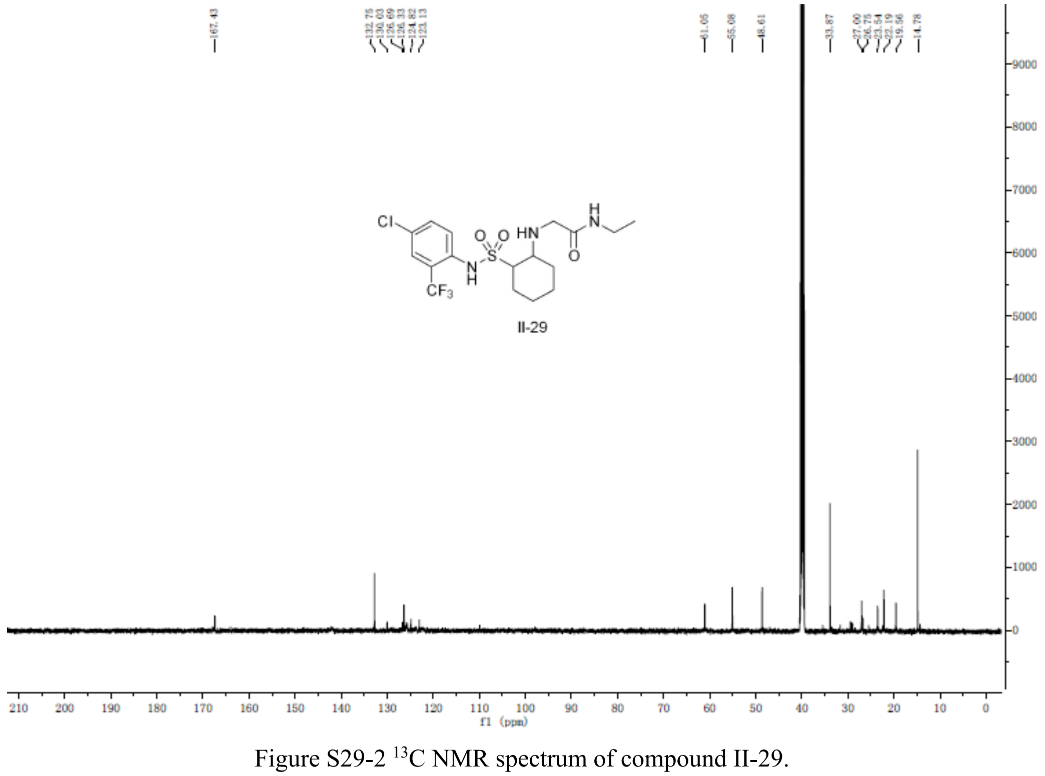

Figure S29-2 ^{13}C NMR spectrum of compound II-29.

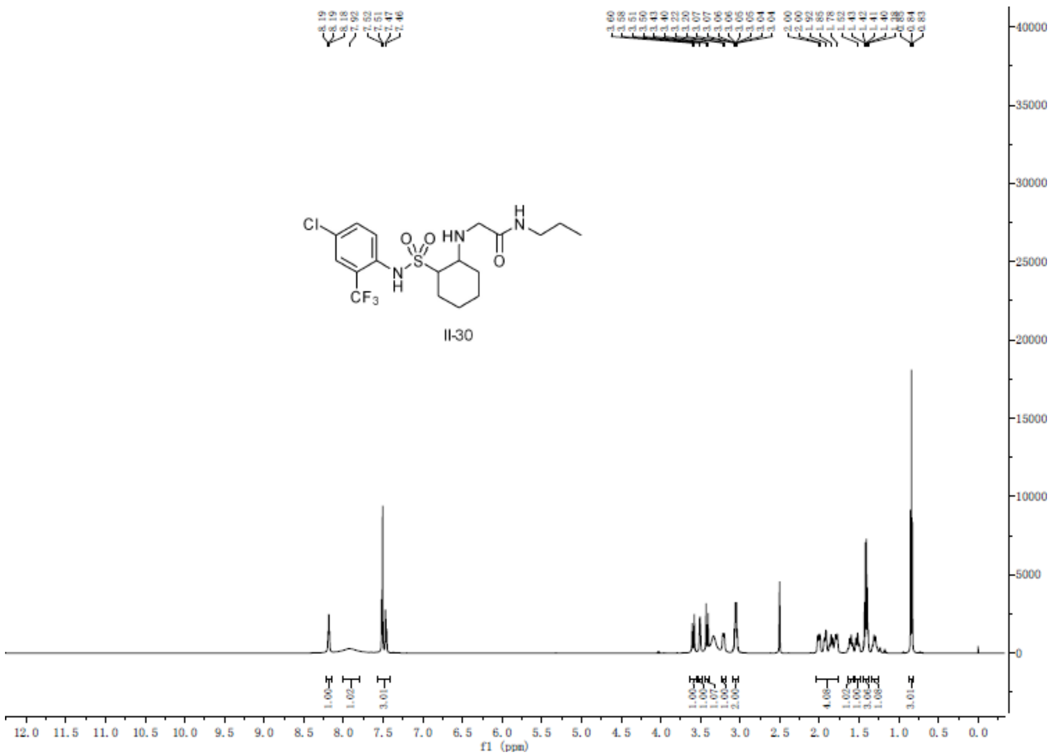

Figure S30-1 ^{1}H NMR spectrum of compound II-30.

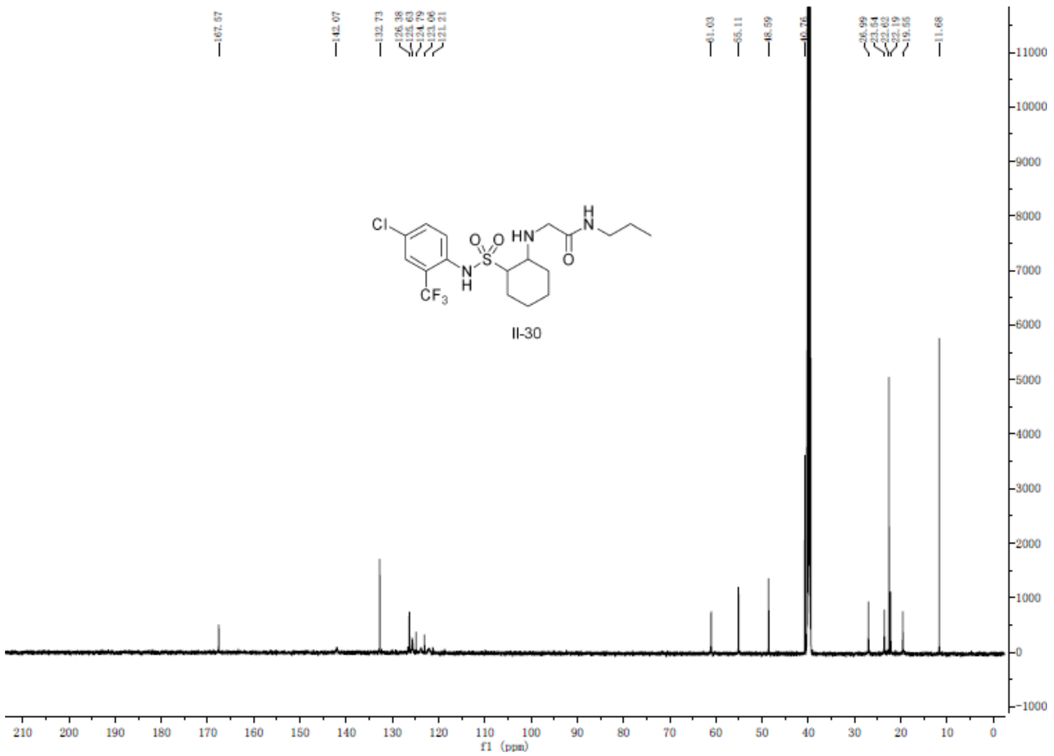

Figure S30-2 ^{13}C NMR spectrum of compound II-30.

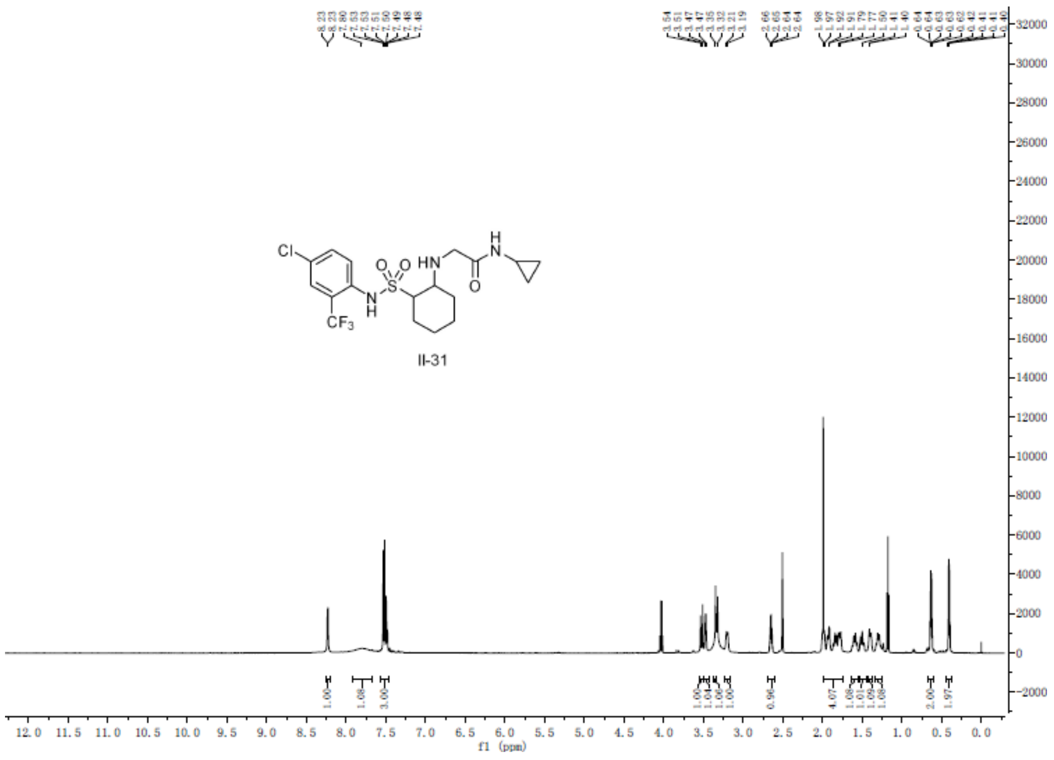

Figure S31-1 ^{1}H NMR spectrum of compound II-31.

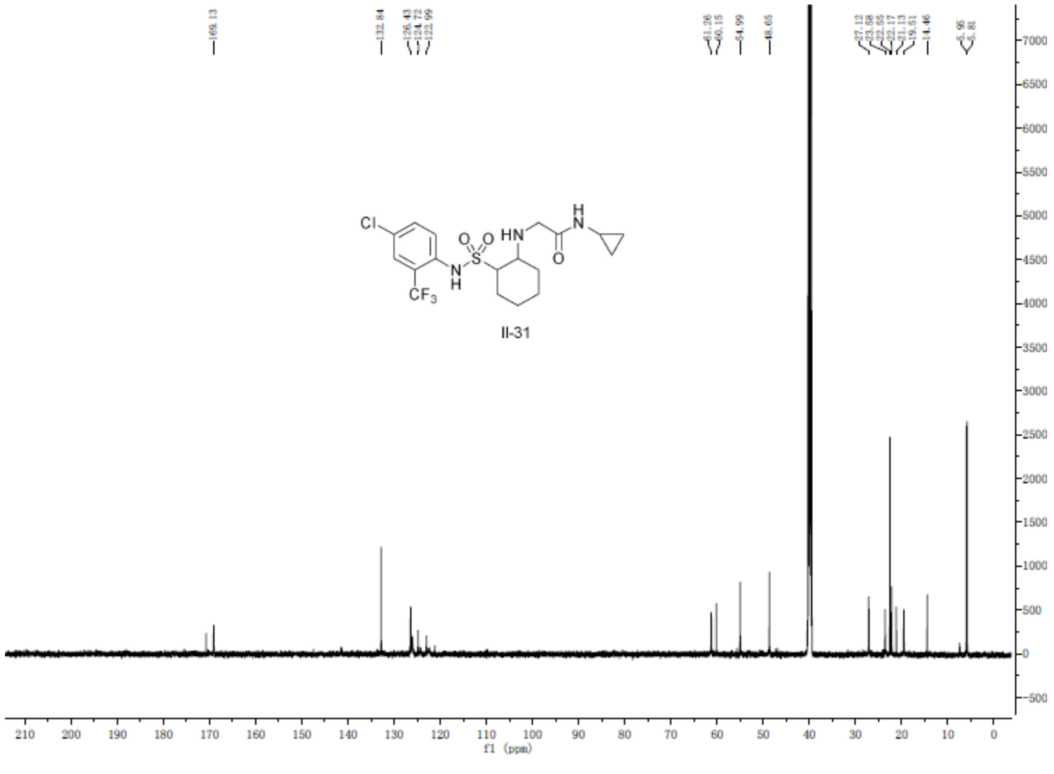

Figure S31-2 ^{13}C NMR spectrum of compound II-31.

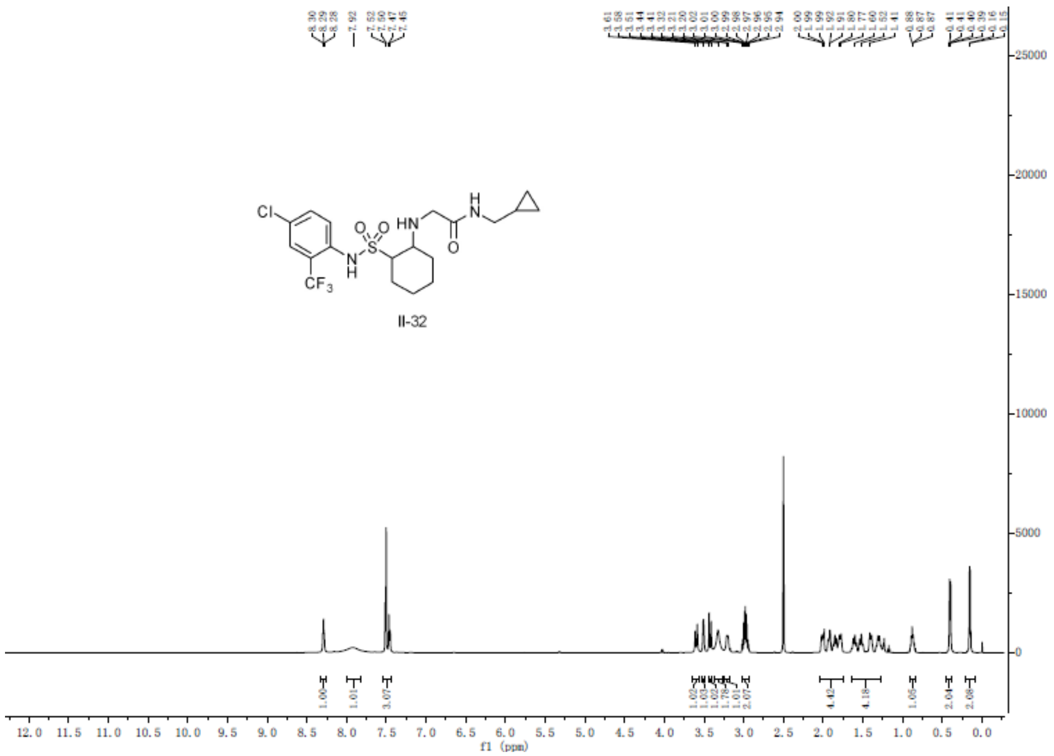

Figure S32-1 ^{1}H NMR spectrum of compound II-32.

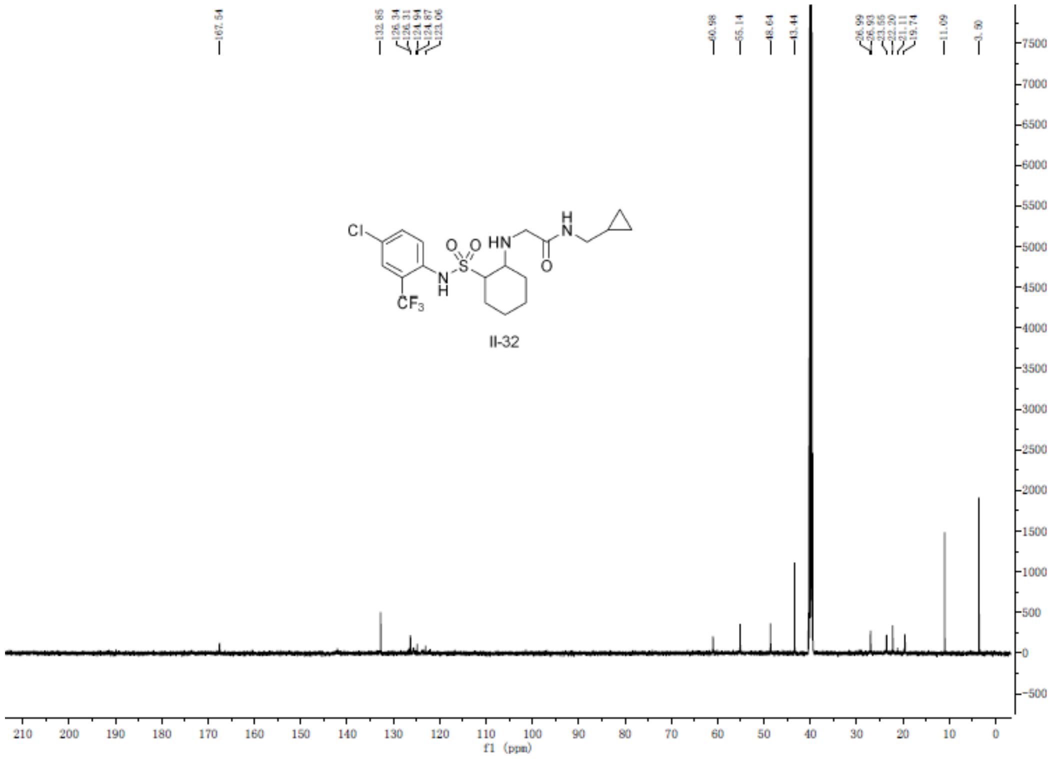

Figure S32-2 ^{13}C NMR spectrum of compound II-32.

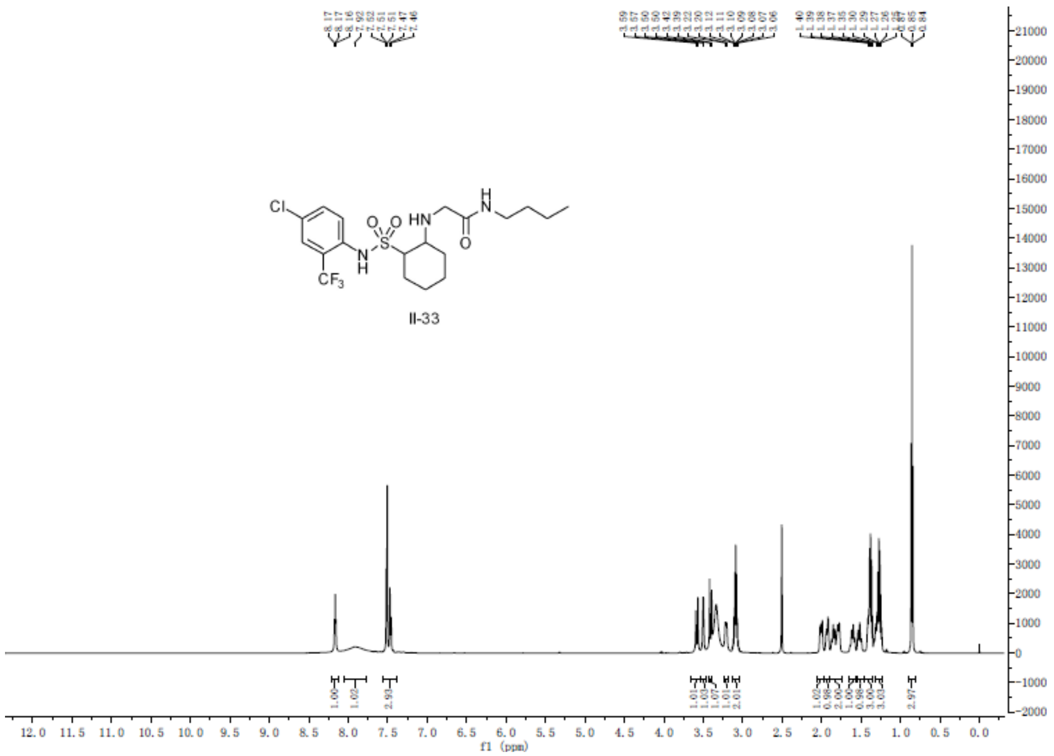

Figure S33-1 ^{1}H NMR spectrum of compound II-33.

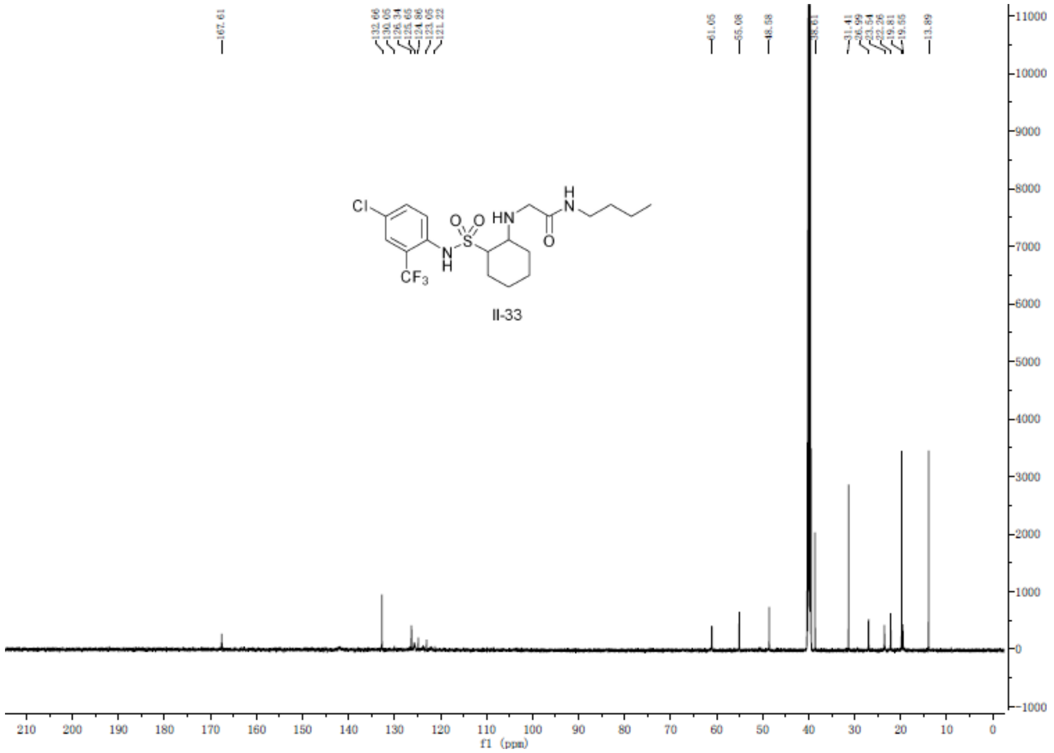

Figure S33-2 ^{13}C NMR spectrum of compound II-33.

Detailed description of the crystal structure of II-19.

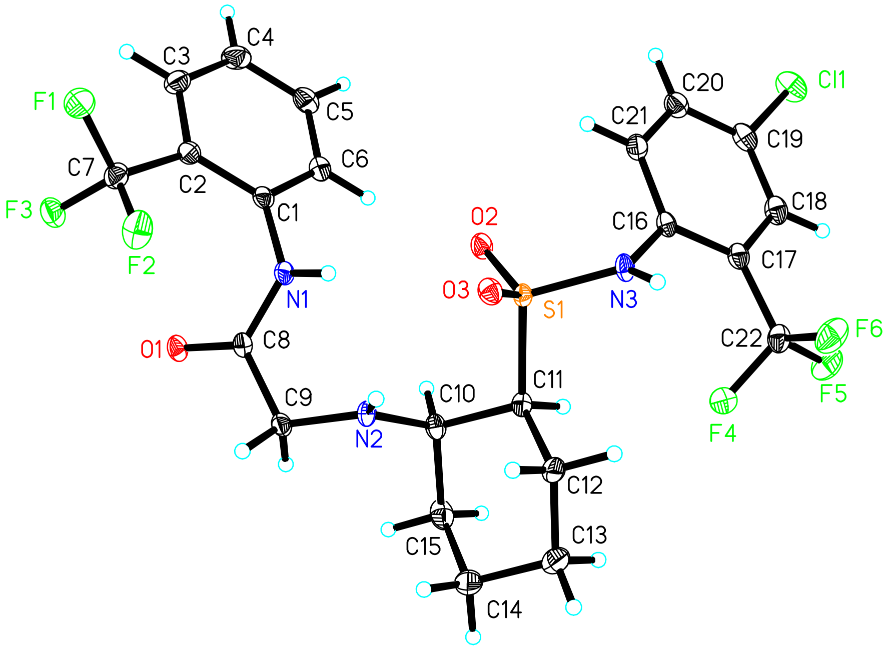

Fig.1 X-ray single crystal diffraction structure.

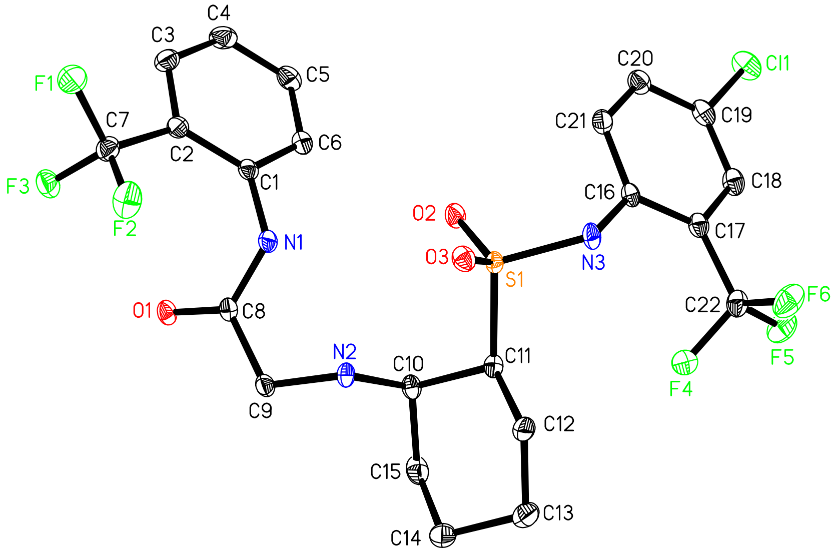

Fig.2 Three-dimensional ellipsoid diagram.

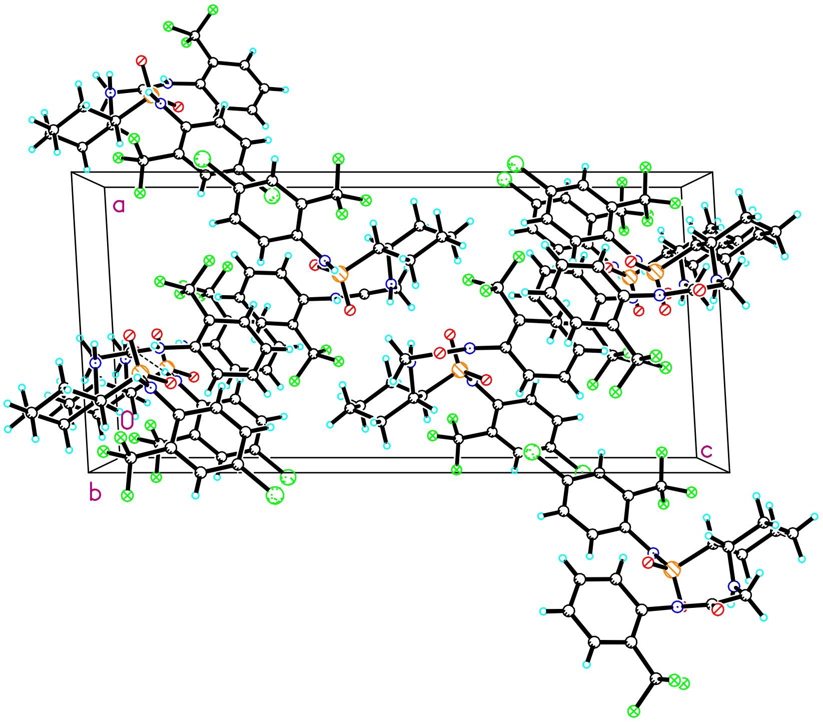

Fig. 3 Cell stacking diagram along the direction of b axis.

Table 1. Crystal data and structure refinement for II-19.

Identification code	II-19	
Empirical formula	$C_{22}H_{22}ClF_6N_3O_3S$	
Formula weight	557.94	
Temperature	173(2) K	
Wavelength	1.54178 A	
Crystal system, space group	Monoclinic, P 21/c	
Unit cell dimensions	a = 10.9396(3) A	alpha = 90 deg.
	b = 9.2428(3) A	beta = 93.2600(10)deg.
	c = 23.2957(7) A	gamma = 90 deg.
Volume	2351.68(12) A^3	
Z, Calculated density	4, 1.576 Mg/m^3	
Absorption coefficient	2.997 mm^-1	
F(000)	1144	
Crystal size	0.25 x 0.13 x 0.12 mm	
Theta range for data collection	3.80 to 68.27 deg.	
Limiting indices	-12<=h<=13, -11<=k<=10, -27<=l<=28	
Reflections collected / unique	21223 / 4279 [R(int) = 0.0221]	

Completeness to theta = 68.27	99.30%
Absorption correction	Semi-empirical from equivalents
Max. and min. transmission	0.7531 and 0.5374
Refinement method	Full-matrix least-squares on F^2
Data / restraints / parameters	4279 / 0 / 325
Goodness-of-fit on F^2	1.062
Final R indices [I>2sigma(I)]	R1 = 0.0339, wR2 = 0.0859
R indices (all data)	R1 = 0.0362, wR2 = 0.0875
Largest diff. peak and hole	0.653 and -0.725 e.A^-3

Table 2. Atomic coordinates (x 10^4) and equivalent isotropic displacement parameters (A^2 x 10^3) for II-19.

	x	y	z	U(eq)
Cl(1)	10503(1)	8906(1)	2019(1)	41(1)
S(1)	6684(1)	5948(1)	4059(1)	19(1)
F(1)	2823(1)	-236(2)	3099(1)	46(1)
F(2)	3571(1)	649(1)	3895(1)	42(1)
F(3)	3965(1)	-1536(1)	3663(1)	36(1)
F(4)	9125(1)	8549(1)	4560(1)	47(1)
F(5)	10443(1)	9869(1)	4175(1)	44(1)
F(6)	8589(1)	10589(1)	4188(1)	45(1)
O(1)	6013(1)	-289(1)	4415(1)	25(1)
O(2)	7019(1)	4888(1)	3644(1)	27(1)
O(3)	5428(1)	6085(1)	4189(1)	25(1)
N(1)	5876(1)	1826(2)	3921(1)	23(1)
N(2)	6373(1)	3433(1)	4880(1)	20(1)
N(3)	7108(1)	7536(1)	3843(1)	23(1)
C(1)	5843(2)	1221(2)	3358(1)	22(1)
C(2)	4923(2)	292(2)	3137(1)	24(1)
C(3)	4984(2)	-232(2)	2578(1)	32(1)
C(4)	5924(2)	168(2)	2238(1)	36(1)
C(5)	6827(2)	1092(2)	2456(1)	33(1)
C(6)	6787(2)	1607(2)	3012(1)	28(1)
C(7)	3840(2)	-194(2)	3455(1)	28(1)
C(8)	6036(2)	1044(2)	4406(1)	21(1)
C(9)	6243(2)	1882(2)	4959(1)	23(1)
C(10)	7625(2)	3980(2)	4856(1)	22(1)
C(11)	7587(2)	5608(2)	4714(1)	21(1)
C(12)	7148(2)	6536(2)	5207(1)	27(1)
C(13)	7965(2)	6278(2)	5752(1)	40(1)
C(14)	8008(2)	4677(2)	5908(1)	37(1)
C(15)	8435(2)	3774(2)	5411(1)	33(1)

C(16)	7975(2)	7812(2)	3426(1)	22(1)
C(17)	8998(2)	8713(2)	3540(1)	25(1)
C(18)	9770(2)	9041(2)	3103(1)	30(1)
C(19)	9537(2)	8463(2)	2561(1)	29(1)
C(20)	8547(2)	7564(2)	2443(1)	30(1)
C(21)	7771(2)	7255(2)	2875(1)	27(1)
C(22)	9282(2)	9417(2)	4114(1)	29(1)
H(1A)	5787	2768	3954	28
H(2B)	5734	4011	4847	24
H(3B)	6769	8292	4000	27
H(3C)	4369	-876	2427	38
H(4A)	5948	-190	1857	43
H(5A)	7475	1373	2225	40
H(6A)	7415	2236	3161	34
H(9A)	5545	1704	5203	27
H(9B)	6991	1510	5168	27
H(10A)	8022	3467	4538	27
H(11A)	8444	5913	4648	25
H(12A)	7175	7571	5099	33
H(12B)	6291	6285	5279	33
H(13A)	8804	6625	5691	48
H(13B)	7646	6837	6074	48
H(14A)	8574	4532	6250	45
H(14B)	7183	4353	6007	45
H(15A)	8431	2740	5521	39
H(15B)	9287	4044	5337	39
H(18A)	10454	9661	3177	36
H(20A)	8401	7163	2070	36
H(21A)	7083	6647	2793	32

Table 3. Bond lengths [A] and angles [deg] for II-19.

Cl(1)-C(19)	1.7402(18)	C(6)-H(6A)	0.95
S(1)-O(3)	1.4296(12)	C(8)-C(9)	1.509(2)
S(1)-O(2)	1.4390(12)	C(9)-H(9A)	0.99
S(1)-N(3)	1.6271(14)	C(9)-H(9B)	0.99
S(1)-C(11)	1.7989(16)	C(10)-C(15)	1.537(2)
F(1)-C(7)	1.350(2)	C(10)-C(11)	1.541(2)
F(2)-C(7)	1.333(2)	C(10)-H(10A)	1
F(3)-C(7)	1.335(2)	C(11)-C(12)	1.531(2)
F(4)-C(22)	1.333(2)	C(11)-H(11A)	1
F(5)-C(22)	1.337(2)	C(12)-C(13)	1.529(3)

F(6)-C(22)	1.339(2)	C(12)-H(12A)	0.99
O(1)-C(8)	1.232(2)	C(12)-H(12B)	0.99
N(1)-C(8)	1.345(2)	C(13)-C(14)	1.524(3)
N(1)-C(1)	1.425(2)	C(13)-H(13A)	0.99
N(1)-H(1A)	0.88	C(13)-H(13B)	0.99
N(2)-C(9)	1.453(2)	C(14)-C(15)	1.523(3)
N(2)-C(10)	1.464(2)	C(14)-H(14A)	0.99
N(2)-H(2B)	0.88	C(14)-H(14B)	0.99
N(3)-C(16)	1.419(2)	C(15)-H(15A)	0.99
N(3)-H(3B)	0.88	C(15)-H(15B)	0.99
C(1)-C(6)	1.392(2)	C(16)-C(21)	1.389(2)
C(1)-C(2)	1.398(2)	C(16)-C(17)	1.408(2)
C(2)-C(3)	1.395(2)	C(17)-C(18)	1.394(2)
C(2)-C(7)	1.502(2)	C(17)-C(22)	1.503(3)
C(3)-C(4)	1.383(3)	C(18)-C(19)	1.380(3)
C(3)-H(3C)	0.95	C(18)-H(18A)	0.95
C(4)-C(5)	1.381(3)	C(19)-C(20)	1.381(3)
C(4)-H(4A)	0.95	C(20)-C(21)	1.383(3)
C(5)-C(6)	1.383(3)	C(20)-H(20A)	0.95
C(5)-H(5A)	0.95	C(21)-H(21A)	0.95
O(3)-S(1)-O(2)	119.15(7)	C(12)-C(11)-C(10)	113.09(14)
O(3)-S(1)-N(3)	106.28(7)	C(12)-C(11)-S(1)	110.99(11)
O(2)-S(1)-N(3)	108.56(7)	C(10)-C(11)-S(1)	111.07(11)
O(3)-S(1)-C(11)	108.89(7)	C(12)-C(11)-H(11A)	107.1
O(2)-S(1)-C(11)	107.65(8)	C(10)-C(11)-H(11A)	107.1
N(3)-S(1)-C(11)	105.51(8)	S(1)-C(11)-H(11A)	107.1
C(8)-N(1)-C(1)	123.90(14)	C(13)-C(12)-C(11)	109.98(15)
C(8)-N(1)-H(1A)	118	C(13)-C(12)-H(12A)	109.7
C(1)-N(1)-H(1A)	118	C(11)-C(12)-H(12A)	109.7
C(9)-N(2)-C(10)	116.38(13)	C(13)-C(12)-H(12B)	109.7
C(9)-N(2)-H(2B)	121.8	C(11)-C(12)-H(12B)	109.7
C(10)-N(2)-H(2B)	121.8	H(12A)-C(12)-H(12B)	108.2
C(16)-N(3)-S(1)	125.94(11)	C(14)-C(13)-C(12)	111.00(15)
C(16)-N(3)-H(3B)	117	C(14)-C(13)-H(13A)	109.4
S(1)-N(3)-H(3B)	117	C(12)-C(13)-H(13A)	109.4
C(6)-C(1)-C(2)	119.04(16)	C(14)-C(13)-H(13B)	109.4
C(6)-C(1)-N(1)	116.81(15)	C(12)-C(13)-H(13B)	109.4
C(2)-C(1)-N(1)	124.14(15)	H(13A)-C(13)-H(13B)	108
C(3)-C(2)-C(1)	119.09(16)	C(15)-C(14)-C(13)	110.90(17)
C(3)-C(2)-C(7)	116.00(16)	C(15)-C(14)-H(14A)	109.5
C(1)-C(2)-C(7)	124.91(15)	C(13)-C(14)-H(14A)	109.5
C(4)-C(3)-C(2)	121.20(18)	C(15)-C(14)-H(14B)	109.5
C(4)-C(3)-H(3C)	119.4	C(13)-C(14)-H(14B)	109.5
C(2)-C(3)-H(3C)	119.4	H(14A)-C(14)-H(14B)	108

C(5)-C(4)-C(3)	119.63(17)	C(14)-C(15)-C(10)	112.65(15)
C(5)-C(4)-H(4A)	120.2	C(14)-C(15)-H(15A)	109.1
C(3)-C(4)-H(4A)	120.2	C(10)-C(15)-H(15A)	109.1
C(4)-C(5)-C(6)	119.82(18)	C(14)-C(15)-H(15B)	109.1
C(4)-C(5)-H(5A)	120.1	C(10)-C(15)-H(15B)	109.1
C(6)-C(5)-H(5A)	120.1	H(15A)-C(15)-H(15B)	107.8
C(5)-C(6)-C(1)	121.22(17)	C(21)-C(16)-C(17)	118.57(16)
C(5)-C(6)-H(6A)	119.4	C(21)-C(16)-N(3)	119.22(15)
C(1)-C(6)-H(6A)	119.4	C(17)-C(16)-N(3)	122.08(15)
F(2)-C(7)-F(3)	106.69(15)	C(18)-C(17)-C(16)	119.88(16)
F(2)-C(7)-F(1)	106.15(15)	C(18)-C(17)-C(22)	116.92(16)
F(3)-C(7)-F(1)	105.08(14)	C(16)-C(17)-C(22)	123.13(15)
F(2)-C(7)-C(2)	114.80(15)	C(19)-C(18)-C(17)	119.76(17)
F(3)-C(7)-C(2)	112.92(15)	C(19)-C(18)-H(18A)	120.1
F(1)-C(7)-C(2)	110.52(15)	C(17)-C(18)-H(18A)	120.1
O(1)-C(8)-N(1)	123.37(15)	C(18)-C(19)-C(20)	121.16(16)
O(1)-C(8)-C(9)	120.10(15)	C(18)-C(19)-Cl(1)	118.92(14)
N(1)-C(8)-C(9)	116.53(14)	C(20)-C(19)-Cl(1)	119.92(15)
N(2)-C(9)-C(8)	114.16(13)	C(19)-C(20)-C(21)	119.08(17)
N(2)-C(9)-H(9A)	108.7	C(19)-C(20)-H(20A)	120.5
C(8)-C(9)-H(9A)	108.7	C(21)-C(20)-H(20A)	120.5
N(2)-C(9)-H(9B)	108.7	C(20)-C(21)-C(16)	121.54(16)
C(8)-C(9)-H(9B)	108.7	C(20)-C(21)-H(21A)	119.2
H(9A)-C(9)-H(9B)	107.6	C(16)-C(21)-H(21A)	119.2
N(2)-C(10)-C(15)	114.95(14)	F(4)-C(22)-F(5)	105.60(15)
N(2)-C(10)-C(11)	109.36(13)	F(4)-C(22)-F(6)	106.68(16)
C(15)-C(10)-C(11)	107.96(14)	F(5)-C(22)-F(6)	105.96(15)
N(2)-C(10)-H(10A)	108.1	F(4)-C(22)-C(17)	113.82(15)
C(15)-C(10)-H(10A)	108.1	F(5)-C(22)-C(17)	112.12(15)
C(11)-C(10)-H(10A)	108.1	F(6)-C(22)-C(17)	112.09(15)

Table 4. Torsion angles [deg] for II-19.

O(3)-S(1)-N(3)-C(16)	-147.07(14)
O(2)-S(1)-N(3)-C(16)	-17.76(16)
C(11)-S(1)-N(3)-C(16)	97.39(15)
C(8)-N(1)-C(1)-C(6)	-114.54(18)
C(8)-N(1)-C(1)-C(2)	66.2(2)
C(6)-C(1)-C(2)-C(3)	0.6(2)
N(1)-C(1)-C(2)-C(3)	179.83(15)
C(6)-C(1)-C(2)-C(7)	-178.81(16)
N(1)-C(1)-C(2)-C(7)	0.4(3)
C(1)-C(2)-C(3)-C(4)	-1.0(3)

C(7)-C(2)-C(3)-C(4)	178.46(17)
C(2)-C(3)-C(4)-C(5)	0.6(3)
C(3)-C(4)-C(5)-C(6)	0.1(3)
C(4)-C(5)-C(6)-C(1)	-0.5(3)
C(2)-C(1)-C(6)-C(5)	0.2(3)
N(1)-C(1)-C(6)-C(5)	-179.14(16)
C(3)-C(2)-C(7)-F(2)	-158.81(16)
C(1)-C(2)-C(7)-F(2)	20.6(2)
C(3)-C(2)-C(7)-F(3)	78.58(19)
C(1)-C(2)-C(7)-F(3)	-102.00(19)
C(3)-C(2)-C(7)-F(1)	-38.8(2)
C(1)-C(2)-C(7)-F(1)	140.61(17)
C(1)-N(1)-C(8)-O(1)	-9.6(3)
C(1)-N(1)-C(8)-C(9)	170.83(15)
C(10)-N(2)-C(9)-C(8)	-94.13(17)
O(1)-C(8)-C(9)-N(2)	173.98(15)
N(1)-C(8)-C(9)-N(2)	-6.4(2)
C(9)-N(2)-C(10)-C(15)	-63.67(19)
C(9)-N(2)-C(10)-C(11)	174.73(13)
N(2)-C(10)-C(11)-C(12)	69.91(17)
C(15)-C(10)-C(11)-C(12)	-55.82(18)
N(2)-C(10)-C(11)-S(1)	-55.65(16)
C(15)-C(10)-C(11)-S(1)	178.63(12)
O(3)-S(1)-C(11)-C(12)	-40.70(14)
O(2)-S(1)-C(11)-C(12)	-171.18(11)
N(3)-S(1)-C(11)-C(12)	73.04(13)
O(3)-S(1)-C(11)-C(10)	86.02(12)
O(2)-S(1)-C(11)-C(10)	-44.46(13)
N(3)-S(1)-C(11)-C(10)	-160.24(11)
C(10)-C(11)-C(12)-C(13)	56.95(19)
S(1)-C(11)-C(12)-C(13)	-177.45(13)
C(11)-C(12)-C(13)-C(14)	-55.8(2)
C(12)-C(13)-C(14)-C(15)	56.3(2)
C(13)-C(14)-C(15)-C(10)	-57.2(2)
N(2)-C(10)-C(15)-C(14)	-66.81(19)
C(11)-C(10)-C(15)-C(14)	55.54(19)
S(1)-N(3)-C(16)-C(21)	58.5(2)
S(1)-N(3)-C(16)-C(17)	-125.71(15)

C(21)-C(16)-C(17)-C(18)	0.8(2)
N(3)-C(16)-C(17)-C(18)	-174.97(15)
C(21)-C(16)-C(17)-C(22)	177.75(16)
N(3)-C(16)-C(17)-C(22)	1.9(3)
C(16)-C(17)-C(18)-C(19)	-1.0(3)
C(22)-C(17)-C(18)-C(19)	-178.06(16)
C(17)-C(18)-C(19)-C(20)	0.2(3)
C(17)-C(18)-C(19)-Cl(1)	179.25(13)
C(18)-C(19)-C(20)-C(21)	0.8(3)
Cl(1)-C(19)-C(20)-C(21)	-178.31(14)
C(19)-C(20)-C(21)-C(16)	-0.9(3)
C(17)-C(16)-C(21)-C(20)	0.1(3)
N(3)-C(16)-C(21)-C(20)	176.02(16)
C(18)-C(17)-C(22)-F(4)	-140.52(17)
C(16)-C(17)-C(22)-F(4)	42.5(2)
C(18)-C(17)-C(22)-F(5)	-20.7(2)
C(16)-C(17)-C(22)-F(5)	162.28(16)
C(18)-C(17)-C(22)-F(6)	98.30(19)
C(16)-C(17)-C(22)-F(6)	-78.7(2)

Table 5. Hydrogen bonds for II-19 [A and deg.].

D-H...A	d(D-H)	d(H...A)	d(D...A)	<(DHA)
N(1)-H(1A)...O(2)	0.88	2.51	3.1757(18)	133.2
N(2)-H(2B)...O(3)	0.88	2.47	3.0790(18)	127.2
N(3)-H(3B)...O(1)#1	0.88	1.85	2.7265(18)	171.8

Symmetry transformations used to generate equivalent atoms: #1 x, y+1, z
